# Protein Language Model‐Guided Engineering of a 2,3‐Butanediol Dehydrogenase for the Enantioselective Synthesis of Cyclic *α*‐Hydroxy Ketones

**DOI:** 10.1002/advs.202509314

**Published:** 2026-01-25

**Authors:** Haote Ding, Ling Jiang, Yijia Song, Zhongji Pu, Lirong Yang, Haoran Yu

**Affiliations:** ^1^ Institute of Bioengineering College of Chemical and Biological Engineering Zhejiang University Hangzhou China; ^2^ ZJU‐Hangzhou Global Scientific and Technological Innovation Centre Hangzhou China; ^3^ Zhejiang Key Laboratory of Intelligent Manufacturing for Functional Chemicals ZJU‐Hangzhou Global Scientific and Technological Innovation Centre Zhejiang University Hangzhou China

**Keywords:** 2,3‐butanediol dehydrogenase, non‐canonical amino acid, protein engineering, α‐hydroxy ketone

## Abstract

(2*R*,3*R*)‐butanediol dehydrogenases (BDHs) are promising catalysts for the production of *α*‐hydroxy ketones, which are highly valuable compounds in the synthesis of fine chemicals and pharmaceuticals. However, (2*R*,3*R*)‐BDHs display limited stereoselectivity, thus restricting wider applications. In this study, we engineered a (2*R*,3*R*)‐BDH from *Bacillus subtilis* (*Bs*BDH) to enhance and invert its stereoselectivity toward 1,2‐cyclohexanediol (1,2‐CHD) for the production of chiral 2‐hydroxycyclohexanone. The hot spots 115, 118, 293 of *Bs*BDH were initially identified using the protein language model ESM‐1v. Subsequently, to obtain a stable scaffold to engineer stereoselectivity, we devised a strategy of position analysis and source search, achieving a true‐positive rate of 88.2% in designing thermostable single variants. Furthermore, iterative saturation mutagenesis was applied to the hot spots of the thermostable variant 6M2, and obtained a *trans*‐CHD preference variant LTF (*ee* > 99%) and a *cis*‐CHD preference variant 10M (*ee* > 99%). Several high‐activity variants were also obtained, including 6M2/F115C/L118F and 6M2/F115L/L118M, which demonstrated the activity improvements toward 25 substrates, with the highest enhancement reaching 5183.1‐fold. Additionally, molecular dynamics (MD) simulations and the incorporation of non‐canonical amino acids (ncAAs) were utilized to elucidate the mechanisms underlying the variants. The engineered *Bs*BDH variants exhibit promising potential for the biocatalytic production of *α*‐hydroxyketones.

## Graphical abstract

A (2*R*,3*R*)‐butanediol dehydrogenase from *Bacillus subtilis* (*Bs*BDH) is engineered for the enantioselective synthesis of 2‐hydroxycyclohexanone. A PASS computational design strategy is proposed to enhance the thermostability of *Bs*BDH. Moreover, ESM‐1v combined with ISM is utilized for enhancing and inverting its stereoselectivity. The engineered *Bs*BDH variants demonstrate promising potential for the biocatalytic production of *α*‐hydroxyketones in the future.

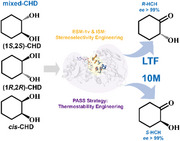

## 1 Introduction

**SCHEME 1:**
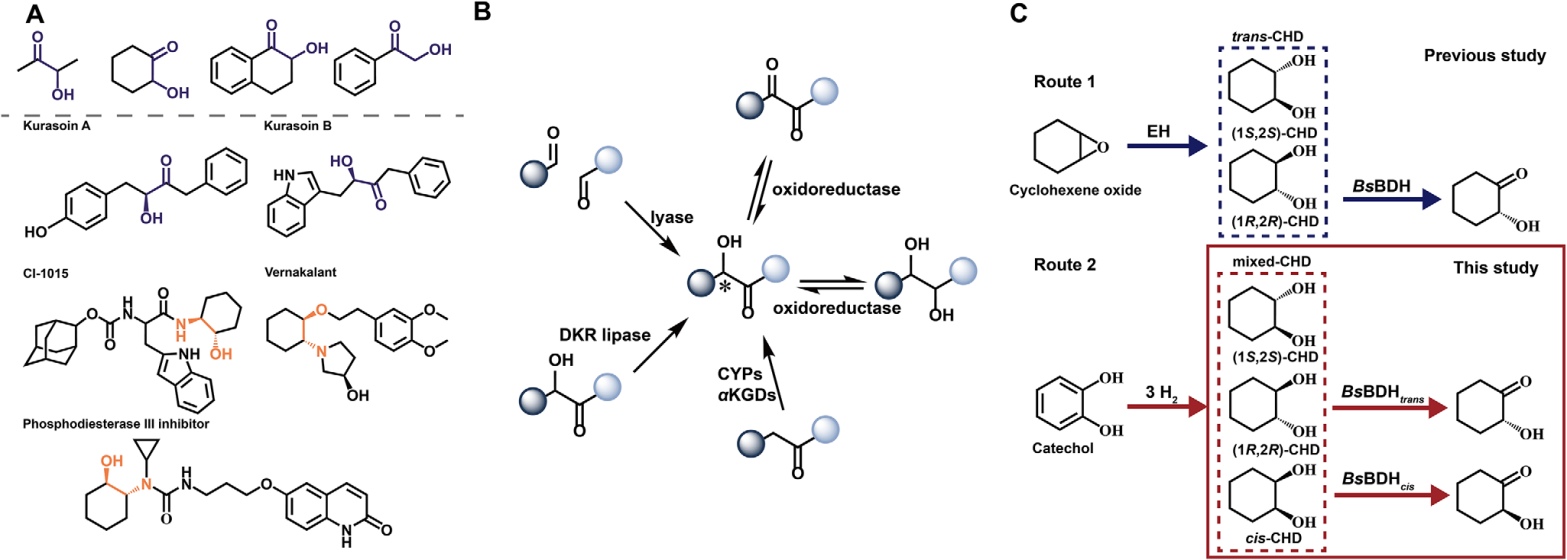
Important *α*‐hydroxy ketones and the synthetic strategies. (A) Important *α*‐hydroxy ketones, including intermediates and active pharmaceutical ingredients. (B) Biocatalytic strategies for the production of *α*‐hydroxy ketones. Aldol condensation via lyases; DKY (dynamic kinetic resolution) with lipases; *α*‐hydroxylation via cytochrome P450s (CYPs) and Fe(II)/*α*‐ketoglutarate‐dependent dioxygenases (*α*KGDs); reduction or oxidation catalyzed by oxidoreductases. (C) Synthetic strategies for synthesizing chiral 2‐hydroxycyclohexanone (HCH) as reported in the previous study and this work. Abbreviations: EH, epoxide hydrolase; *Bs*BDH*_trans_*, mutants of *Bs*BDH that only accept (1*R*,2*R*)‐CHD; *Bs*BDH*_cis_*, mutants of *Bs*BDH that only accept *cis*‐CHD.

Chiral *α*‐hydroxy ketones are valuable chiral auxiliaries, ligands, and templates in asymmetric reactions, and are essential building blocks in the synthesis of pharmaceuticals [1]. For instance, *α*‐hydroxy ketones are key components in various bioactive compounds, including antidepressants, selective amyloidogenesis inhibitors, farnesyl transferase inhibitors, antifungal agents, and antitumor antibiotics (Scheme 1A) [2, 3, 4]. Moreover, *α*‐hydroxy ketones are valuable fine chemicals for synthesizing other important molecules, such as amino alcohols, diols, and related derivatives (Scheme 1A) [5, 6]. For example, cyclic *α*‐hydroxy ketones are frequently utilized in the synthesis of cyclic *β*‐amino alcohols. They act as intermediates for certain pharmaceutical ingredients, such as “Peptoid” cholecystokinin (CCK‐B) receptor antagonists (CI‐1015) [7], phosphodiesterase III inhibitors, and the antiarrhythmic agent Vernakalant (Scheme 1A) [8]. Various chemical synthetic approaches have been developed to synthesize these compounds, including thiazolium‐based carboligation [9], proline‐catalyzed *α*‐hydroxylation [10], and methyl sulfonyl azide‐mediated C═C bond cleavage [11]. However, these chemical approaches often encounter limitations, including operational complexity, poor stereoselectivity, high‐temperature requirements, and economic inefficiency due to the extensive number of required chemical steps.

Several biocatalytic strategies have also been developed for the efficient production of *α*‐hydroxy ketones (Scheme 1B). Thiamine diphosphate (ThDP)‐dependent lyases catalyze the condensation of two carbonyl compounds to produce enantiopure *α*‐hydroxy ketones [12, 13]. Enzymes such as benzaldehyde lyase, benzoylformate decarboxylase, transketolases, CDP‐yersiniose biosynthesis protein YerE, and pyruvate decarboxylases play key roles in these reactions [14, 15]. Notably, the use of pyruvate decarboxylases for L‐phenylacetylcarbinol production has been successfully scaled up [16]. Another effective approach is the dynamic kinetic resolution (DKR) of racemates catalyzed by lipases, which has enabled the synthesis of a diverse range of enantiopure *α*‐hydroxy ketones, including aliphatic, alkyl‐aryl, and diaryl *α*‐hydroxy ketones [17, 18, 19]. Moreover, *α*‐hydroxylation of ketones represents a powerful synthetic strategy [20, 21]. For instance, cytochrome P450s (CYPs) and Fe(II)/*α*‐ketoglutarate‐dependent dioxygenases (*α*KGDs) have been engineered for regio‐ and stereospecific C–H hydroxylation to synthesize *α*‐hydroxy ketones [22, 23]. Despite these advances, further research is needed to broaden the substrate scope and optimize reaction conditions for these biocatalytic processes.

Oxidoreductases are also a powerful tool for producing enantioenriched *α*‐hydroxy ketones through the reduction of diketones or the oxidation of vicinal diols [24, 25, 26]. NAD(P)H‐dependent acetoin reductases/2,3‐butanediol dehydrogenases (EC 1.1.1.4 and 1.1.1.76) are promising oxidoreductases for the enantioselective synthesis of *α*‐hydroxy ketones [27, 28]. These enzymes catalyze the interconversion between diacetyl (DA), acetoin (AC), and 2,3‐butanediol (2,3‐BD) [29, 30]. 2,3‐BD contains two stereocenters and has three stereo isomers: *meso*‐, (2*S*,3*S*)‐, and (2*R*,3*R*)‐forms. Based on this, 2,3‐butanediol dehydrogenases (2,3‐BDHs) are classified into three types: *meso*‐BDH, (2*S*,3*S*)‐BDH, and (2*R*,3*R*)‐BDH [31]. Some 2,3‐BDHs are suitable for producing *α*‐hydroxy ketones by the reduction of diketones. For example, a *meso*‐BDH from *S. marcescens* CECT 977 exhibits remarkable substrate promiscuity. It efficiently catalyzes the asymmetric reduction from prochiral *α*‐diketones to corresponding *α*‐hydroxy ketones, including aliphatic cyclic and alkyl phenyl diketones [32]. Similarly, a (2*R*,3*R*)‐BDH from *B. clausii* DSM 8716T has been characterized for its ability to catalyze the asymmetric reduction of prochiral 1,2‐diketones, ranging from aliphatic diketones to alkyl phenyl dicarbonyl compounds. Conversely, some 2,3‐BDHs exhibit high oxidation activity toward vicinal diols [28]. For example, a (2*R*,3*R*)‐BDH from *Bacillus subtilis* has been used to oxidize 1,2‐cyclopentanediol, 1,2‐cyclohexanediol, 1,2‐cycloheptanediol, and 1,2‐cyclooctanediol. This enzyme produces the corresponding (*R*)‐*α*‐hydroxy ketones with high stereoselectivity and yield [33]. By coupling this enzyme with epoxide hydrolase (EH), a cascade biocatalysis system was developed, which effectively converted various epoxides into the corresponding *α*‐hydroxy ketones (Scheme 1C) [34].

Although 2,3‐BDHs have great potential for synthesizing *α*‐hydroxy ketones, they are limited by low substrate stereoselectivity. Since 2,3‐BD exists in three stereoisomers, these enzymes cannot achieve 100% stereoselectivity in distinguishing these isomers. For example, *meso*‐BDHs accept both *meso*‐2,3‐BD and (2*S*,3*S*)‐BD, with a stereoselectivity ratio of approximately 10:1, based on the activity ratio between *meso*‐2,3‐BD and (2*S*,3*S*)‐BD [35]. Similarly, (2*S*,3*S*)‐BDHs display a strong preference for (2*S*,3*S*)‐BD over *meso*‐2,3‐BD, with a more significant stereoselectivity ratio (approximately 132:1) [30]. (2*R*,3*R*)‐BDHs exhibit significantly lower stereoselectivity compared to (2*S*,3*S*)‐BDHs and *meso*‐BDHs. They accept (2*R*,3*R*)‐BD and *meso*‐2,3‐BD almost equally, with the stereoselectivity ratios of 1.4:1, 0.7:1, 2.3:1, and 0.78:1 for (2*R*,3*R*)‐BDHs from *Paenibacillus polymyxa* ATCC 12321 [36], *Neisseria gonorrhoeae* FA1090 [37], *Saccharomyces cerevisiae* YAL060W [38], and *Bacillus thuringiensis* [39], respectively.

Among characterized (2*R*,3*R*)‐BDH enzymes, (2*R*,3*R*)‐BDH from *Bacillus subtilis* (*Bs*BDH) stands out for its higher catalytic efficiency. Its X‐ray crystal structure (PDB ID: 6IE0) has been elucidated and displayed on the RCSB protein data bank [40]. *Bs*BDH has been used in cascade catalysis to produce intermediates, such as 2‐phenylglycinol, 2‐hydroxycyclohexanone (2‐HCH), and 2‐hydroxy‐1‐tetralone (Scheme 1C) [34]. However, *Bs*BDH exhibits similar activity toward (2*R*,3*R*)‐BD and *meso*‐BD, with a stereoselectivity ratio of 1.7:1. This low stereoselectivity limits its application in the synthesis of chiral *α*‐hydroxyketones, particularly when mixed stereoisomers are used as substrates.

Directed evolution is a powerful strategy for engineering enzymes with improved properties, such as thermostability, activity, and enantioselectivity [41]. However, screening a large‐sized mutation library is time‐consuming and labor‐intensive. To address this, targeted mutagenesis guided by structural or sequence information has become increasingly popular. This approach allows for the construction of smaller, more focused libraries, so‐called small‐but‐smart libraries [42, 43], that improve the efficiency of directed evolution by increasing the ratio of beneficial to detrimental mutations. Methods like iterative saturation mutagenesis (ISM) and CASTing typically require screening only moderate‐sized libraries, ranging from 5,000 to 20,000 mutants [44, 45]. However, the success of targeted mutagenesis depends heavily on identifying the right hot spots. This requires a thorough understanding of the protein structure‐function relationship. Software‐assisted approaches, such as those using Rosetta, have also been successful in redesigning enzymes to improve their functions [46, 47]. Nonetheless, computational predictions typically contain many false‐positive mutations, necessitating extensive experimental screening to identify true positives [48]. One common computational method involves estimating changes in free energy ∆∆G, to guide thermostability design. Over the past decade, tools like Rosetta_ddg [49], FoldX [50], ABACUS [51] have been widely used. While these tools can effectively filter out most true‐negative predictions, the false‐positive mutation rate remains high. For example, the experimental validation of PROSS—a server for predicting thermostable mutants—showed a true‐positive rate of only 35%, while the true‐negative rate was 99.6% [52]. This high false‐positive rate results in significant experimental effort.

In this study, we combined computational design, iterative saturation mutagenesis, and non‐canonical amino acid (ncAA) incorporation to enhance and invert the stereoselectivity of *Bs*BDH toward 1,2‐cyclohexanediol (1,2‐CHD). Using the engineered stereoselective *Bs*BDH variants, we aim to produce chiral *R*‐HCH or *S*‐HCH from mixed CHD stereoisomers. This can be readily synthesized via chemical catalysis, such as the hydrogenation of catechol (Scheme 1C) [53]. We first identified the key hot spots influencing the stereoselectivity through molecular docking and the protein language model ESM‐1v. Then, we developed a PASS (Position Analysis and Source Search) strategy to enhance the thermostability of *Bs*BDH, which provides a stable framework for engineering stereoselectivity. Subsequently, iterative saturation mutagenesis was performed on these hot spots, using the thermostable variant as template. This resulted in variants with excellent stereoselectivity and significantly increased activity. Many of these high‐activity variants also showed improved activity toward 25 substrates. Finally, molecular dynamics (MD) simulations and ncAA incorporation were used to elucidate the mechanisms behind these enhancements. Overall, our study demonstrates that combining protein language models for hot spots identification with iterative saturation mutagenesis enables rapid optimization of enzymatic activity and stereoselectivity.

## 2 Results and Discussion

### 2.1 Molecular Docking and Protein Language Model‐Guided Identification of Hot Spots

**FIGURE 1:**
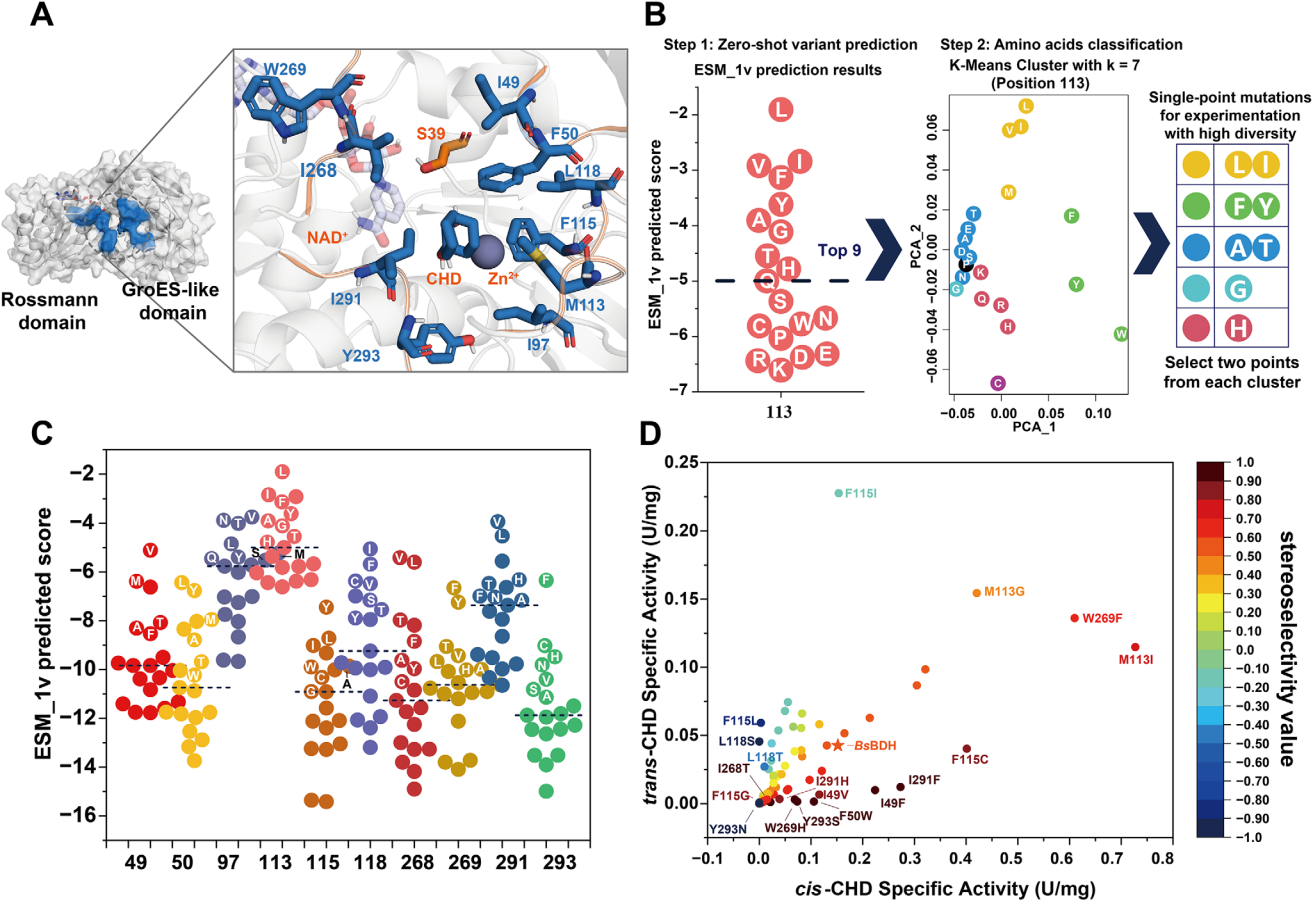
PLM‐guided prediction of single mutations. (A) Identification of key residues in the binding pocket. Five loops (orange) form a cleft (blue) that regulates substrate entrance and product release, thus shaping the binding pocket near the surface. Ten residues were identified as hot spots surrounding CHD. The structure of *Bs*BDH was obtained from the RCSB Protein Data Bank (PDB ID: 6IE0). (B) Schematic representation of zero‐shot prediction for potential single mutations at position 113 using the ESM‐1v model. (C) Zero‐shot prediction of potential single mutations in 10 hot spots. (D) Activity and stereoselectivity of single mutants of *Bs*BDH. Stereoselectivity value was calculated using the formula (SA*_cis_*‐SA*_trans_*)/(SA*_cis_* +SA*_trans_*), where SA*_cis_* and SA*_trans_* represent specific activities toward *cis*‐CHD and *trans*‐CHD, respectively. The single mutants showing the activity more than 3‐fold higher than that of *Bs*BDH, or the stereoselectivity value greater than 0.85 or less than −0.4 are labelled.

The complex structure of *Bs*BDH with the cofactor NAD^+^ was predicted by AlphaFold3. The structural environment surrounding NAD^+^ demonstrated a high degree of alignment with the structure of the alcohol dehydrogenase from *Sulfolobus solfataricus* (PDB ID: 1R37) [54], which is a homologous enzyme of *Bs*BDH (Figure S1A). The substrate *cis*‐CHD consists of the *meso*‐CHD configuration, while *trans*‐CHD is composed of two configurations: (1*S*,2*S*)‐CHD and (1*R*,2*R*)‐CHD (Scheme 1C). However, *Bs*BDH exhibits no catalytic activity toward (1*S*,2*S*)‐CHD. In order to identify the hot spots determining *Bs*BDH's stereoselectivity toward 1,2‐CHD, we subsequently docked *cis*‐CHD (*meso*‐CHD) and (1*R*,2*R*)‐CHD into the *Bs*BDH structure. *Bs*BDH comprises a *C*‐terminal Rossmann domain that binds the cofactor NAD^+^ or NADH, as well as a GroES‐like domain situated in the *N*‐terminal region [26]. Between the Rossmann and GroES‐like domains, there exists a cleft formed by several loops, which contains the substrate binding pocket and the Zn^2+^ binding site, including residues C37, H70, E71, and E152 (Figure S1B). Quantum mechanical calculations have been performed to investigate the oxidation mechanism of the MDR family, identifying S39 and H42 as key residues for catalysis (Figure S1C) [55]. Sequence alignment with MDRs from *Thermoanaerobacter brockii* [56] and *Rhodococcus erythropolis* WZ010 [57] revealed that S39 in *Bs*BDH was crucial for the final two steps of proton transfer (Figure S2). According to the oxidation mechanism, the substrates *cis*‐CHD (*meso*‐CHD) and (1*R*,2*R*)‐CHD were manually docked to ensure proper orientation at the reactive site. The hydroxyl groups at the *R*‐configured stereocenter of both substrates were held in similar positions. However, distinct differences were noted in their binding modes within the binding pocket. The six‐membered ring of *cis*‐CHD showed a preference for orientation toward residues 115 and 118, while (1*R*,2*R*)‐CHD favored to form hydrophobic interactions with residues 115 and 291 (Figure S1D). According to the proposed mechanism of the MDR family, the hydroxyl group at the *R*‐configured stereocenter of the substrate undergoes deprotonation via proton transfer through S39, 2’‐OH on the NAD^+^, and H42. Following this, the hydride then transfers from the donating carbon of the *R*‐configured stereocenter (C_D_) to the C‐4 (the accepting carbon C_A_) of the nicotinamide ring of NAD^+^ (Figure S1D). To confirm the reliability of the manual docking, we additionally employed molecular docking using Autodock Vina and selected reactive poses from the generated docking sets. The orientations of *cis*‐CHD (*meso*‐CHD) and (1*R*,2*R*)‐CHD within the substrate‐binding pocket aligned closely with those obtained from manual docking (Figure S3A). The docking results indicated that the binding pocket for the two substrates is primarily formed by five loops, including 47–52 aa, 96–100 aa, 113–120 aa, 265–270 aa, and 290–295 aa (Figure S3B; Figure 1A). These loops not only define the substrate binding pocket but also play a vital role in regulating both substrate entry and product release. Additionally, we then identified ten residues within 8 Å distance from the substrate CHD as hot spots, which include I49, F50, I97, M113, F115, L118, I268, W269, I291, and Y293 (Figure 1A).

Protein language models (PLMs), such as ESM models, were trained on billions of protein sequences spanning the evolutionary tree of life and learned the fundamental principles of protein structure and function [58]. The captured knowledge in PLMs can be utilized for zero‐shot predictions of advantageous mutations aimed at engineering proteins with improved stability, activity, and other desirable properties. Since the 10 identified hot spots are located within the binding pocket, mutations at these positions would significantly impact enzyme activity and even lead to the loss of activity. Hence, we employed the ESM model to predict promising single mutations, with the primary goal of excluding mutations that would significantly decrease enzyme activity. To efficiently explore the sequence space and minimize experimental cost, we implemented a two‐step screening process for the mutants predicted by the ESM‐1v model. First, the top 9 mutants with the highest prediction scores were identified at each mutation site. Subsequently, the 20 amino acids at each mutation site were clustered into 7 classes based on their ESM encodings. From each cluster, we selected up to 2 mutations ranked within the top 9 to ensure diversity among the constructed mutations. For example, at position 113, the top 9 mutations predicted by ESM‐1v included M113L, M113I, M113V, M113F, M113Y, M113A, M113G, M113T, and M113H. All 20 mutations at the site were clustered into 7 classifications. Based on the prediction scores of these mutations, we ultimately selected 8 mutations from 5 classifications for further characterization (Figure 1B). Following the same selection criteria, we identified 5, 6, 8, 8, 7, 7, 7, 7, 7 and 7 mutations for positions I49, F50, I97, M113, F115, L118, I268, W269, I291, and Y293, respectively, for a total of 69 single mutants for characterization (Figure 1C; Figure S4). To validate the effectiveness of the PLM in predicting active mutants, we also randomly selected 22 single mutants with low prediction scores for characterization as a control (Figure S5).

We constructed these single mutants and characterized their activities toward *cis*‐CHD (*meso*‐CHD) and (1*R*,2*R*)‐CHD, respectively. Since (1*R*,2*R*)‐CHD was not commercially available, we tested the activity toward *cis*‐CHD (*meso*‐CHD) and *trans*‐CHD, which comprises (1*S*,2*S*)‐CHD and (1*R*,2*R*)‐CHD, and calculated the stereoselectivity based on the activity data. In this study, the stereoselectivity value was defined as (SA*_cis_*‐SA*_trans_*)/(SA*_cis_* +SA*_trans_*), where SA*_cis_* and SA*_trans_* are the specific activities toward *cis*‐CHD and *trans*‐CHD, respectively. Among 69 mutants, 62 retained their enzymatic activity, while F50T, I97N, I268V, Y293F, Y293C, Y293V, and Y293A showed complete loss of activity (Table S1). In contrast, 15 of 22 single mutants with low prediction scores showed a complete loss of activity (Figure S5). This indicated that the ESM‐1v model was effective in predicting high‐fitness mutants while successfully excluding those with significantly decreased activities. Among 62 active mutants, 22 variants showed improved preference for *cis*‐CHD. The activity of *Bs*BDH toward *cis*‐CHD (0.15 U/mg) was 3.56‐fold higher than that toward *trans*‐BDH (0.043 U/mg), leading to a stereoselectivity value of 0.561. Notably, the top 3 mutants F50W, W269H, and Y293S exhibited stereoselectivity values of 0.973, 0.960, and 0.919, respectively, significantly exceeding that of *Bs*BDH (Table S1; Figure 1D). However, F50W, W269H, and Y293S showed decreased activity toward *cis*‐CHD, with their respective activities being 1.36, 2.02, and 2.17‐fold lower than that of *Bs*BDH.

Interestingly, 11 single mutants demonstrated reversal of the substrate stereoselectivity, switching their preference from *cis*‐CHD to *trans*‐CHD. The top 3 mutants, L118S, Y293N, and F115L, exhibited stereoselectivity values of −1, −1, and −0.897, respectively. However, these mutants showed low activity toward *trans*‐CDH, with values of 0.046, 0.00067, and 0.059 U/mg, respectively (Table S1; Figure 1D). Furthermore, several mutants were identified that exhibited significantly improved activity toward both substrates. For example, compared to *Bs*BDH, W269F showed a 4‐fold improvment in activity toward *cis*‐CHD, and a 3.26‐fold increase in activity toward *trans*‐CHD, while M113I improved activity by 4.85‐fold toward *cis*‐CHD, and by 2.67‐fold toward *trans*‐CHD (Figure 1D). Notably, F115I achieved the highest activity (0.23 U/mg) toward *trans*‐CHD, which was 5.3‐fold higher than *Bs*BDH.

Based on the results, 9 of the 10 identified hot spots, excluding I97, yielded mutations that either enhanced or inverted stereoselectivity. Among these, residues 115, 118, and 293 had the most significant impact on stereoselectivity. Y293N, F115L/I and L118S/T/V inverted the stereoselectivity from *cis*‐CHD preference to *trans*‐CHD preference, while F115W/C/G, Y293S markedly improved *cis*‐CHD preference. The improvements in stereoselectivity were closely related to alterations in the shape and polarity of the binding pocket. To further investigate the effect of the binding pocket shape on the CHD stereoselectivity and activity, we focused on exploring the combinatorial sequence space of mutations at positions 115, 118, and 293. However, since *Bs*BDH exhibited limited thermostability, with a half‐life *t*_1/2_ of only 36.16 min at 37°C (Figure S6), we first enhanced the thermostability of *Bs*BDH before exploring the combinatorial fitness landscape, as the wild‐type *Bs*BDH might not be able to tolerate the introduction of a significant number of mutations.

### 2.2 Computational Design to Improve Thermostability of *Bs*BDH

**FIGURE 2:**
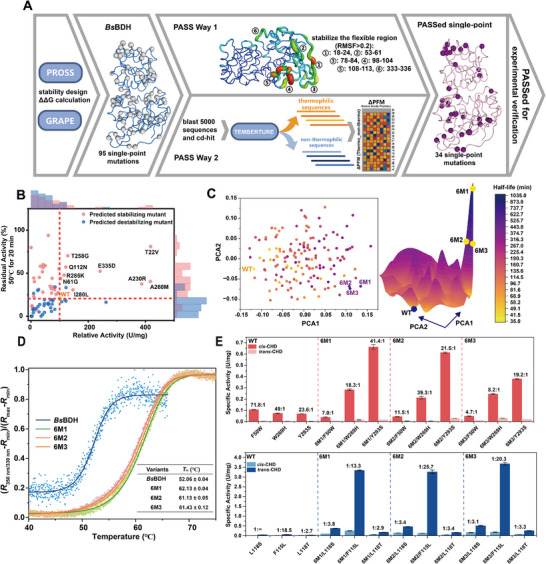
Computational design to improve the thermostability of *Bs*BDH. (A) Schematic diagram of the PASS strategy. First, single mutations were generated with the GRAPE and PROSS strategies. Next, mutations were filtered by two ways, which included PASS Way 1 based on flexibility analysis and PASS Way 2 based on protein evolutionary information. (B) Experimental verification for predicted stabilizing and destabilizing mutants. 88.2% of predicted stabilizing mutants were true‐positive, and 88.2% of predicted destabilizing mutants were true‐negative. (C) Fitness landscape of thermostability. The thermostability was shown with the half‐life time at 37°C. 6M1, 6M2, 6M3 showed the highest thermostability. (D) Unfolding curves and *T*_m_ values of the wild‐type and its mutants. (E) Activities and stereoselectivities of *Bs*BDH mutants combined with thermostable mutations. Red indicates the mutants with improved stereoselectivity for *cis*‐CHD, while blue indicates the mutants with inverted stereoselectivity. The data were presented as mean values ± SD from three independent biological replicates (n = 3). The error bars represent the standard deviation (SD).

To engineer *Bs*BDH for improved thermostability, we developed a PASS (Position Analysis and Source Search) strategy that considers both the position and the source of mutations to reduce the false‐positive rate of the mutations predicted by ∆∆G calculation (Figure 2A). The strategy includes two PASS ways for filtering mutations predicted by ∆∆G calculations, and only those that pass the filtration proceed to experimental verification. The PASS way 1 considers the position of mutations, and only those located in flexible regions are selected for characterization. Rigidifying flexible sites has been reported to be a useful strategy to improve protein stability [59]. Therefore, the single mutations located at the flexible loops are considered to be beneficial for enhancing thermostability. The PASS way 2 involves the analysis of evolutionary information related to protein thermostability to identify mutants present in thermophilic homologous while filtering out those derived from non‐thermophilic homologous proteins. Here, we applied the deep learning model TemBERTure to classify protein sequences into thermophilic or non‐thermophilic classes [60]. Mutations exhibiting higher frequencies in the thermophilic sequence position matrix are prioritized for subsequent experimental verification.

*Bs*BDH was then designed by the PASS strategy to improve thermostability. First, 95 single mutations were predicted to be more stable based on ∆∆G calculations using PROSS and GRAPE strategies, with only D132P being predicted by both strategies (Table S2). To eliminate the false‐positive mutants through PASS way 1, we carried out a 100 ns molecular dynamics (MD) simulation of *Bs*BDH, which revealed six flexible regions characterized by root‐mean‐square‐fluctuation (RMSF) values exceeding 0.2 nm, including 18–24 aa, 53–61 aa, 78–84 aa, 98–104 aa, 108–113 aa, and 333–336 aa (Figure S7). Six mutations including K21Q, T22V, N61G, E84T, Q112N, and E335D were located in these flexible regions, and selected for further characterization.

To apply PASS way 2 for filtering the mutations, we obtained 5000 homologous sequences from the Non‐Redundant Protein Sequence Database (NR) via blastp using the sequence of *Bs*BDH as the query sequence, with similarities ranging from 40% to 100%. After removing highly similar or repeated sequences using the cd‐hit cleaning [61], a protein sequence library consisting of 691 sequences with pairwise similarity lower than 90% was obtained for TemBERTure prediction (Figure S8).

In the results, 272 sequences were predicted to be thermophilic, and 419 sequences were predicted to be non‐thermophilic. The thermophilic and non‐thermophilic sequences were then aligned separately to calculate the position frequency matrix (PFM), which quantifies the frequencies of each amino acid at each position based on the alignment. ∆PFM was then calculated by subtracting PFM(thermophilic) from PFM(non‐thermophilic) for all 80 mutation sites where the 95 single mutations with favorable ∆∆G values are located (Figure S9). ∆PFM values clearly revealed the preference of certain amino acids for thermophilic sequences or non‐thermophilic sequences. For example, position 84 showed a preference to have the polar amino acid T over the acidic amino acid E (Figure S9). Interestingly, analysis of ∆PFM values also revealed that similar types of amino acids could have different preferences. For example, in thermophilic sequences, position 86 favored F over L, position 285 favored K over R, and 280 favored L over I. We attempted to select single mutations with ∆∆G < 0 and ∆PMF > 0, which identified the mutations not only energetically stabilizing the protein but also aligning with amino acid preferences observed in nature, particularly in thermophilic sequences. After the PASS Way 2 filtration, 28 single mutations were selected, which include W6L, T67I, M68L, Y86F, L118F, S130L, D132P, F137H, E142N, Y145F, S154M, Y159H, Q206A, D223E, A230R, T258G, I259K, A260M, T263I, H276N, I280L, K283Y, R285K, P298E, A299E, L301I, K305S, and V320K (Figure S9).

Due to the low activity of *Bs*BDH toward the substrate CHD, we used the natural substrate (2*R*,3*R*)‐butanediol to assay the thermostability of 34 single mutants obtained after filtration by two PASS ways. The residual activity was tested for WT and all mutants in cell lysates after being heated at 50°C for 20 min. Thirty mutants showed improved thermostability, while only 4 mutants exhibited decreased thermostability, including S154M, K283Y, P298E, and A299E (Figure 2B). The most stable mutant I259K showed a residual activity of 94.0%, which was 4.5‐fold higher than that of WT (20.7%). In addition, 9 mutants exhibited both improved activity and thermostability, with T22V, N61G, Q112N, and E335D being selected by PASS Way 1, while A230R, T258G, A260M, I280L, and R285K were identified through PASS Way 2. To confirm this, we purified these 9 single mutants and measured their initial activities and residual activities after heat treatment at 37°C for 90 min (Figure S10). All the mutants enhanced residual activity and maintained comparable or higher activity than *Bs*BDH. Q112N exhibited the highest activity with a 3.8‐fold improvement and a 2.6‐fold improvement in residual activity. A260M showed the highest residual activity of 67.2%, which was 3.01‐fold higher than WT, along with a 1.6‐fold improved activity.

Thirty of the 34 mutants predicted by the PASS strategy exhibited higher thermostability, demonstrating a true‐positive rate of 88.2%, significantly higher than traditional methods such as PROSS, which had a true‐positive rate of only 35%. To confirm that the predicted destabilizing mutants were true‐negative, we selected an equivalent number of 34 negative mutants with ∆PFM < 0 for experimental verification (Table S3). The results indicated that 30 of these mutants showed lower thermostability than *Bs*BDH (Figure 2B), including Y86L with a residual activity of 19.5% and H276D with a residual activity of 13.9%, leading to a true‐negative rate of 88.2%. This clearly demonstrates the high accuracy of the PASS strategy in predicting true‐negative destabilizing mutants. The false predicted mutants, including N8G, S202I, E242D, and Q338I exhibited a weakly improved thermostability, and N8G was the most stable with residual activity of 33.9%, which was only a 1.6‐fold improvement compared to *Bs*BDH (Table S3).

In order to obtain mutants with further improved thermostability, we used Gibson assembly to randomly combine nine single mutations showing both improved thermostability and activity. The mutations were divided into three gene fragments based on their positions, with 1–514 bp covering T22V/N61G/Q112N, 494–808 bp covering A230R/T258G/A260M, and 788‐1041 bp covering I280L/R285K/E335D, resulting in a combinatorial library containing 8 × 8 × 8 = 512 mutations. We randomly selected and tested 142 combinatorial mutants for half‐life time at 37°C, including 29 double mutants, 18 triple mutants, 38 quadruple mutants, 30 quintuple mutants, 16 sextuple mutants, 8 septuple mutants, 2 octuple mutants, and 1 nonuple mutant (Table S4). In the results, all these combinatorial mutants exhibited higher thermostability than WT, and the double mutant Q112N/R285K was the worst one, showing a half‐life time of 45.3 min, which was 1.3‐fold higher than WT. We constructed the fitness landscape composed of 152 verified mutants, including WT, 9 single, and 142 combinatorial mutants, revealing that the fitness landscape was highly rugged (Figure 2C). The top three mutants T22V/N61G/Q112N/A230R/A260M/I280L (6M1), T22V/Q112N/A230R/T258G/A260M/I280L (6M2) and T22V/N61G/A230R/T258G/A260M/I280L (6M3) displayed half‐life time of 1033.8, 533.1 and 495.1 min, which was 28.5‐fold, 14.8‐fold and 13.7‐fold higher than 36.1 min of *Bs*BDH, respectively (Table S4; Figure 2C). These three mutants showed a similar specific activity of 0.5 U/mg tested with cell lysates, around 3‐fold higher than *Bs*BDH. However, after purification, 6M2 showed the highest activity of 0.91 U/mg, slightly higher than 0.73 U/mg of 6M1 and 0.80 U/mg of 6M3, and significantly higher than 0.51 U/mg of *Bs*BDH (Figure S11). It was also found that 6M1, 6M2, and 6M3 significantly improved the melting temperature *T*_m_ of the enzyme by 10.1°C, 9.1°C and 9.4°C, respectively (Figure 2D).

We then combined 6M1, 6M2, and 6M3 with mutations F50W, W269H, Y293S that improved the stereoselectivity, and L118S, F115L, L118T that inverted the stereoselectivity (Figure 2E). The thermostable mutants generally enhanced the crude enzyme activities of these single mutants. For example, 6M1 improved the F115L activity by 78.2‐fold toward *cis*‐CHD, and by 56.3‐fold toward *trans*‐CHD, respectively. The improvement in crude enzyme activities may be attributed to the increased soluble expression of the mutants.

However, since the thermostable mutants did not equally improve the activity of single mutants toward *cis*‐CHD and *trans*‐CHD, the stereoselectivity was different for the stereoselective variants before and after combined with the thermostable mutants. The incorporation of thermostable variants generally decreased the stereoselectivity of stereoselective mutations. For example, F50W showed a *cis*:*trans* selectivity ratio of 71.8:1, which was decreased to 7.0:1, 11.5:1 and 4.7:1 of 6M1/F50W, 6M2/F50W and 6M3/F50W, respectively. However, several combinational variants still maintain high stereoselectivity. 6M1/Y293S and 6M2/W269H were the top two mutants showing improved stereoselectivity for *cis*‐CHD, with the *cis*:*trans* selectivity ratio of 41.4:1 and 39.3:1, similar with those of Y293S and W269H. Additionally, 6M2/F115L exhibited the highest *trans*:*cis* selectivity ratio of 25.7:1, higher than 18.5:1, 13.3:1 and 20.3:1 of F115L, 6M1/F115L and 6M3/F115L, respectively (Figure 2E). We also measured the kinetic parameters of WT and 6M2. The catalytic efficiencies of 6M2 toward substrates *cis*‐CHD and *trans*‐CHD were determined to be 0.268 ± 0.029 s^−1^mM^−1^ and 0.023 ± 0.003 s^−1^mm ^−1^, which were 5.92‐fold and 2.15‐fold higher than WT, respectively (Figure S12; Table 1). Considering the performance of 6M2 in maintaining the stereoselectivity of single mutants and its high activity, we chose 6M2 as the starting sequence for the subsequent protein engineering process.

**TABLE 1:** Kinetic parameters of *Bs*BDH variants.

mutants	substrate	*k*_cat_ (s^−1^)	*K*_m_ (mM)	*k*_cat_/*K*_m_ (s^−1^mm ^−1^)	fold
WT	*cis*‐CHD	1.59 ± 0.09	35.06 ± 3.37	0.045 ± 0.006	1
WT	*trans*‐CHD	0.76 ± 0.06	69.80 ± 6.25	0.011 ± 0.002	1
6M2	*cis*‐CHD	3.33 ± 0.16	12.44 ± 0.98	0.268 ± 0.029	5.92
6M2	*trans*‐CHD	0.85 ± 0.05	36.35 ± 2.67	0.023 ± 0.003	2.15
6M2/F115C/L118F	*cis*‐CHD	22.15 ± 1.84	5.21 ± 0.54	4.255 ± 0.664	94.10
6M2/F115L/L118M	*trans*‐CHD	18.99 ± 0.31	5.50 ± 0.28	3.456 ± 0.220	317.85
10M	*cis*‐CHD	0.92 ± 0.01	22.41 ± 0.78	0.041 ± 0.001	0.90
LTF	*trans*‐CHD	1.18 ± 0.04	71.63 ± 7.58	0.016 ± 0.002	1.51
NC‐WT	*cis*‐CHD	0.14 ± 0.007	30.47 ± 3.17	0.0047 ± 0.001	1
NC‐6M2/Y293S/L118F	*cis*‐CHD	0.78 ± 0.08	5.41 ± 0.88	0.145 ± 0.033	30.52
NC‐6M2/Y293S/L118F/F50‐2,5‐2ClF	*cis*‐CHD	6.87 ± 0.04	6.74 ± 0.40	1.020 ± 0.072	214.48

### 2.3 Perfect Iterative Saturation Mutagenesis to Engineer Stereoselectivity of *Bs*BDH

**FIGURE 3:**
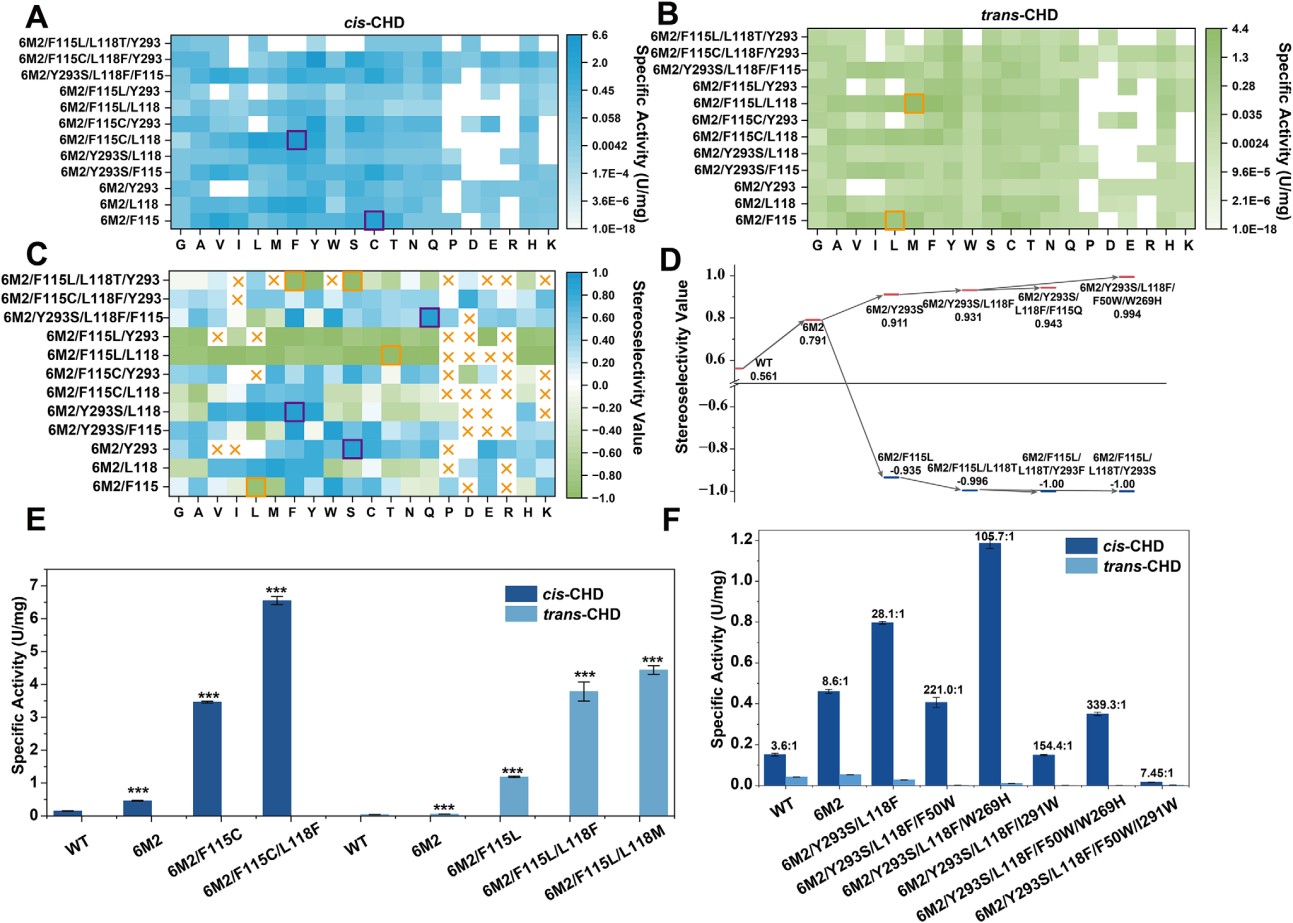
Iterative saturation mutagenesis to engineer *Bs*BDH. (A) Activities of the mutants at residues F115, L118, and Y293 toward *cis*‐CHD. (B) Activities of the mutants at residues F115, L118, and Y293 toward *trans*‐CHD. (C) Stereoselectivity of the mutants at residues F115, L118, and Y293. (D) Evolutionary paths to increase and invert the stereoselectivity of *Bs*BDH. (E) High activity mutants toward *cis*‐CHD and *trans*‐CHD. The asterisks show statistically significant differences from WT. (F) Evolutionary paths to increase the stereoselectivity of *Bs*BDH at residues F50, W269, and I291. The data were presented as mean values ± SD from three independent biological replicates (n = 3). The error bars represent the standard deviation (SD). Statistical significance was evaluated using one‐way analysis of variance (ANOVA). The asterisks of ^*^, ^**^, ^***^ denote *p* < 0.05, *p* < 0.01 and *p* < 0.001, respectively.

To further improve and invert the stereoselectivity of *Bs*BDH, we used 6M2 as the parent and constructed combinatorial mutants at F115, L118, and Y293. To efficiently explore the sequence space, we implemented a perfect iterative saturation mutagenesis (PISM) strategy. Different from traditional ISM that performs randomization at target sites by primers containing NNK, we constructed the saturation mutants at positions 115, 118, and 293 by site‐directed mutagenesis and characterized all mutants with the utility of iBioFoundry [62], an automatic facility to perform construction and characterization of protein mutants. In the first round of PISM, 57 single mutants (19^*^3) were constructed at positions 115, 118, and 293. We then tested their activity toward both *cis*‐CHD and *trans*‐CHD (Figure 3A,B), and calculated the stereoselectivity value (Figure 3C). It was interesting to find that 6M2/Y293S*_cis_* was still the best mutant to accept *cis*‐CHD, while 6M2/F115L*_trans_* was the best mutant to accept (1*R*,2*R*)‐CHD (Figure 3C,D). We also found 6M2/F115C showed the highest activity of 3.46 U/mg toward *cis*‐CHD, representing 23.1‐fold and 7.5‐fold increases compared to 0.15 U/mg of WT and 0.46 U/mg of 6M2, respectively, although its *cis*:*trans* selectivity ratio was only 4.9:1 (Figure 3A,C). This substitution from a non‐polar side chain to a polar side chain, resulting in increased activity, also indicated the strong plasticity of the binding pocket.

We then selected these three mutants, 6M2/Y293S*_cis_*, 6M2/F115L*_trans_* and 6M2/F115C as templates for subsequent construction of a total of 114 single mutants (19^*^2^*^3) at positions 115, 118 for 6M2/Y293S*_cis_*, and positions 118, 293 for 6M2/F115L*_trans_* and 6M2/F115C (Figure 3A,B). Among 38 mutants constructed based on 6M2/Y293S*_cis_*, 6M2/Y293S/L118F*_cis_* showed the highest stereoselectivity for *cis*‐CHD, with a stereoselectivity value of 0.931 and a *cis*:*trans* selectivity ratio of 28.1:1, higher than 21.5:1 of 6M2/Y293S*_cis_* (Figure 3C,D). The variant with the highest preference for *trans*‐CHD was 6M2/F115L/L118T*_trans_* with a stereoselectivity value of −0.996 and a *trans*:*cis* selectivity ratio of 476:1, significantly higher than 25.7:1 of 6M2/F115L*_trans_*, although its activity decreased to 0.47 U/mg, 2.5‐fold lower than 1.18 U/mg of 6M2/F115L*_trans_* (Figure 3C,D). Interestingly, 6M2/F115L/L118M*_trans_* improved both the stereoselectivity and activity compared to 6M2/F115L*_trans_*, with a stereoselectivity value of −0.955 and a specific activity of 4.44 U/mg (Figure 3C,E). 6M2/F115L/L118F*_trans_* also exhibited a specific activity of 3.78 U/mg toward *trans*‐CHD, higher than 1.18 U/mg of 6M2/F115L*_trans_*, but showed a stereoselectivity value of −0.806, lower than −0.935 of 6M2/F115L*_trans_* (Figure 3C,E).

The 38 mutants constructed based on 6M2/F115C showed different stereoselectivity, ranging from −0.8 of 6M2/F115C/L118A to 0.79 of 6M2/F115C/Y293S (Figure 3C). Although no mutants with improved stereoselectivity were obtained compared to 6M2/Y293S/L118F*_cis_* and 6M2/F115L/L118T*_trans_*, 6M2/F115C/L118F was obtained with the highest activity of 6.55 U/mg toward *cis*‐CHD, which was 1.9‐fold higher than 6M2/F115C (Figure 3C,E). Kinetic parameters were then determined for 6M2/F115C/L118F and 6M2/F115L/L118M*_trans_*. The catalytic efficiency of 6M2/F115C/L118F for *cis*‐CHD was measured to be 4.26 s^−1^mM^−1^, which was 94.1‐fold higher compared to WT. The catalytic efficiency of 6M2/F115L/L118M*_trans_* toward *trans*‐CHD was 3.46 s^−1^mM^−1^, which was 317.85‐fold higher than WT. Both 6M2/F115C/L118F and 6M2/F115L/L118M*_trans_* exhibited significant increases in *k*_cat_ and notable reductions in *K*_m_, indicating that the reshaped pockets in these two mutants are more favorable for substrate binding and conversion (Figure S12; Table 1).

In the third of round of PISM, 57 (19^*^3) mutations were constructed at position 115 of 6M2/Y293S/L118F*_cis_* to further improve stereoselectivity for *cis*‐CHD, at position 293 of 6M2/F115L/L118T*_trans_* to further improve stereoselectivity for *trans*‐CHD, and at position 293 of 6M2/F115C/L118F to further improve activity, respectively. We obtained a mutant 6M2/Y293S/L118F/F115Q*_cis_* showing an improved stereoselectivity with a value of 0.943 and a *cis*:*trans* selectivity ratio of 34.1:1, although its activity was decreased to 0.21 U/mg, even lower than 0.46 U/mg of 6M2. Additionally, two mutants 6M2/F115L/L118T/Y293S*_trans_* (LTS) and 6M2/F115L/L118T/Y293F*_trans_* (LTF) showed perfect stereoselectivity for *trans*‐CHD, with a stereoselectivity value of −1, whereas their activities were 0.13 and 0.018 U/mg, respectively. Both values were lower than 0.47 U/mg of 6M2/F115L/L118T*_trans_* (Figure 3B,D). Despite this, LTF was also found to show a *k*_cat_ of 1.18 s^−1^ toward *trans*‐CHD, which was 1.6‐fold higher than that of WT, while the mutant maintained a similar *K*_m_ value with WT, which leads to an improved catalytic efficiency of 0.016 s^−1^mM^−1^ (Table 1). However, among the mutants constructed based on 6M2/F115C/L118F, no further improved mutants were obtained with higher activity and stereoselectivity than 6M2/F115C/L118F and 6M2/Y293S/L118F/F115Q*_cis_*, respectively.

To further improve *cis*‐CHD stereoselectivity of *Bs*BDH, positions F50, W269, and I291 were considered for constructing combinatorial mutants using 6M2/Y293S/L118F*_cis_* as a template. These positions not only impact the shape of the substrate binding pocket but also the product release tunnel. 6M2/Y293S/L118F*_cis_* was further engineered by incorporating F50W, I291F, I291W, and W269H, which demonstrated high stereoselectivity for *cis*‐CHD (Figure 1D). We did not select 6M2/Y293S/L118F/F115Q*_cis_* as a template because it only showed weakly improved stereoselectivity but significantly lower activity compared to 6M2/Y293S/L118F*_cis_*. The variants with further improved stereoselectivity were obtained, including 6M2/Y293S/L188F/F50W, 6M2/Y293S/L188F/I291W and 6M2/Y193S/L118F/F50W/W269H (10M) showing a *cis*:*trans* selectivity ratio value of 221.0:1, 154.4:1 and 339.3:1, respectively, which were significantly higher than 28.1:1 of 6M2/Y293S/L118F*_cis_* (Figure 3F). Kinetics measurement revealed that the 10M variant exhibited a catalytic efficiency of 0.041 s^−1^mm ^−1^, similar to 0.045 s^−1^mm ^−1^ of WT toward *cis*‐CHD (Table 1). Further combination of 6M2/Y293S/L118F/F50W/W269H (10M) with either I291F or I291W led to significant reductions in both activity and stereoselectivity, probably due to steric hindrance between residues 50 and 291 that disrupted the substrate binding pocket.

### 2.4 Conversion of 1,2‐Cyclohexanediol by Whole‐Cell Catalysts

**FIGURE 4:**
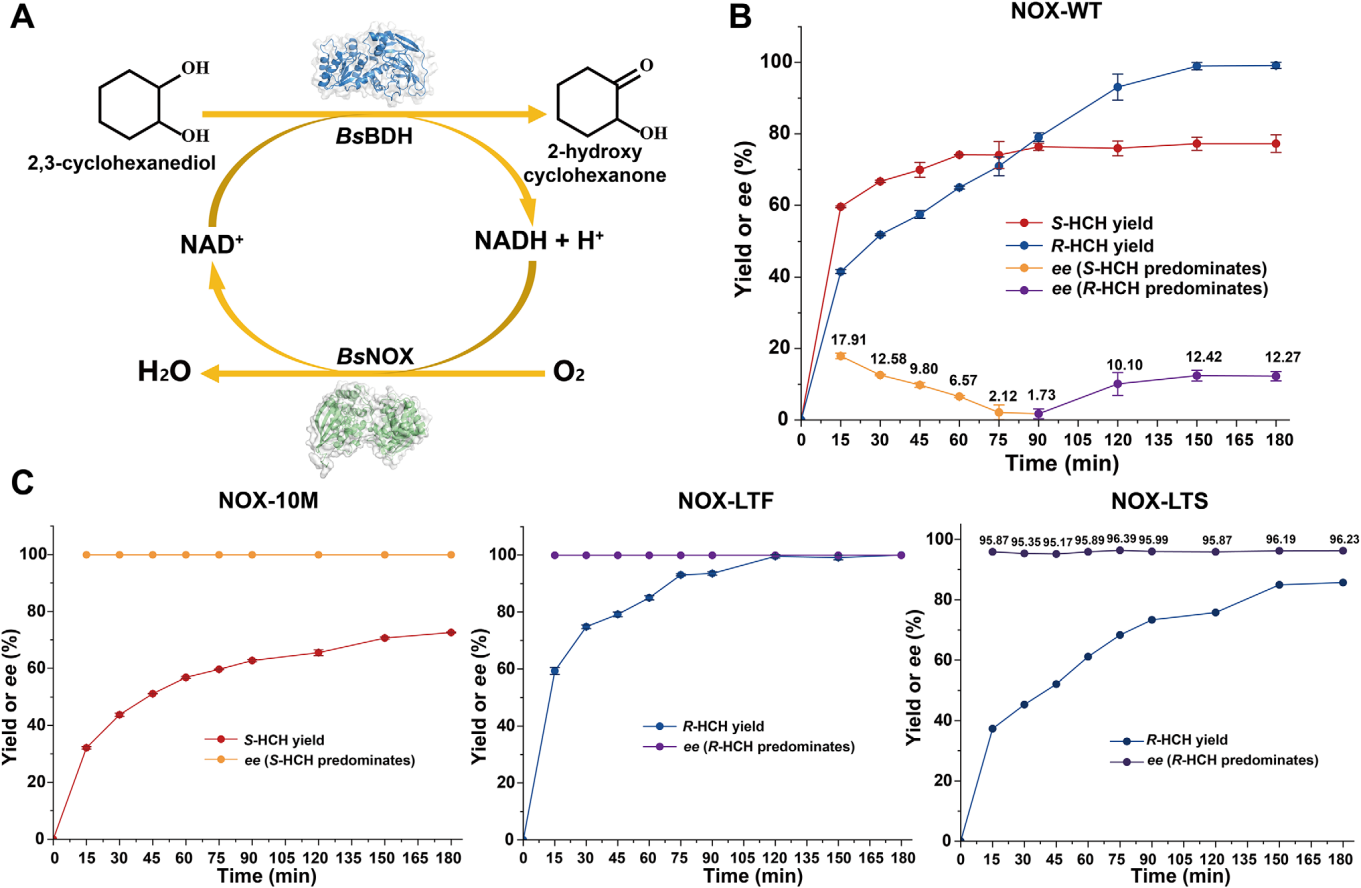
Whole‐cell catalytic reactions for synthesizing chiral 2‐hydroxycyclohexanone using wild‐type and mutant *Bs*BDH. (A) A recombinant *E.coli* whole‐cell biocatalyst with *Bs*BDH and the enzymatic NAD^+^ regeneration system for the production of chiral HCH. (B) Whole‐cell catalytic reactions for wild‐type *Bs*BDH. (C) Whole‐cell catalytic reactions for *Bs*BDH mutants. Reaction conditions were 10 mm CHD, OD_600_ of 5 for NOX‐WT, NOX‐10M, NOX‐LTS, and OD_600_ 30 for NOX‐LTF in Tris‐HCl buffer (pH 8.0) at 30°C and 220 rpm within 180 min. The data were presented as mean values ± SD from three independent biological replicates (n = 3). The error bars represent the standard deviation (SD).

Through the above target mutagenesis, we obtained a *cis*‐CHD preference mutant 6M2/Y293S/L118F/F50W/W269H*_cis_* (10M) and two *trans*‐CHD preference mutants 6M2/F115L/L118T/Y293S*_trans_* (LTS) and 6M2/F115L/L118T/Y293F*_trans_* (LTF). These three mutants were then evaluated for synthesizing chiral HCH using mixed CHD as substrate, which contained three stereoisomers with equimolar proportions. A NADH oxidase (NOX) from *Bacillus subtilis* was employed as the cofactor‐regeneration enzyme and co‐expressed with *Bs*BDH (Figure 4A). The whole cells expressing *Bs*BDH and NOX were used for converting 10 mM mixed CHD at 30°C for 180 min. Neither *Bs*BDH nor the mutants exhibited activity toward (1*S*,2*S*)‐CHD (Figure S13). Due to the low stereoselectivity of *Bs*BDH, NOX‐WT converted both *cis*‐CHD and (1*R*,2*R*)‐CHD, leading to a yield of 77.3% of *S*‐HCH and 99.1% of *R*‐HCH after 180 min reaction (Figure 4B; Figures S14 and S15). By contrast, 10M and LTF exhibited high stereoselectivity. NOX‐10M only oxidized *cis*‐CHD to *S*‐HCH, achieving a yield of 72.6% and an *ee* value of >99% after 180 min reaction (Figure 4C; Figures S14 and S15). Additionally, NOX‐LTF only converted (1*R*,2*R*)‐CHD to *R*‐HCH, while no *S*‐HCH was detected, achieving a yield of >99% and an *ee* value of >99% after 120 min. LTS was also an excellent (1*R*,2*R*)‐CHD stereospecific mutant, showing an *ee* value of 95.9% and a yield of 85.7% (Figure 4C).

### 2.5 Substrate Scope of High‐Activity *Bs*BDH Mutants

**FIGURE 5:**
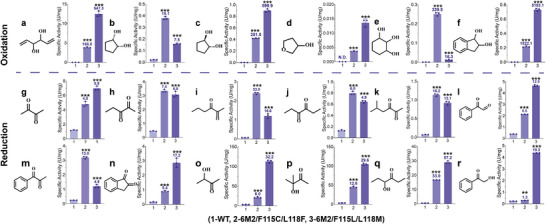
Substrate scope of the mutants 6M2/F115C/L118F and 6M2/F115L/L118M. The oxidation activity toward diols and the reduction activity toward *α*‐hydroxy ketones and diketones were measured. The fold increases in activity compared to WT are displayed above the columns. The asterisks show statistically significant differences from WT. (a) 1,5‐hexadiene‐3,4‐diol; (b) *cis*‐1,2‐cyclopentanediol; (c) *trans*‐1,2‐cyclopentanediol; (d) 3‐hydroxytetrahydrofuran; (e) 1,2,3‐cyclohexanetriol; (f) 1,2‐indandiol; (g) butanedione; (h) 2,3‐pentanedione; (i) 2,3‐hexanedione; (j) 3,4‐hexanedione; (k) 5‐methyl‐2,3‐hexanedione; (l) phenylglyoxal; (m) 1‐phenyl‐1,2‐propanedione; (n) 1,2‐indanedione; (o) acetoin; (p) 3‐hydroxy‐3‐methyl‐2‐butanone; (q) 4‐hydroxy‐3‐hexanone; (r) *α*‐hydroxyacetophenone. The data were presented as mean values ± SD from three independent biological replicates (n = 3). The error bars represent the standard deviation (SD). Statistical significance was evaluated using one‐way analysis of variance (ANOVA). The asterisks of ^*^, ^**^, ^***^ denote *p* < 0.05, *p* < 0.01 and *p* < 0.001, respectively.

During the evolution of *Bs*BDH, 6M2/F115C/L118F and 6M2/F115L/L118M exhibited significantly high activities with catalytic efficiency *k*_cat_/*K*_m_ being 94.1‐fold and 317.8‐fold higher than WT toward *cis*‐CHD and *trans*‐CHD, respectively (Table 1). This makes us wonder if these two variants could enhance the activity toward other substrates. We then tested these two mutants along with WT for oxidation of 9 substrates and reduction of 16 substrates, 25 substrates in total (Figure 5; Figure S16). Since some of the chiral compounds, such as 1,5‐hexadiene‐3,4‐diol (a), 1,2,3‐cyclohexanetriol (e), and 1,2‐indandiol (f) were not available, the racemic substrates were used for reactions instead. In oxidation reactions, the two mutants exhibited significantly higher activity toward the substrates compared to WT, particularly for the oxidation of cyclic substrates. For example, 6M2/F115C/L118F improved the enzyme activity by 168.0‐fold, 18.1‐fold, 281.0‐fold, 339.5‐fold and 1522.1‐fold toward 1,5‐hexadiene‐3,4‐diol (a), *cis*‐1,2‐cyclopentanediol (b), *trans*‐1,2‐cyclopentanediol (c), 1,2,3‐cyclohexanetriol (e) and 1,2‐indandiol (f), respectively. Notably, while WT showed no activity toward 2‐methoxycyclohexanol and 3‐hydroxytetrahydrofuran (d), 6M2/F115C/L118F exhibited a weak but measurable activity (Figure 5; Figure S16). Although 6M2/F115L/L118M also improved activity toward these substrates, the extent of improvement was different from that of 6M2/F115C/L118F, with the most significant improvement of 5183.1‐fold toward 1,2‐indandiol (f). The substrate scope encompassed cyclic compounds and aliphatic compounds, demonstrating their great potential for oxidizing various diols substrates to produce *α*‐hydroxy ketones in industrial applications (Figure 5; Figure S16).

Additionally, both 6M2/F115C/L118F and 6M2/F115L/L118M exhibited improved reduction activities toward 6 *α*‐hydroxy ketones and 10 diketones substrates. 2,3‐butanedione (g) and acetoin (o) are native substrates of *Bs*BDH. 6M2/F115L/L118M showed improved activity by 5.9‐fold and 32.2‐fold toward 2,3‐butanedione and acetoin, respectively. The two mutants also improved the reduction activity toward bulky substrates such as phenylglyoxal (l), 1‐phenyl‐1,2‐propanedione (m), 1,2‐indanedione (n), and *α*‐hydroxyacetophenone (r). The two mutants showed different substrate preferences toward *α*‐hydroxy ketones and diketones. For example, 6M2/F115C/L118F showed higher activity toward aliphatic vicinal diketones than 6M2/F115L/L118M, such as 2,3‐hexanedione (i), 3,4‐hexanedione (j), 5‐methyl‐2,3‐hexanedione (k), whereas 6M2/F115L/L118M exhibited higher activity toward bulky vicinal diketones and *α*‐hydroxy ketones, such as phenylglyoxal (l), 1,2‐indanedione (n), acetoin (o), 3‐hydroxy‐3‐methyl‐2‐butanone (p), 4‐hydroxy‐3‐hexanone (q), *α*‐hydroxyacetophenone (r). While the activity improvement for reduction was typically weaker than for oxidation, the expanded substrate scope of 6M2/F115C/L118F and 6M2/F115L/L118M showed their potential for improving the biocatalytic production of *α*‐hydroxy ketones and vicinal diols.

### 2.6 MD Simulations to Reveal the Mechanism of Improved Variants

**FIGURE 6:**
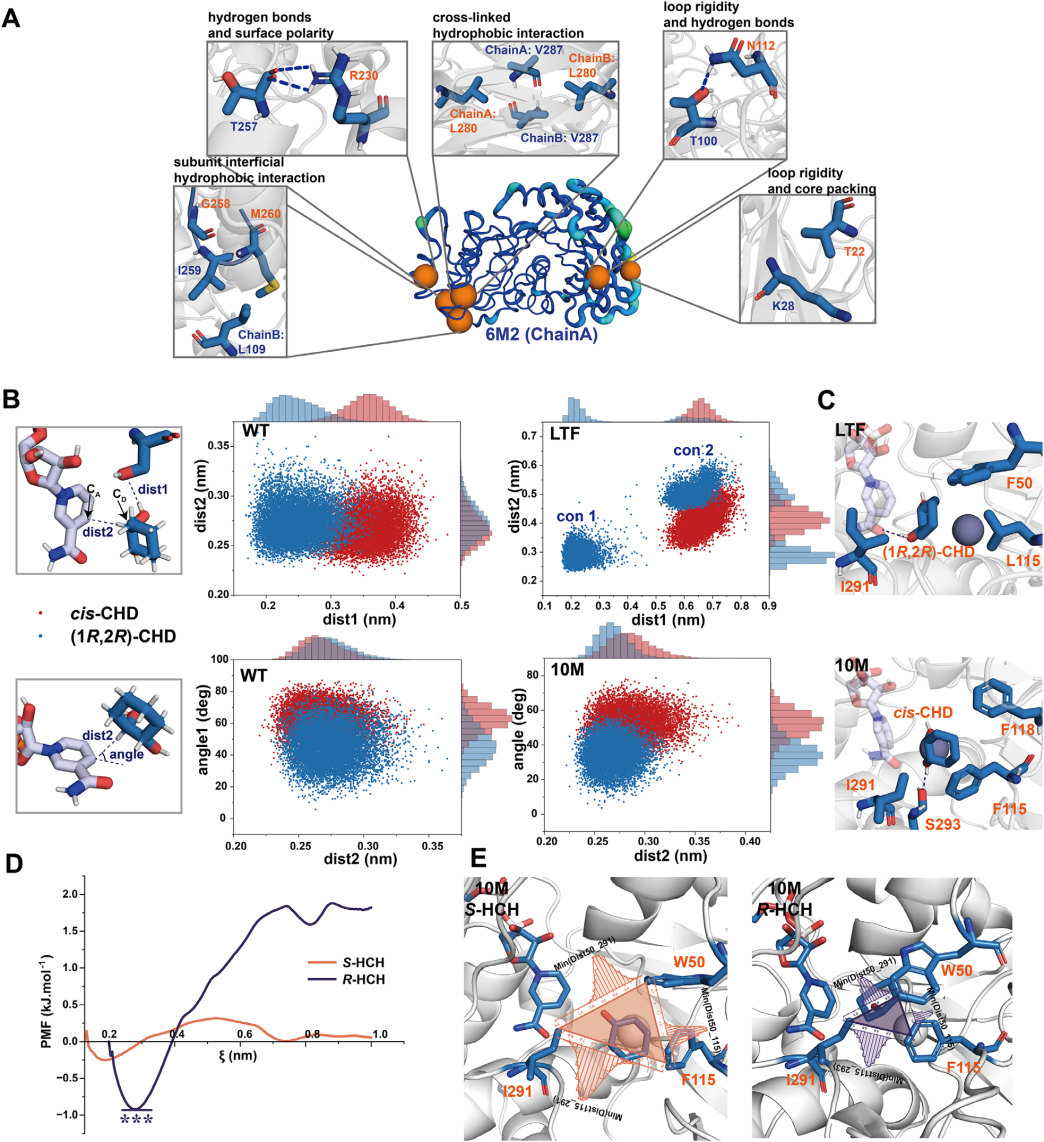
Molecular mechanisms of mutants with improved properties. (A) Structural basis of stabilization in the mutant 6M2. Q112N, A230R formed new hydrogen bonds, while T22V, Q112N, A260M, and I280L enhanced thermostability due to hydrophobic interactions. (B) Confirmation population analysis of WT, 10M, and LTF from the last 10 ns trajectories. The conformations of *cis*‐CHD (*meso‐*CHD, red) and (1*R*,2*R*)‐CHD (blue) were analyzed using dist1, dist2, and angle. (C) Substrate conformations in stereoselective mutants 10Mm and LTF. (D) Potential of Mean Force (PMF) analysis of *S*‐HCH and *R*‐HCH with 10M via umbrella sampling. (E) Area analysis of release tunnels for *S*‐HCH and *R*‐HCH in 10M.

We first conducted 100 ns MD simulations for WT and 6M2 without binding any ligands, and analyzed the conformation changes caused by the thermostable mutations. The RMSD showed that the trajectories equilibrated well after 20 ns (Figure S17A). We then analyzed the RMSF and radius of gyration (Rg) using the last 20 ns trajectory. The average Rg value of 6M2 was 0.31 Å lower than that of WT, indicating a more compact internal structure (Figure S17B). The RMSF values also showed that 6M2 was rigidified at the local regions of 18–30 aa, 75–90 aa, 110–113 aa, and 330–343 aa, where the mutations T22V, Q112N were located, suggesting that the mutations stabilized the local structure of the protein (Figure S17C). We analyzed changes in hydrogen bonds and hydrophobic interactions as well. A230R and Q112N formed stable hydrogen bonds with surrounding residues, resulting in 1.2 and 3.7 additional hydrogen bonds, respectively (Figure 6A; Figure S18). T258G contributed to the formation of a hydrophobic interaction with the interface mutation A260M, and T22V also formed a new hydrophobic interaction that increased loop rigidity. Interestingly, I280L utilized a Y‐type hydrophobic side chain at the interface to form cross‐linked hydrophobic interactions with V287 from chain A and chain B, thereby enhancing both interfacial and internal stability (Figure 6A). These interactions collectively enhanced the thermostability of 6M2.

MD simulations were also performed for 10M and LTF in complex with NAD^+^ and *cis*‐CHD or (1*R*,2*R*)‐CHD to investigate how the reshaped binding pockets influence the stereoselectivity. Based on the catalysis mechanism quantified by QM/MM study, the substrate conformation in the binding pocket was evaluated using three key indicators (Figure 6B): (1) dist1: the distance between the hydrogen atom of the hydroxyl group at the R‐configured stereocenter and the oxygen atom in the side chain of S39; (2) dist2: the distance of the hydride transfer from the donating carbon (C_D_) to the accepting carbon (C_A_); (3) angle: the hydride attack angle toward the nicotinamide ring of NAD^+^. Conformations from the last 10 ns trajectories were analyzed to calculate dist1, dist2, and angle. As for WT, there was a slight difference in dist2 between *cis*‐CHD and (1*R*,2*R*)‐CHD, measuring 0.26 and 0.27 nm, respectively. By contrast, the dist1 for *cis*‐CHD (0.35 nm) was larger than (1*R*,2*R*)‐CHD (0.25 nm). However, *cis*‐CHD exhibited a larger attack angle (62.8°) than (1*R*,2*R*)‐CHD (45.5°). Since experimental results showed that the activity of *cis*‐CHD was higher than (1*R*,2*R*)‐CHD, the angle might have a greater impact on the reaction.

In the binding pocket of 6M2/F115L/L118T/Y293F*_trans_* (LTF), two stable conformations of (1*R*,2*R*)‐CHD were observed (Figure 6B; Figure S19). The analysis of the two conformations revealed that con2 was the result of con1 being flipped by 180°, indicating that the interaction between the substrate and the binding pocket of LTF was weak. Further statistical analysis was performed on dist1, dist2, and the angle of the correct conformation (Figure 6B; Figure S20). The average reaction distances dist1 and dist2 of (1*R*,2*R*)‐CHD were 0.22 and 0.29 nm, both of which were within a reasonable distance for the reaction to occur. However, the average angle of (1*R*,2*R*)‐CHD was only 36.8° (Figure S20), which explained the low catalytic activity of LTF toward *trans*‐CHD. *cis*‐CHD exhibited only one stable conformation in the binding pocket (Figure S20), which was stabilized by the hydrophobic interactions provided by residues L115 and I291 (Figure S19). When (1*R*,2*R*)‐CHD was bound in the LTF binding pocket, its 2‐OH formed a hydrogen bond with the amide group of NAD^+^ (Figure 6C). However, the 2‐OH of *cis*‐CHD was oriented away from the amide group of NAD^+^. This caused *cis*‐CHD to deviate from the reactive conformation, with dist1 and dist2 values of 0.66 and 0.41 nm, respectively, which explained why LTF showed no activity toward *cis*‐CHD.

In order to understand the stereoselectivity mechanism of 6M2/Y293S/L118F/F50W/W269H*_cis_* (10M), we analyzed dist1, dist2, and angle as well. Both dist1 and dist2 for *cis*‐CHD and (1*R*,2*R*)‐CHD were within reasonable attacking distances (Figure 6B; Figure S20). However, the attack angle for (1*R*,2*R*)‐CHD measured 34.1°, significantly smaller than the 52.4° angle observed for *cis*‐CHD (Figure 6B). This resulted in the low catalytic activity of 10M toward (1*R*,2*R*)‐CHD and the high stereoselectivity for *cis*‐CHD.

We then investigated how the mutations influenced the release of the product. The product (*R*/*S*)‐HCH was docked into the binding pocket, and stable conformations were obtained through MD simulations. The umbrella sampling was performed on the stable complex conformation, which released the product HCH in the direction away from NADH. The potential of mean force (PMF) for the release process of HCH from the binding pocket was calculated using the weighted histogram analysis method (WHAM) (Figure 6D; Figure S21). During the release process of *R*‐HCH, the ∆PMF reached a maximum of 2.76 kJ/mol, indicating a significant free energy barrier that needed to be overcome. Moreover, there was an energy trap in the binding pocket for *R*‐HCH (ξ = 0.29 nm), which resulted in low turnover efficiency. By contrast, the release of *S*‐HCH was much easier, requiring a maximum barrier ∆PMF of only 0.56 kJ/mol.

This ∆PMF difference between the two products could be caused by the binding modes of substrates and the steric hindrance at position 50 (Figure 6E). As for *R*‐HCH, the substrate formed hydrophobic interactions with residues 50, 115, and 291, where *R*‐HCH acted as “glue”. Particularly, when F50 was mutated to a larger hydrophobic amino acid W, these hydrophobic interactions led to a narrow release tunnel with an average area of 3.94 Å^2^, which severely hindered the product release (Figure 6E; Figure S22). The enhanced hydrophobic interactions also stabilized the product‐protein interaction, accounting for the energy trap during product release. As for *S*‐HCH, the six‐membered ring was oriented toward W50 and F115, and only formed hydrophobic interactions with W50 and F115. Due to the absence of interaction between residue 291 and HCH, it was easy for residues 50 and 115 to move away from residue 291 and form a large release tunnel with an average area of 6.66 Å^2^, which created the condition for the smooth release of *S*‐HCH (Figure 6E; Figure S22).

### 2.7 Incorporation of Non‐Canonical Amino Acids to Reveal the Role of F50 and L118

**FIGURE 7:**
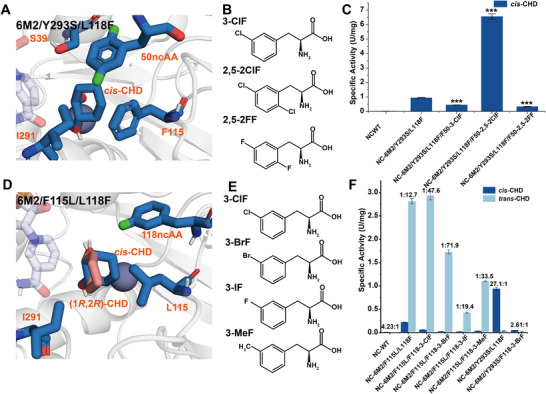
Incorporation of ncAAs for binding pocket and product release tunnel remodeling. (A) Structure of 6M2/Y293S/L118F with 2,5‐2ClF incorporated at position 50. (B) Structures of ncAAs incorporated at position 50 of 6M2/Y293S/L118F. (C) Activity and stereoselectivity of ncAA‐containing 6M2/Y293S/L118F mutants. The asterisks show statistically significant differences from NC‐6M2/Y293S/L118F. (D) Structure of 6M2/F115L with 3‐ClF incorporated at position 118. *cis*‐CHD and (1*R*,2*R*)‐CHD are colored in blue and pink, respectively. (E) Structures of ncAAs incorporated at position 118 of 6M2/F115L. (F) Activity and stereoselectivity of ncAA‐containing 6M2/F115L/L118F mutants. The ratio values of *cis*‐CHD:*trans*‐CHD are displayed above the columns. NC indicates *C*‐terminal His‐tag. The data were presented as mean values ± SD from three independent biological replicates (n = 3). The error bars represent the standard deviation (SD). Statistical significance was evaluated using one‐way analysis of variance (ANOVA). The asterisks of ^*^, ^**^, ^***^ denote *p* < 0.05, *p* < 0.01 and *p* < 0.001, respectively.

According to the stereoselectivity mechanism of 10M, residue W50 regulates product release by forming hydrophobic interactions with residues F115 and I291. During the release process of *S*‐HCH, residues W50 and F115 must dissociate from residue 291 to form an expanded product release tunnel. Therefore, we hypothesized that halogen atom modifications at position 50 could weaken its hydrophobic interaction with I291, thereby expanding the product release tunnel and potentially enhancing the activity toward *cis*‐CHD (Figure 7A). To validate the hypothesis, we chose 6M2/Y293S/L118F*_cis_* as the parent, which exhibited a *cis*:*trans* selectivity ratio of 28.1:1.

The genetic code expansion technology was applied to incorporate halogenated derivatives of *L*‐phenylalanine at position 50. In order to purify the whole ncAA‐containing protein without truncation, the His‐tag at the *N*‐terminus was changed to the *C*‐terminus. However, this modification (NC‐6M2) reduced the specific activity to 42% compared with the original *N*‐terminal His‐tag variant (Figure S23; Table 1).

When 3ClF was incorporated at position 50 of NC‐6M2/Y293S/L118F, the mutant NC‐6M2/Y293S/L118F/F50‐3‐ClF exhibited 52.3% lower activity toward *cis*‐CHD (Figure 7B,C). Substrate conformation analysis suggested that the chlorine atom may form a halogen bond [63] with residue S39 rather than orienting toward residues F115 and I291, which could explain the reduced activity (Figure S24). To prevent the interaction with residue 39, we incorporated 2,5‐2ClF, where 2‐Cl could form a halogen bond with the backbone amine of residue 50, thereby stabilizing the 5‐Cl orientation toward residues 291 and 115. The results demonstrated that NC‐6M2/Y293S/L118F/F50‐2,5‐2ClF exhibited a 6.98‐fold increase in *cis*‐CHD activity compared to 6M2/Y293S/L118F. This suggested that the halogen atom could play a crucial role in weakening hydrophobic interactions and enlarging the product release tunnel (Figure 7C). The measured kinetic parameters indicated that its catalytic efficiency for *cis*‐CHD increased to 1.020 ± 0.072 s^−1^mM^−1^, which was 214.48‐fold and 7.02‐fold higher than NC‐WT and NC‐6M2/Y293S/L118F, respectively (Figure S25; Table 1). Moreover, the incorporation of 2,5‐2FF resulted in a reduced catalytic activity, likely due to the strong polarity of the fluorine atoms (Figure 7B,C).

Previous computational simulations demonstrated subtle differences between the binding modes of *cis*‐CHD and (1*R*,2*R*)‐CHD. The six‐membered ring of *cis*‐CHD tended to orient toward residues 115 and 118, while (1*R*,2*R*)‐CHD preferred to form hydrophobic interactions with residues 115 and 291. We hypothesized that a halogen atom modification at the phenyl group of F118 would selectively reduce *cis*‐CHD binding affinity, thereby enhancing the stereoselectivity for *trans*‐CHD. 6M2/F115L/L118F was selected as the parent due to its high activity toward *trans*‐CHD but moderate stereoselectivity (−0.806), which was lower than ‐0.935 of 6M2/F115L*_trans_* (Figure 3C,E). To validate the hypothesis, we incorporated 3‐ClF, 3‐BrF, 3‐IF, and 3‐MeF at position 118 of NC‐6M2/F115L/L118F and successfully improved stereoselectivity (Figure 7D,E). Notably, 3‐BrF showed the highest stereoselectivity with a *trans*:*cis* selectivity ratio of 71.9:1, which was significantly higher than 25.7:1 of 6M2/F115L*_trans_* (Figure 2E and 7F). The incorporation of 3‐MeF also resulted in an improved stereoselectivity (33.5:1) compared to NC‐6M2/F115L/L118F (12.6:1), indicating that steric hinderance could also be used for improving the stereoselectivity. Conversely, incorporating these ncAAs at position 118 in the *cis*‐CHD preference variant 6M2/Y293S/L118F*_cis_* would decrease its stereoselectivity for *cis*‐CHD. The result showed that NC‐6M2/Y293S/L118ClF decreased its stereoselectivity from 27.1:1 to 2.61:1 (Figure 7F). These results confirmed the role of residue 118 in modulating enzyme stereoselectivity and highlighted the importance of ncAAs incorporation in elucidating enzyme mechanisms.

## 3 Conclusion

In this study, we engineered *Bs*BDH to improve and invert its stereoselectivity using several strategies, including protein language model, perfect iterative saturation mutagenesis, and the incorporation of ncAAs. To obtain a stable scaffold for the evolution of stereoselectivity, we developed a PASS (Position Analysis and Source Search) strategy to filter single mutations predicted by ∆∆G calculations. The PASS strategy effectively addressed the high false‐positive rate, which was a common issue in many thermostability design methods such as PROSS and GRAPE. As a result, we obtained several highly selective variants: a *trans*‐CHD preference variant LTF (*ee* > 99%) and a *cis*‐CHD preference variant 10M (*ee* > 99%). The incorporation of ncAAs overcame the limitations of natural amino acids, and illustrated the critical role of F50 and L118 in modulating enzyme activity and stereoselectivity. The engineered *Bs*BDH variants demonstrate promising potential for the biocatalytic production of *α*‐hydroxyketones in future applications.

## 4 Experimental Section

### 4.1 Microorganisms and Materials

*Bs*BDH, *Bs*NOX, and *Mb*PylRS used in this work are preserved in the laboratory and expressed in the host *E.coli* BL21(DE3). The expression vectors used in this study were pET‐28a(+), pET‐Duet‐1, and pULTRA. *cis*‐1,2‐cyclohexanediol and *trans*‐1,2‐cyclohexanediol were purchased from Meryer Co., Ltd. (Shanghai, China). 2‐Hydroxycyclohexanone, and ncAAs were purchased from Bide Pharmatech Ltd. (Shanghai, China). (2*R*,3*R*)‐Butanediol was provided by Shanghai Aladdin Biochemical Technology Co., Ltd. (China). DNA polymerase (PrimeStar Max) and QuickCut Dpn I were purchased from Takara Biomedical Technology (Beijing, China) Co., Ltd.

### 4.2 Site‐Directed Mutagenesis

Mutations were introduced into the pET28a‐SUMO‐*Bs*BDH plasmid using the PrimeSTAR mutagenesis basal kit (Takara) with specially designed primers. All mutants were constructed with the utility of iBioFoundry. The PCR mixture consisted of 0.5 µL of the DNA template (50 ng/µL), 1 µL of each primer (10 µm), 10 µL of ddH2O, and 12.5 µL of 2×PrimeSTAR in a total volume of 50 µL. The PCR products were incubated with QuickCut Dpn I at 37°C for 30 min to digest the original DNA template. 2 µL of the digested solution was added to 20 µL competent cells by an automation workstation (Evo, Tecan Trading AG, Switzerland). After incubating at 4°C for 30 min, a heat shock at 42°C for 90 s was performed. The samples were then instantly placed on ice for 2 min. Following this, 200 µL of Luria‐Bertani (LB) medium was added to each sample, and they were incubated in a shaker for 1 h. Transformant plating was performed by spraying the cells onto eight‐well agar plates using the 8‐channel pipetting system in the automated workstation (Fluent, Tecan Trading AG, Switzerland). The plates were labeled with a microplate labeler (Agilent Labeler, Santa Clara, CA, USA) and then incubated in the automated incubator (Cytomat 10C, ThermoFisher, Waltham, MA, USA) for 14 h. The entire mutant genes were sequenced to confirm that no other changes were introduced into the nucleotide sequences.

### 4.3 Expression and Purification of *Bs*BDH and Mutants

The *Bs*BDH and mutants were cultured in LB media at 37°C to an OD_600_ of 0.6–0.8, and then induced by 1 mm isopropyl *β*‐D‐thiogalactopyranoside (IPTG) at 18°C for 18 h. Cells were harvested by centrifugation at 4000 rpm for 15 min. The cell pellet was resuspended in lysis buffer (50 mm Tris, pH 8.0) and disrupted by ultrasonication. The supernatant was obtained by centrifugation at 12 000 rpm, 4°C for 30 min to remove the cell debris. Protein was purified by Ni‐NTA resin. The purified enzyme was verified by SDS‐PAGE and stored at 4°C. Additionally, the protein concentration was determined by the BCA protein assay kit (Takara Biomedical Technology (Beijing) Co., Ltd.).

### 4.4 Enzyme Activity and Stereoselectivity Measurement

The catalytic activity of the enzyme was measured by following either the oxidation of NADH or the reduction of NAD^+^ at 340 nm. The oxidation‐reduction reactions were carried out in 200 µL 50 mm Tris buffer (oxidation reaction: pH 8.5, reduction reaction: pH 7.5) containing 12.5 mm substrate and an appropriate of enzyme. One unit of the activity was defined as the amount of enzyme that oxidized or reduced 1 µmol NADH or NAD^+^ per minute. In this research, the stereoselectivity value was defined as (SA*_cis_*‐SA*_trans_*)/(SA*_cis_* +SA*_trans_*) to quantitatively reflect the catalytic preference between *cis*‐CHD and *trans*‐CHD, where SA*_cis_* and SA*_trans_* are the specific activities toward *cis*‐CHD and *trans*‐CHD, respectively.

### 4.5 Kinetic Parameters Measurement

Kinetic parameters were determined by a series of concentrations (0.125, 0.25, 0.625, 1.25, 1.875, 2.5, 3.125, 3.75, 5, 7.5, 10, 12.5, 18.75, 25 mm) of *cis*‐CHD or *trans*‐CHD in the presence of 0.50 mm NAD^+^ in 50 mM Tris buffer (pH 8.5). *K*_m_ and *k*_cat_ were determined by fitting the Michaelis–Menten equation. The results were presented as the mean ± standard deviation (n = 3).

### 4.6 Enzyme Residual Activity and Half‐Life Assays

The enzyme residual activity was assayed by incubating the enzyme at 37°C. Enzyme samples collected at different time points were quantitatively measured. A first‐order deactivation rate constant (*k*_d_) was determined by linear regression of ln(residual activity) vs. incubation time (*t*). The half‐life at 37°C was calculated using the equation *t*_1/2_ = ln(2)/*k*_d_.

### 4.7 Resting Whole‐Cell Biotransformation

Two plasmids, pET28a‐SUMO‐*Bs*BDH(mutants) and pET‐Duet‐1‐*Bs*NOX were co‐transformed into *E.coli* BL21(DE3). pET‐Duet‐1‐*Bs*NOX was introduced to express *Bs*NOX. NOX‐*Bs*BDH and its mutants were cultured in LB media at 37°C until reaching an OD_600_ of 0.6–0.8, and then induced by 1 mm isopropyl *β*‐D‐thiogalactopyranoside (IPTG) at 18°C for 18 h. Cell densities were measured by OD_600_, and cells were harvested by centrifugation at 4000 rpm for 15 min. Resting cells were resuspended to target cell densities (NOX‐*Bs*BDH and NOX‐10M: OD_600_ 5; NOX‐LTF: OD_600_ 30) in the reaction buffer (50 mm Tris pH 8.0, and 10 mm mixed CHD as substrate). The substrate mixed CHD is an equimolar (1:1:1) mixture of *cis*‐CHD, (1*R*,2*R*)‐CHD, (1*S*,2*S*)‐CHD, which was prepared by mixing *cis*‐CHD and *trans*‐CHD with a molar ratio of 1:2. Whole‐cell bioconversion was performed in 50 mL conical tubes containing 5 mL of resuspended cells at 30°C for 180 min. 200 µL samples were withdrawn at each time point for HPLC analysis.

### 4.8 HPLC Analysis

The products *R*‐HCH and *S*‐HCH were detected by a 1260II system (Agilent, China) equipped with a YMC CHIRAL ART Amylose‐SA (250 × 4.6 mm, 5 µm, YMC Co, Ltd, Kyoto, Japan). 85% mobile phase A (0.1% trifluoroacetic acid in distilled water) and 15% mobile phase B (acetonitrile) were used at 0.5 mL/min. Separation was performed at 35°C with detection at 254 nm.

### 4.9 Non‐Canonical Amino Acids Incorporation

Two plasmids, pET28a‐SUMO‐*Bs*BDH(mutants) and pULTRA‐*Mb*PylRS‐IPYE were co‐transformed into *E.coli* BL21(DE3). pULTRA‐MbPylRS‐IPYE was used for expressing the *Mb*PylRS mutant IPYE. Positions for incorporating ncAAs were mutated to the amber codon (TAG). Cells were added into LB media (5 mL) containing kanamycin (50 µg/mL) and spectinomycin (50 µg/mL), cultured at 37°C overnight, and then inoculated into GMML media (100 mL). IPTG (the final concentration of 1 mm) and ncAAs (the final concentration of 1 mm) were added into the GMML when OD_600_ reached 0.6–0.8. Cells were induced at 18°C for 18 h. The enzymes were purified using Ni‐NTA resin. The purified enzymes were concentrated to 200 µL using ultrafiltration. The molecular weight was verified by SDS‐PAGE.

### 4.10 Protein Structure and Molecular Docking Method

The structure of *Bs*BDH was obtained from the RCSB Crystal Database (PDB ID: 6IE0). AlphaFold3 [64] (https://deepmind.google/technologies/alphafold/alphafold‐server) was used to determine the binding conformation of the coenzyme with *Bs*BDH. Then, substrates *cis*‐CHD, (1*R*,2*R*)‐CHD, *S*‐HCH, and *R*‐HCH were docked into *Bs*BDH manually according to the reaction mechanism in Pymol (version 3.1.1). The substrate binding conformations were optimized by MD energy minimization. Virtual mutations and structure visualization were performed by Pymol. Additionally, the predicted binding sites for the substrates *cis*‐CHD and (1*R*,2*R*)‐CHD were recognized by the Autodock Vina software. The docking was carried out according to the manufacturer's protocol with default settings.

### 4.11 Molecular Dynamics (MD) Simulation Analysis of BsBDH Mutants

The crystal structure of the tetramer 6IE0 without any binding ligands was used as the input of MD simulations for the thermostable mutations design and analysis. The complex structure of a monomeric protein with NAD^+^, predicted by AlphaFold3, was used for MD simulations to illustrate the mechanism by which the variants improve stereoselectivity. These MD simulation systems contained *Bs*BDH or its mutants, a cofactor (NAD^+^), and a substrate (*cis*‐CHD, (1*R*,2*R*)‐CHD, *S*‐HCH, or *R*‐HCH). MD simulations were performed using CHARMM36 protein force field parameters within the GROMACS 2023 package [65, 66]. The force field parameters for NAD^+^, *cis*‐CHD, (1*R*,2*R*)‐CHD, *S*‐HCH, and *R*‐HCH were constructed by the CGenFF web server using the CHARMM general force field [67]. The complex was centralized in a rectangular box that has a 1 nm distance to the largest protein boundary. The box was filled with the TIP3P water model. Sodium ions were added to the solution to maintain the system's neutrality. Energy minimization was performed using the steepest descent algorithm. A total of 1000 steps of energy minimization were carried out, and the conformation with the lowest energy was taken as the initial conformation for the pre‐equilibrium stage. During the pre‐equilibrium stage, a 100 ps NPT ensemble was employed to equilibrate the system at 298.15 K and 1 atm using the Berendsen pressure coupling and the velocity‐rescaling thermostat. The 100 ns MD simulation was carried out under the conditions: 298.15 K, 1 atm. To maintain the pressure, the Parrinello‐Rahman pressure coupling was utilized in the production run, with pressure time constant and isothermal compressibility set to 1 ps and 4.5 × 10^−5^ bar^−1^. A cutoff of 10 Å was used for non‐bonded interactions. The root‐mean‐square fluctuations (RMSF) of backbone atoms were analyzed with the last 10 ns trajectories.

### 4.12 Umbrella Sampling of the Product HCH Release

Umbrella sampling was used for exploring the mechanism of the stereospecific mutant 10M [68]. 50‐ns MD simulation was first carried out for obtaining conformations of the substrate. In order to generating a series of conformations along the reaction coordination, umbrella sampling was conducted. Harmonic potential was used for pulling the products away from the binding pocket, and the force constant 1000 kJ.mol^−1^.nm^−2^ was used for pulling. The spacing of the windows was defined about 0.5 Å, and the frames were extracted from the pulling trajectory, which were used for umbrella sampling input. The extracted frames were used for independent simulations. Then, PMF was analyzed by the Weighted Histogram Analysis Method (WHAM).

### 4.13 Protein Thermostability Prediction and Position Frequency Matrix (PFM) Construction

In the initial step, two websites (GRAPE and PROSS) were used for ΔΔG calculations. GRAPE (https://nmdc.cn/grape‐web/) was based on three algorithms, with the cutoffs −5 R.E.U, −5 A.E.U, and −2.2 kJ/mol for Rosetta_ddg, ABACUS and FoldX, respectively. PROSS (http://pross.weizmann.ac.il/) was used with default parameters. Blastp search (protein‐protein BLAST) was used for collecting 5000 homologous sequences using the sequence of *Bs*BDH as the query sequence and NR (Non‐Redundant Protein Sequence Database) as the BLAST database. The blastp search was conducted with the parameters of expect threshold 0.05, word size 5, scoring matrix BLOSUM 62. Cd‐hit was used to remove ‘redundant’ (or highly similar) sequences. 696 sequences were selected after cd‐hit and subsequently classified by thermostability using TemBERTure, which was a deep learning model that predicted a protein's thermostability classification and melting point from amino acid sequences. The model was downloaded from https://github.com/ibmm‐unibe‐ch/TemBERTure, and implemented in a python env (python 3.9.18). Multiple sequence alignment was performed using ClustaW in MEGA11 and statistically calculated PMF.

### 4.14 Statistical Analysis

Data were presented as mean values ± SD from three independent biological replicates (n = 3). The significant differences were analyzed by GraphPad Prism 9.0 using one‐way analysis of variance (ANOVA). A *p*‐value of < 0.05 was considered statistically.

## Funding

The “Pioneer” and “Leading Goose” R&D Program of Zhejiang (Grant No. 2025C01097), National Natural Science Foundation of China (Grant No. 22378351), Key Research and Development Program of China (Grant No. 2022YFA0913000).

## Conflicts of Interest

The authors declare no conflicts of interest.

## Supporting information




**Supporting File**: advs73996‐sup‐0001‐SuppMat.pdf.

## Data Availability

The data that support the findings of this study are available from the corresponding author upon reasonable request.
