# Supplementary material for: Protein Language Model‐Guided Engineering of a 2,3‐Butanediol Dehydrogenase for the Enantioselective Synthesis of Cyclic α‐Hydroxy Ketones
Source: Adv Sci (Weinh). 2026 Jan 25;13(18):e09314. doi: 10.1002/advs.202509314 (PMC13042536; doi:10.1002/advs.202509314)
Supplement: Supplementary file 1 — Supporting File: advs73996‐sup‐0001‐SuppMat.pdf. [file ADVS-13-e09314-s001.pdf]

WILEY-VCH

Supporting Information

Protein language model-guided engineering of a 2,3-butanediol dehydrogenase for the enantioselective synthesis of cyclic α-hydroxy ketones

Haote Ding[#], *Ling Jiang*[#], *Yijia Song, Zhongji Pu, Lirong Yang, Haoran Yu**

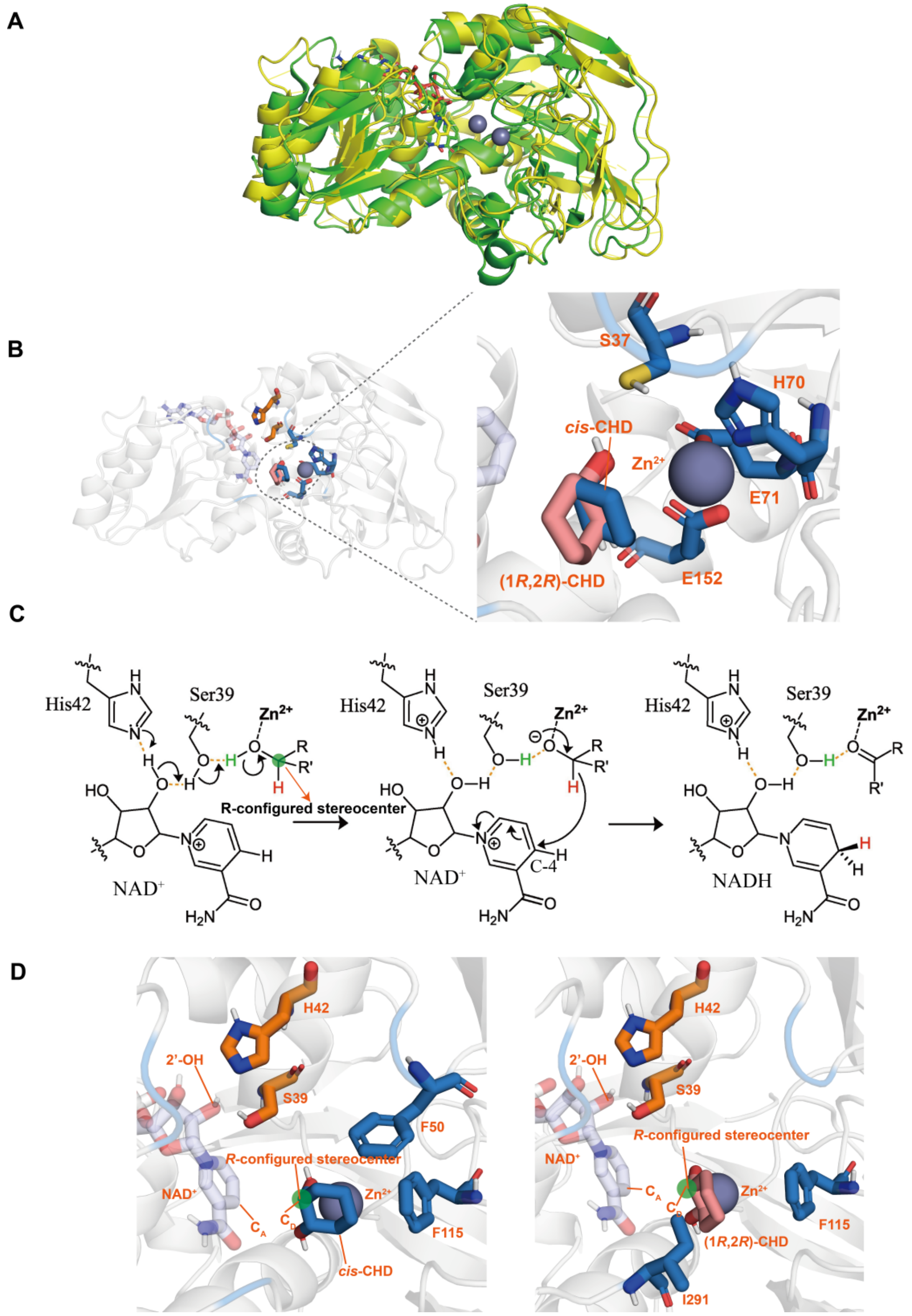

Figure S1. Catalytic residues in the *Bs*BDH structure. A) Structure alignment between the complex structure predicted by Alphafold3 (yellow) and the structure of the alcohol dehydrogenase from *Sulfolobus solfataricus* (green). B) Zn^{2+} binding site of *Bs*BDH with docked substrates: *cis*-CHD (*meso-CHD*, blue) and (1*R*,2*R*)-CHD (pink). C) General catalytic mechanism of zinc-dependent alcohol dehydrogenases. D) Substrate binding pocket of *Bs*BDH. C_D denotes the donating carbon at the *R*-configured stereocenter, while C_A represents the accepting carbon (the C-4 of the nicotinamide ring of NAD^+).

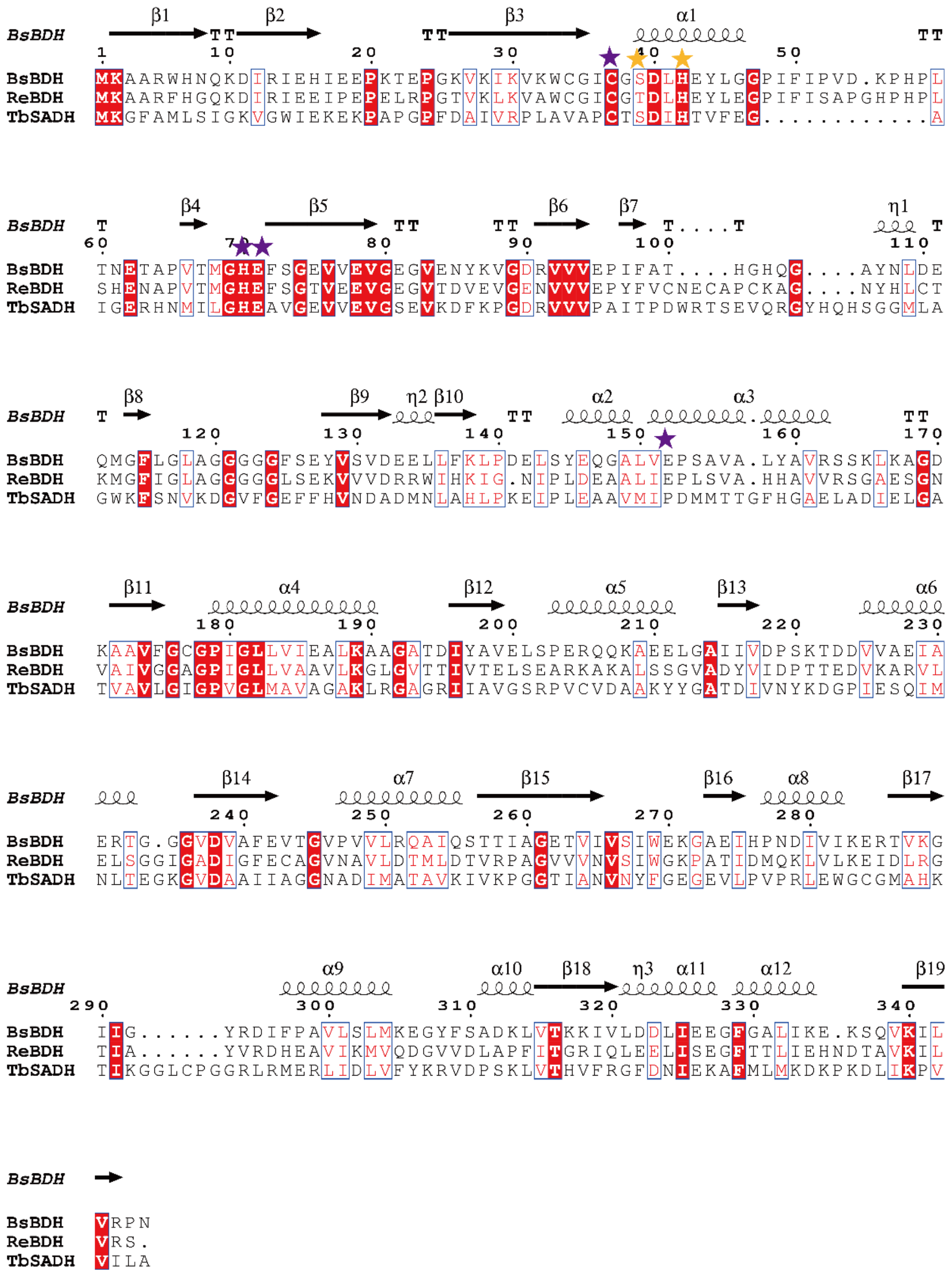

Figure S2. Sequence alignment and identification of key residues. Catalytic residues are marked with yellow stars, and Zn^{2+} coordinating residues are marked with purple stars.

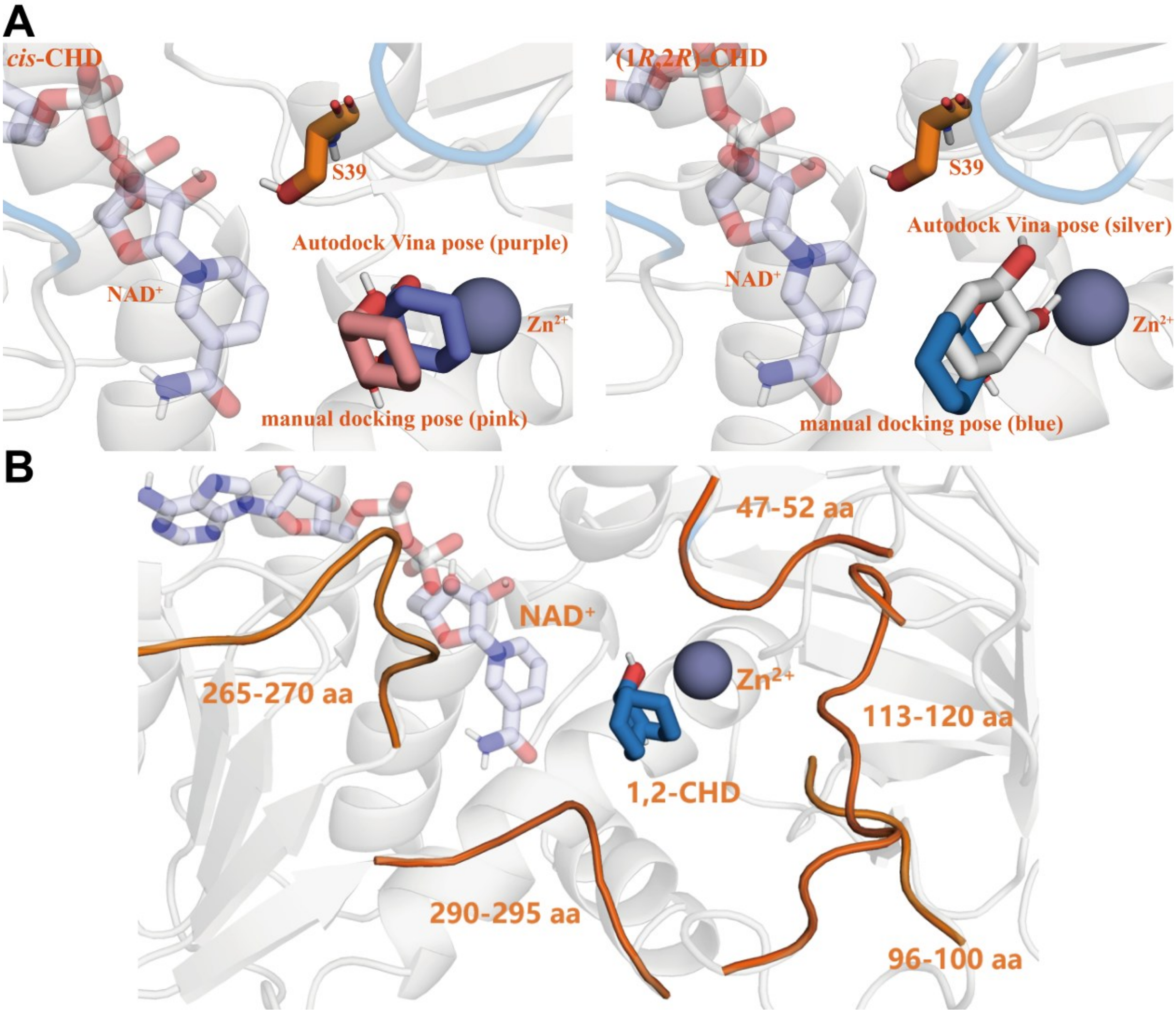

Figure S3. Analysis of the substrate binding pocket. A) The comparation between Autodock Vina docking and manual docking results. B) The substrate binding pocket. It is formed by five loops: 47-52 aa, 96-100 aa, 113-120 aa, 265-270 aa, and 290-295 aa.

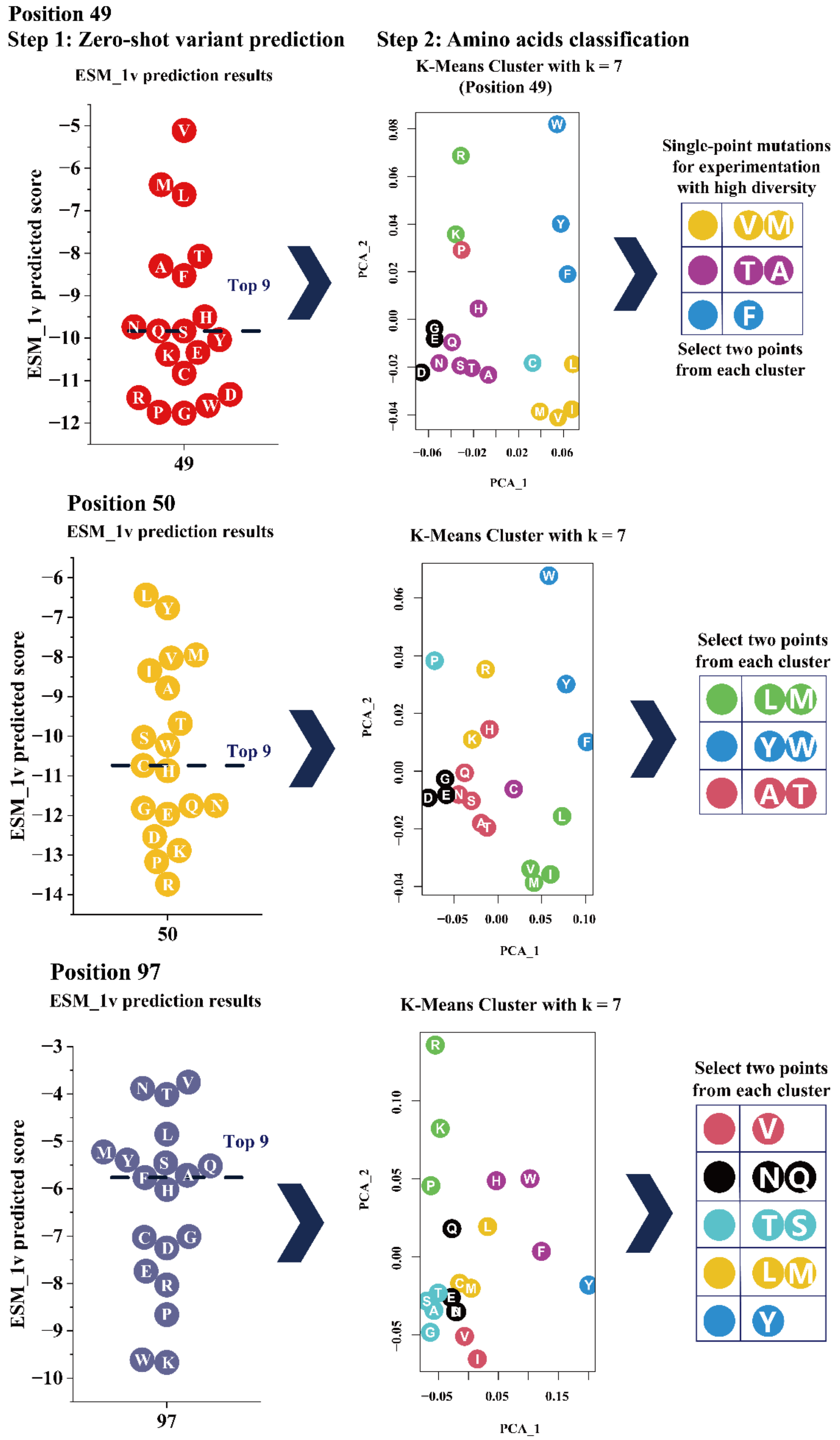
Position 49
Step 1: Zero-shot variant prediction
ESM_1v prediction results
ESM_1v predicted score
Top 9
49
Step 2: Amino acids classification
K-Means Cluster with k = 7
(Position 49)
PCA_1
PCA_2
Single-point mutations
for experimentation
with high diversity
Select two points
from each cluster
Position 50
ESM_1v prediction results
ESM_1v predicted score
Top 9
50
K-Means Cluster with k = 7
PCA_1
PCA_2
Select two points
from each cluster
Position 97
ESM_1v prediction results
ESM_1v predicted score
Top 9
97
K-Means Cluster with k = 7
PCA_1
PCA_2
Select two points
from each cluster

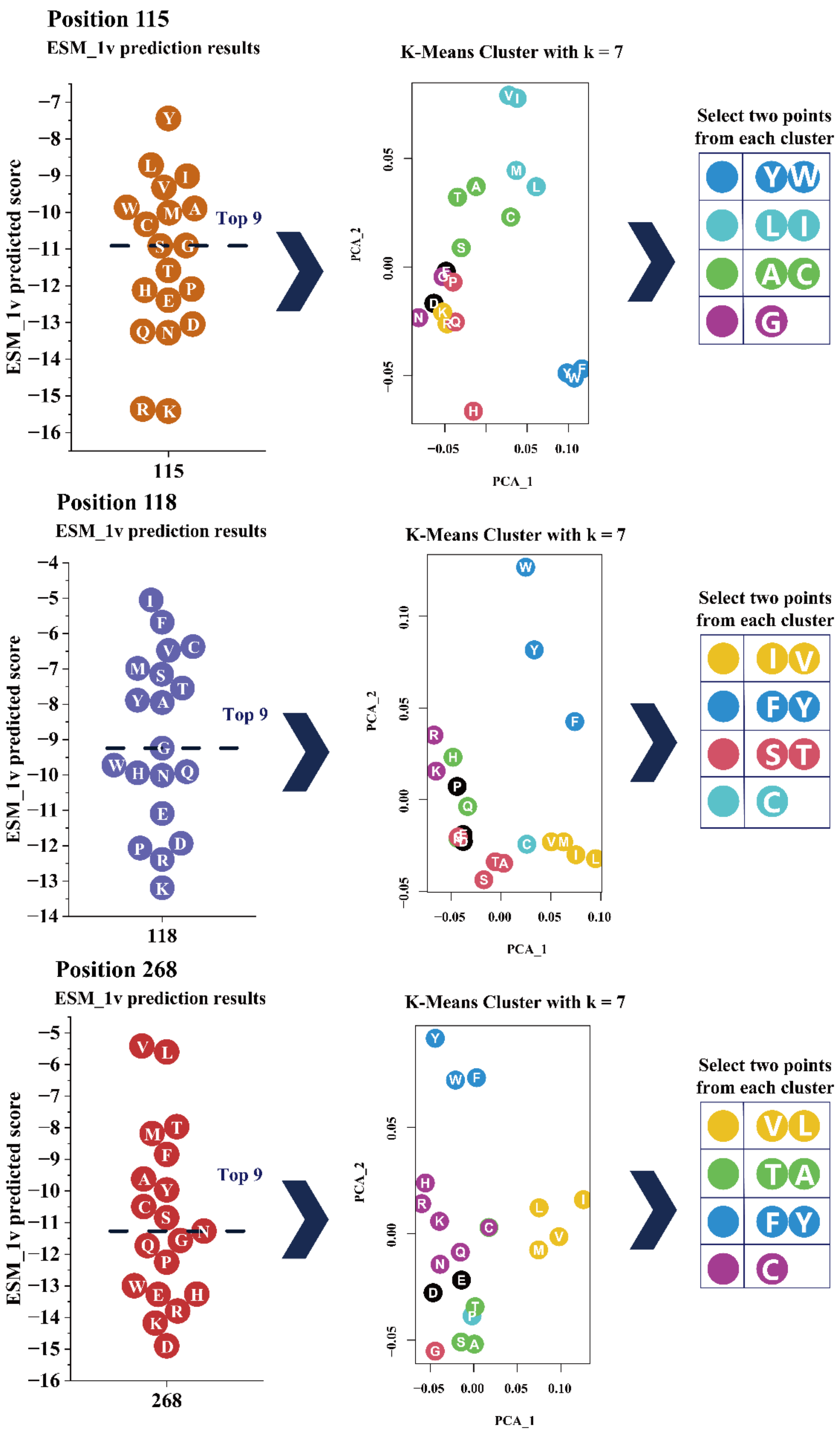

Position 115
ESM_1v prediction results
ESM_1v predicted score
Top 9
115
K-Means Cluster with k = 7
PCA_1
PCA_2
Select two points from each cluster
Position 118
ESM_1v prediction results
ESM_1v predicted score
Top 9
118
K-Means Cluster with k = 7
PCA_1
PCA_2
Select two points from each cluster
Position 268
ESM_1v prediction results
ESM_1v predicted score
Top 9
268
K-Means Cluster with k = 7
PCA_1
PCA_2
Select two points from each cluster

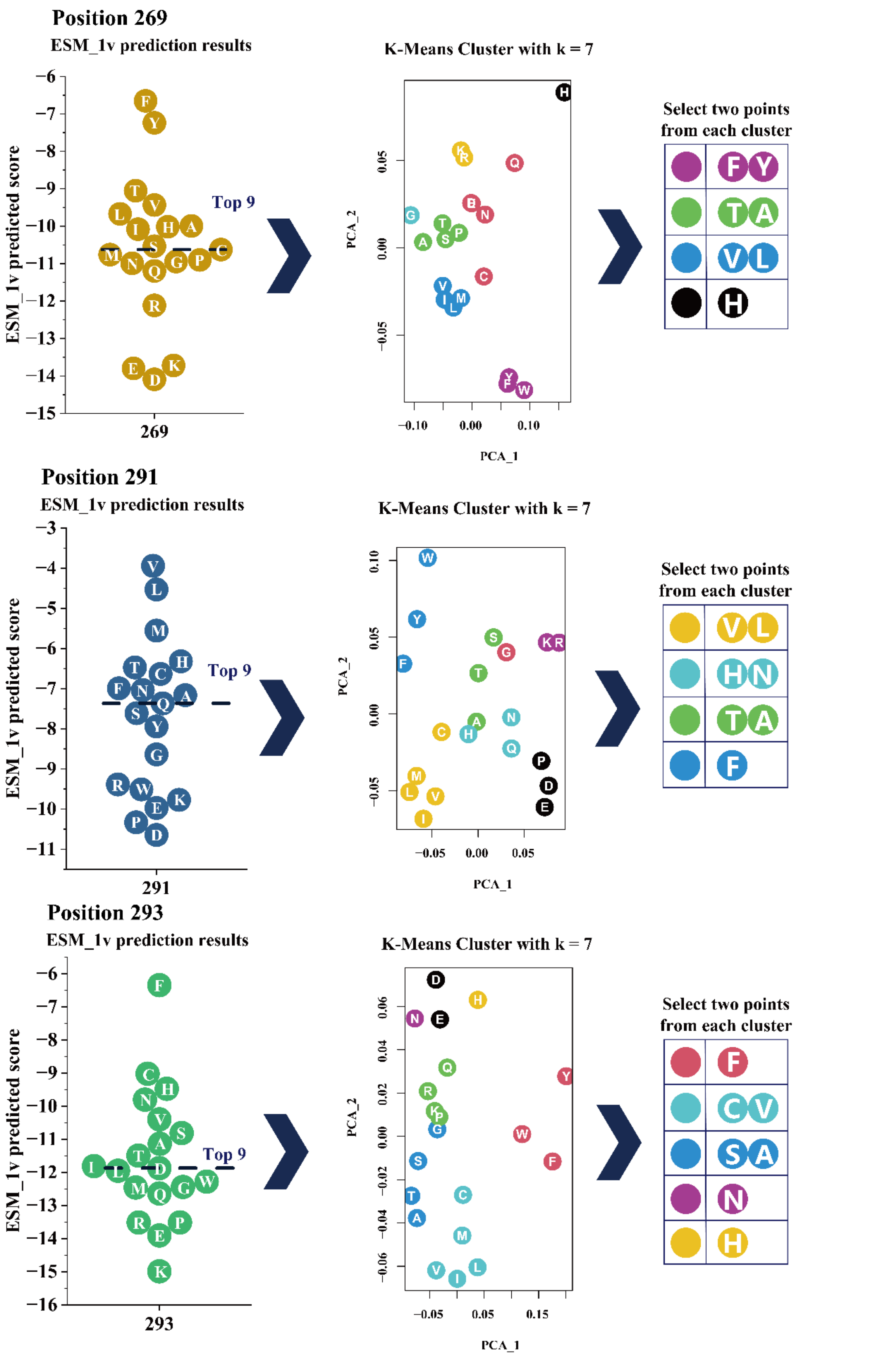

Figure S4. Zero-shot prediction of potential single-point mutations at position 49, 50, 97, 115, 118, 268, 269, 291, and 293 using the ESM-1v model.

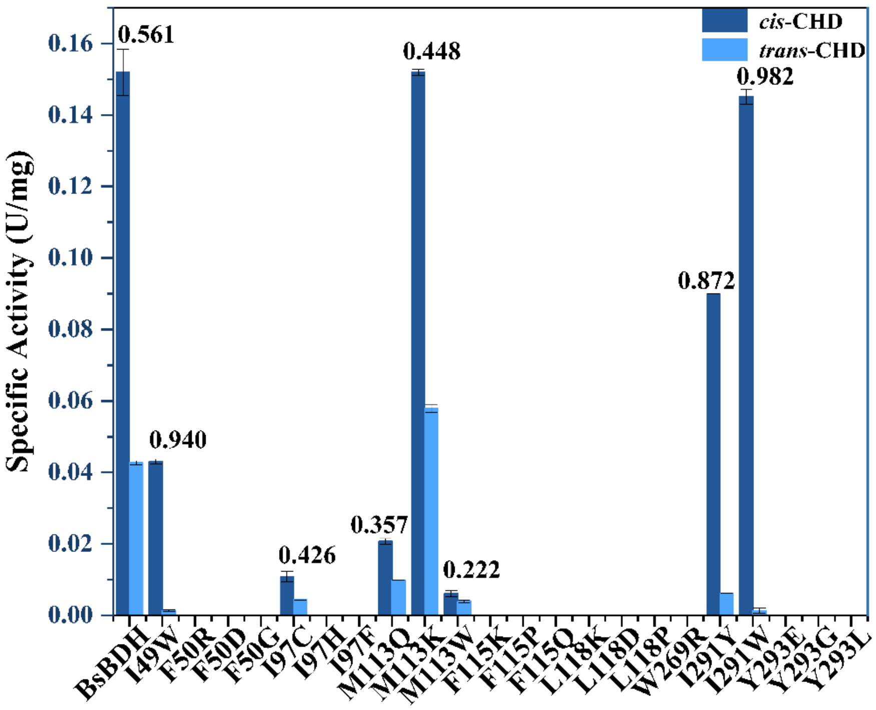

Figure S5. Activities of single-point variants with low ESM-1v prediction scores. The values above the column represent the stereoselectivity values. Stereoselectivity value was calculated by $(SA_{cis}-SA_{trans})/(SA_{cis}+SA_{trans})$, where SA_{cis} and SA_{trans} represent the specific activities against *cis*-CHD and *trans*-CHD, respectively. The data were presented as mean values ± SD from three independent biological replicates (n=3). The error bars represent the standard deviation (SD).

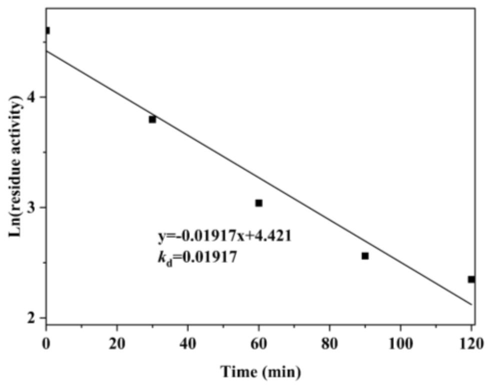

Figure S6. Half-life of wild-type *Bs*BDH at 37 ºC. The half-life of 36.16 min was calculated using the equation $t_{1/2}$=ln2/k_d, where the k_d is the deactivation rate.

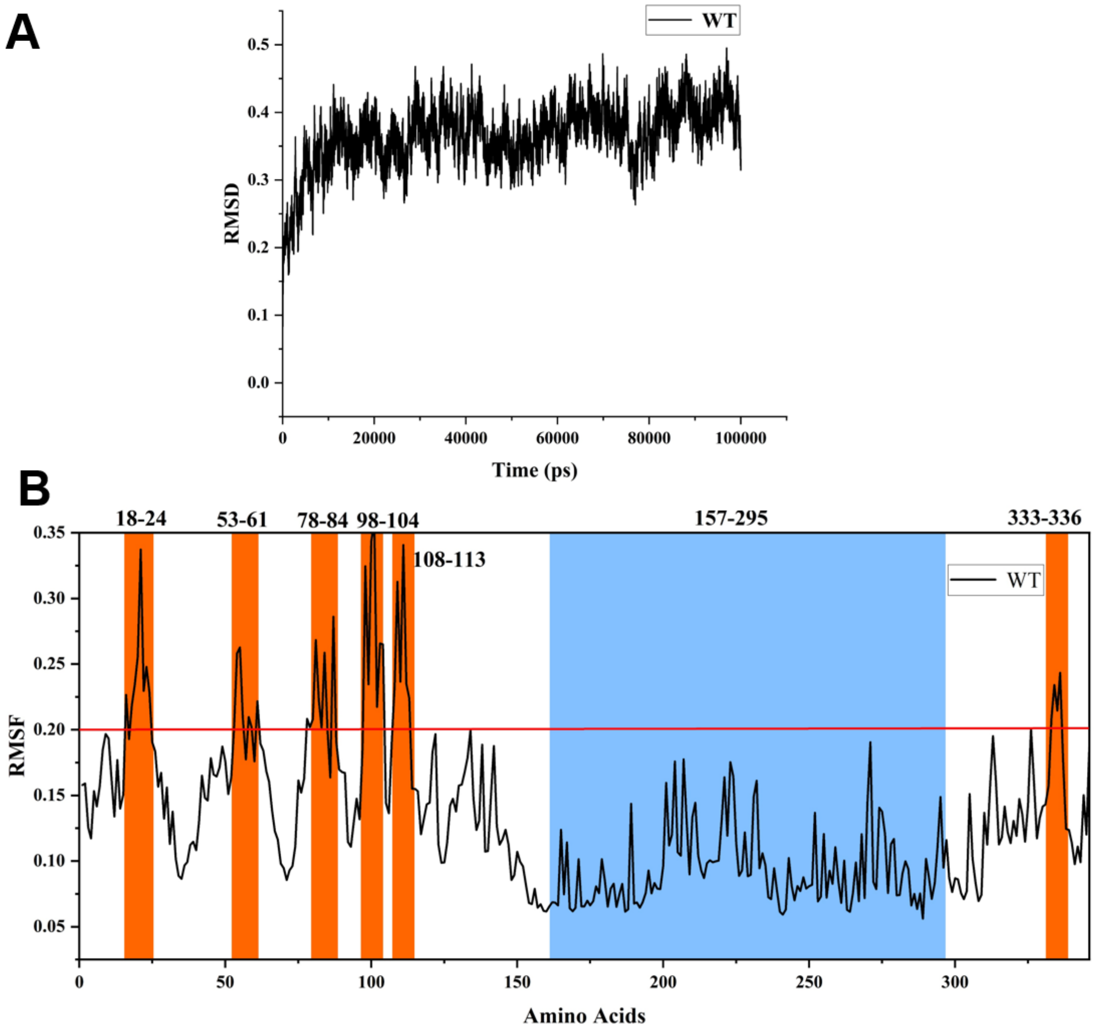

Figure S7. RMSD and RMSF analysis of wild-type *Bs*BDH. A 100-ns molecular dynamics simulation was carried out, and the last 20 ns trajectories were used for analysis.

419 non-thermophilic sequences

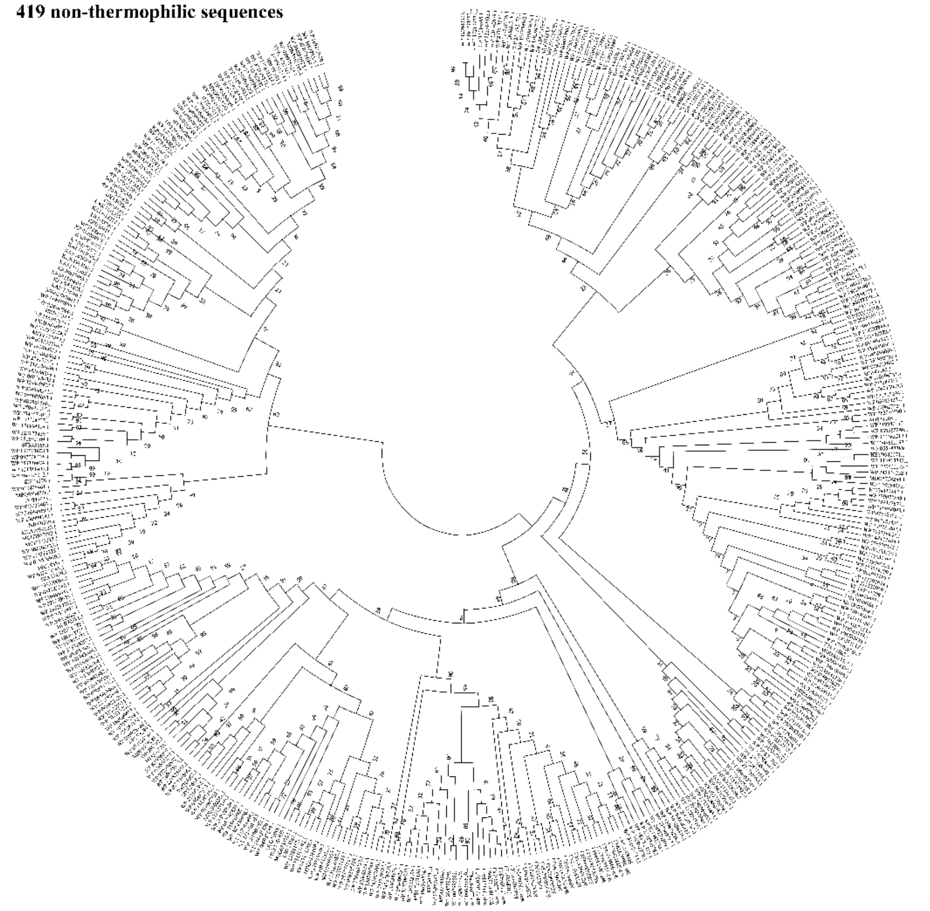

272 thermophilic sequences

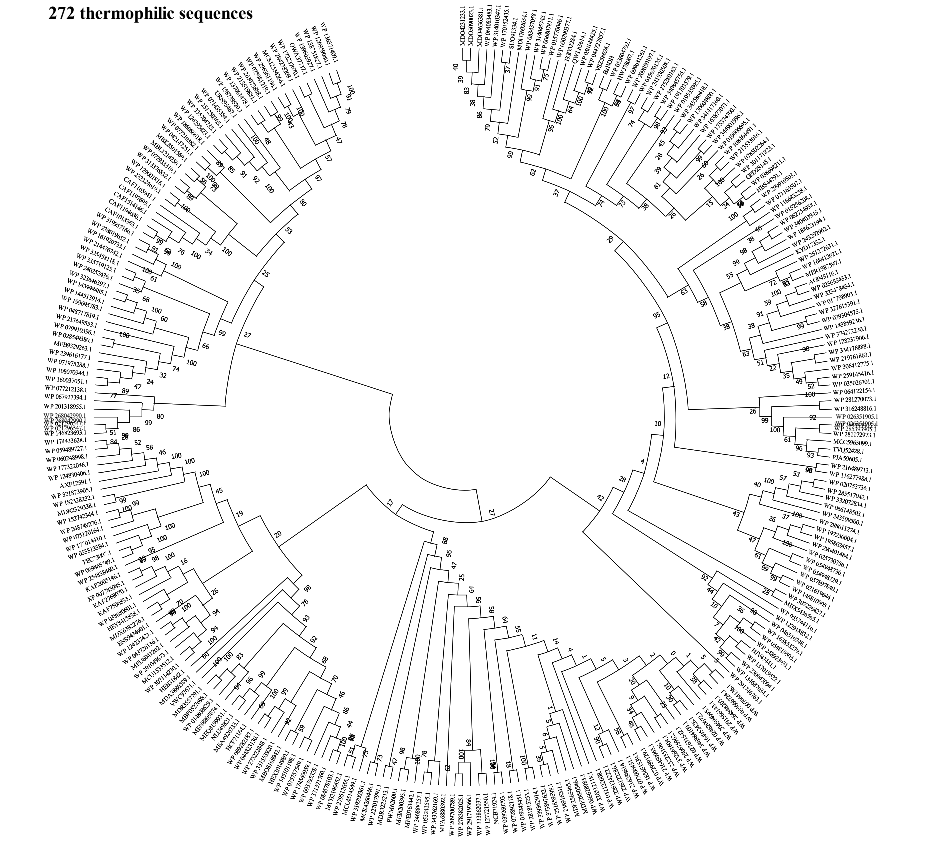

Figure S8. Thermophilic and non-thermophilic protein sequence libraries.

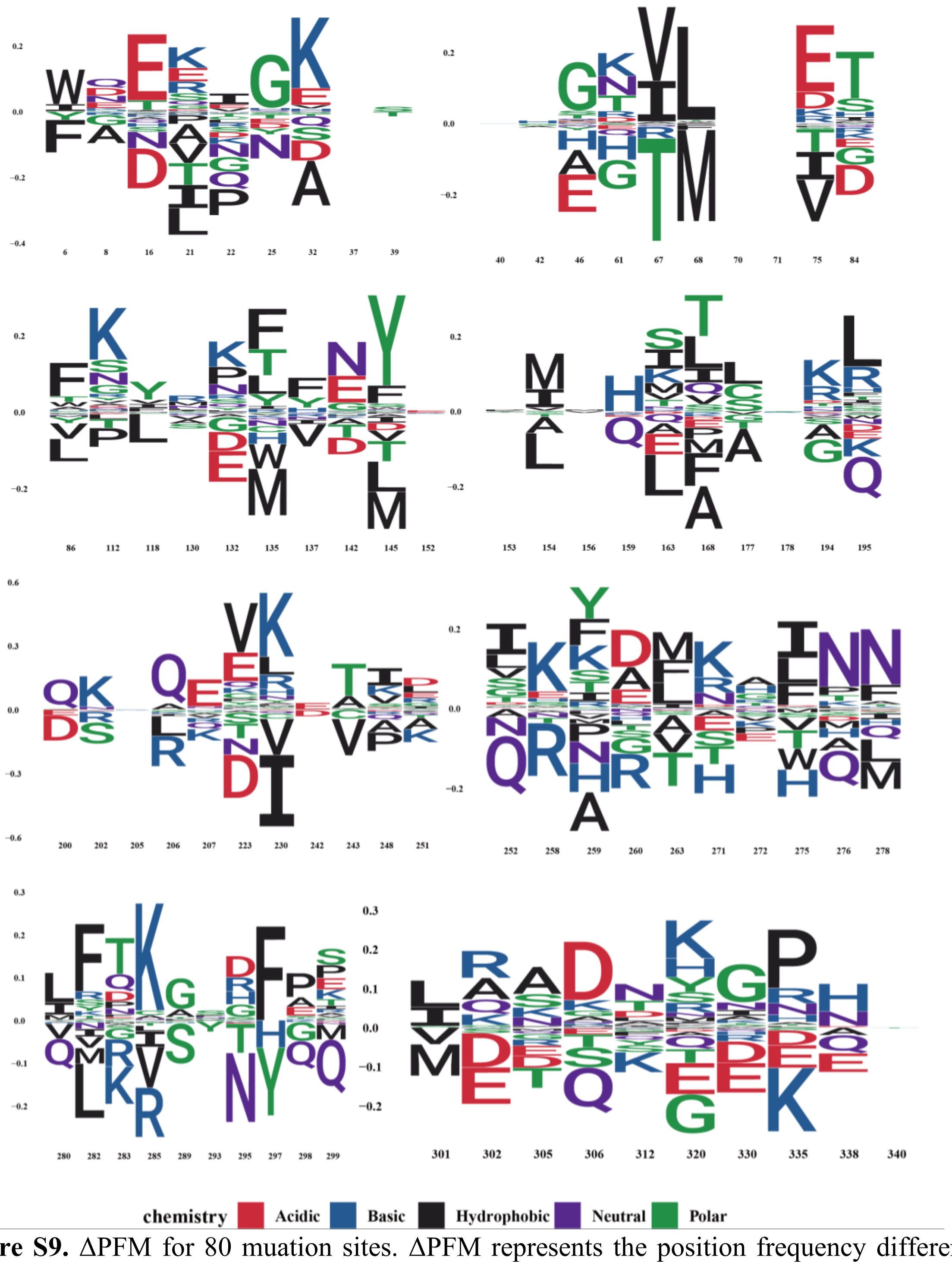

Figure S9. ΔPFM for 80 muation sites. ΔPFM represents the position frequency difference between PFM(thermophilic sequences) and PFM(non-thermophilic sequences). ΔPMF > 0 suggests higher amino acid frequencies in thermophilic sequences. Conversely, ΔPMF < 0 indicated that the amino acid appears less frequently in thermophilic sequences.

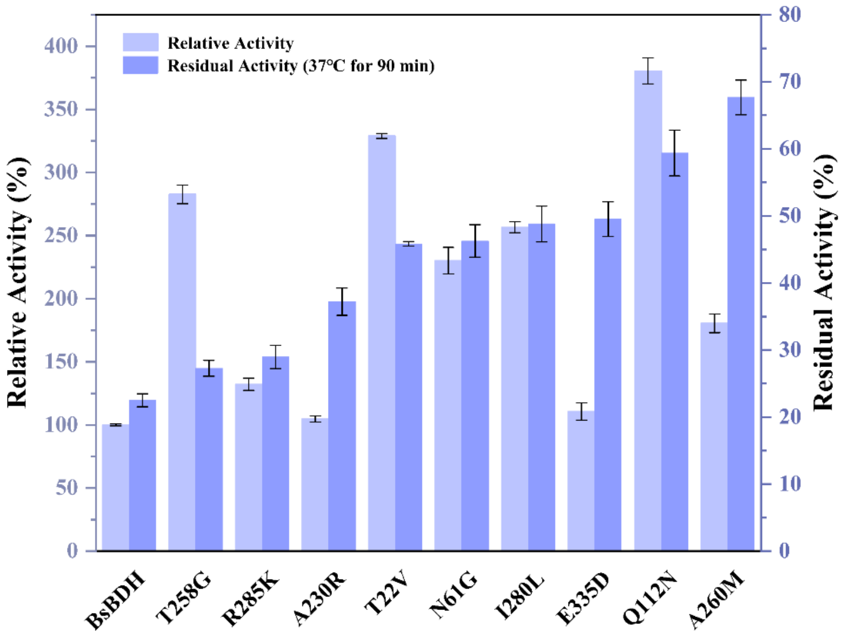

Figure S10. Activity and residual activity of purifed single-point variants improving both activity and thermostability. The data were presented as mean values ± SD from three independent biological replicates (n=3). The error bars represent the standard deviation (SD).

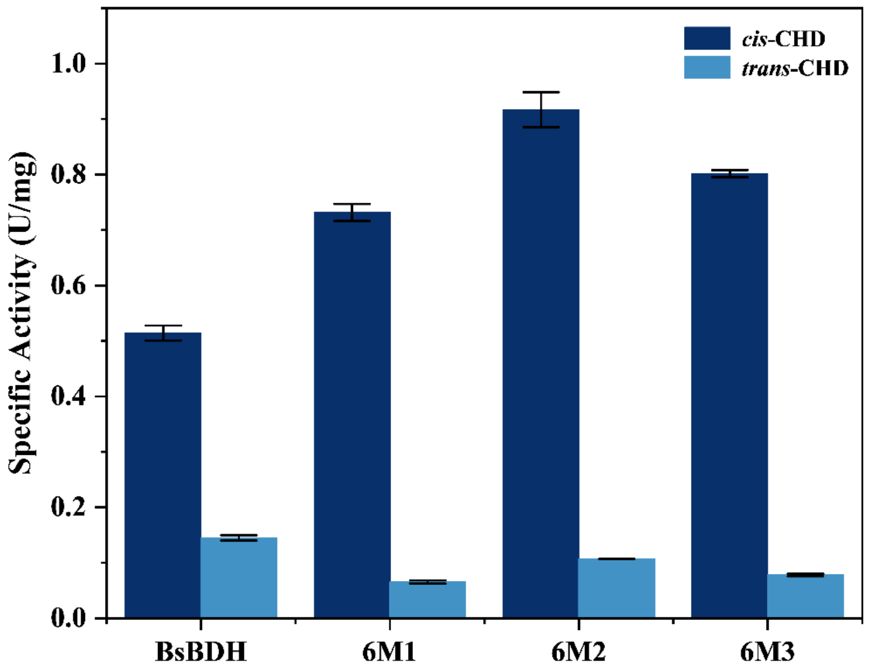

Figure S11. Specific activities of three thermostable variants. The data were presented as mean values ± SD from three independent biological replicates (n=3). The error bars represent the standard deviation (SD).

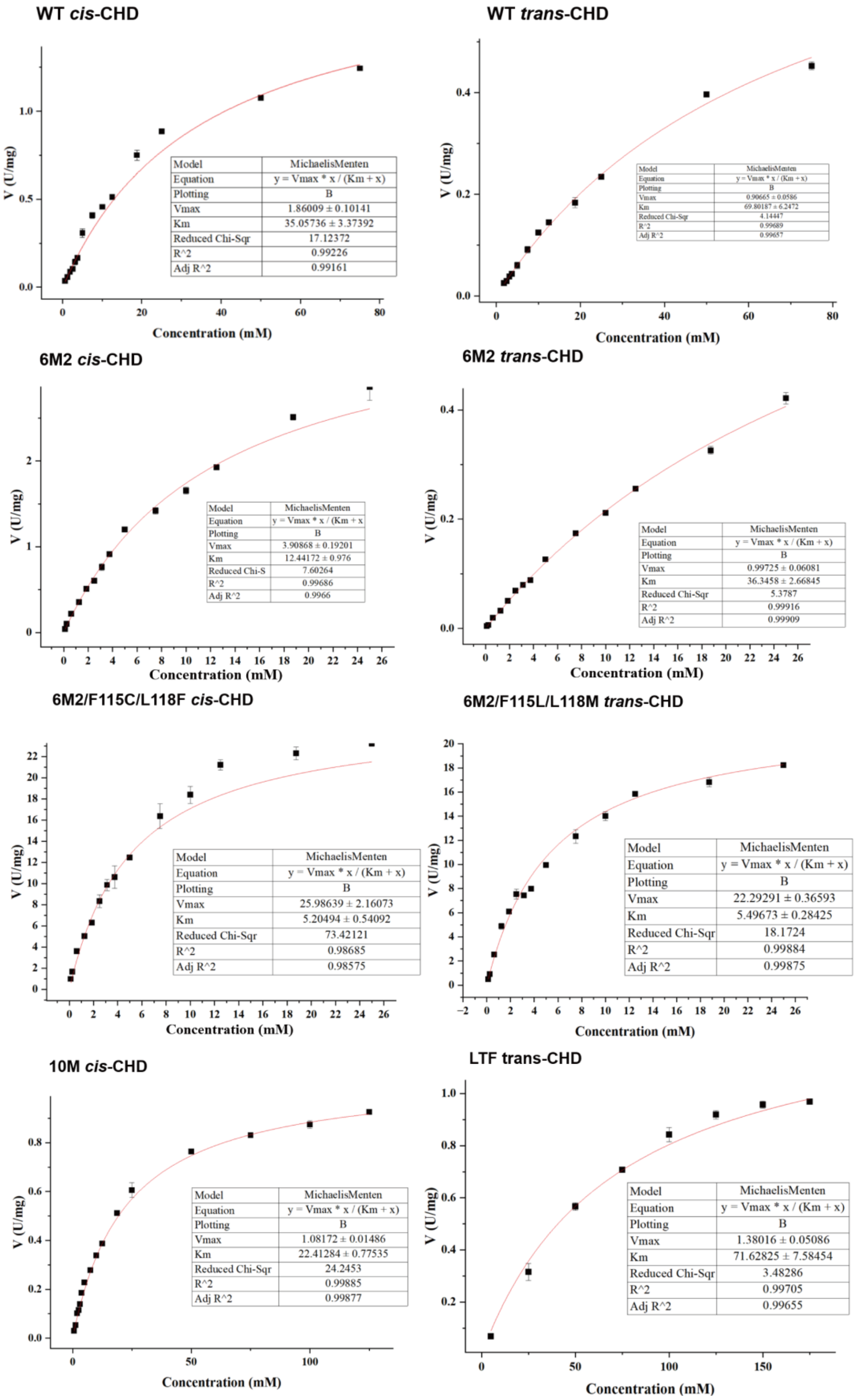

Figure S12. Measurement of kinetic parameters for the wild-type and its mutants.

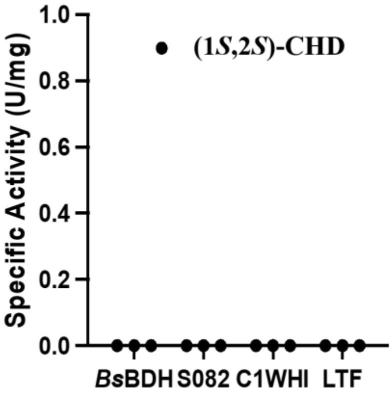

Figure S13. Activities of the wild-type and its mutants against (1*S*, 2*S*)-CHD.

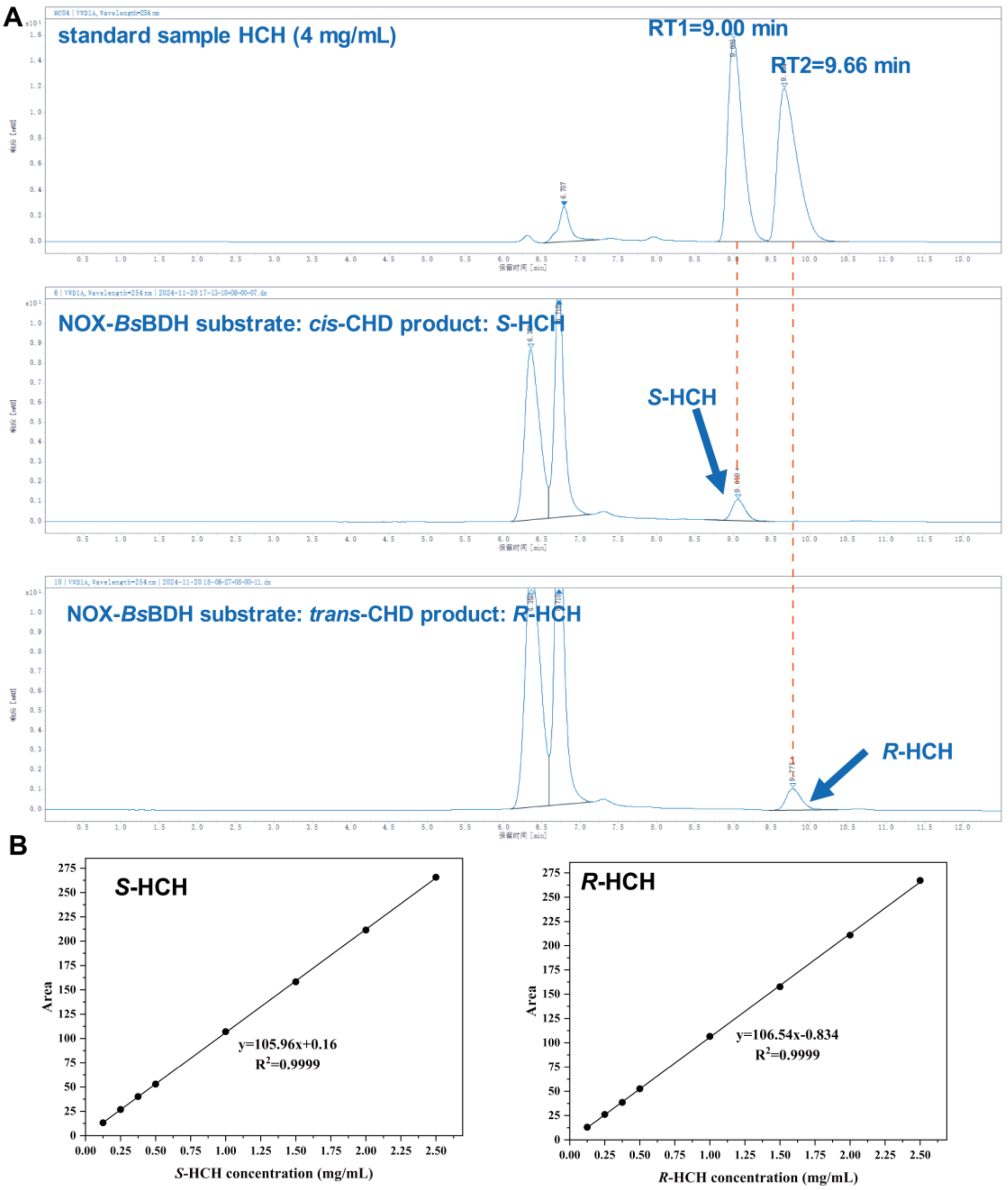

Figure S14. HPLC detection of *S*-HCH and *R*-HCH, and the standard curves.

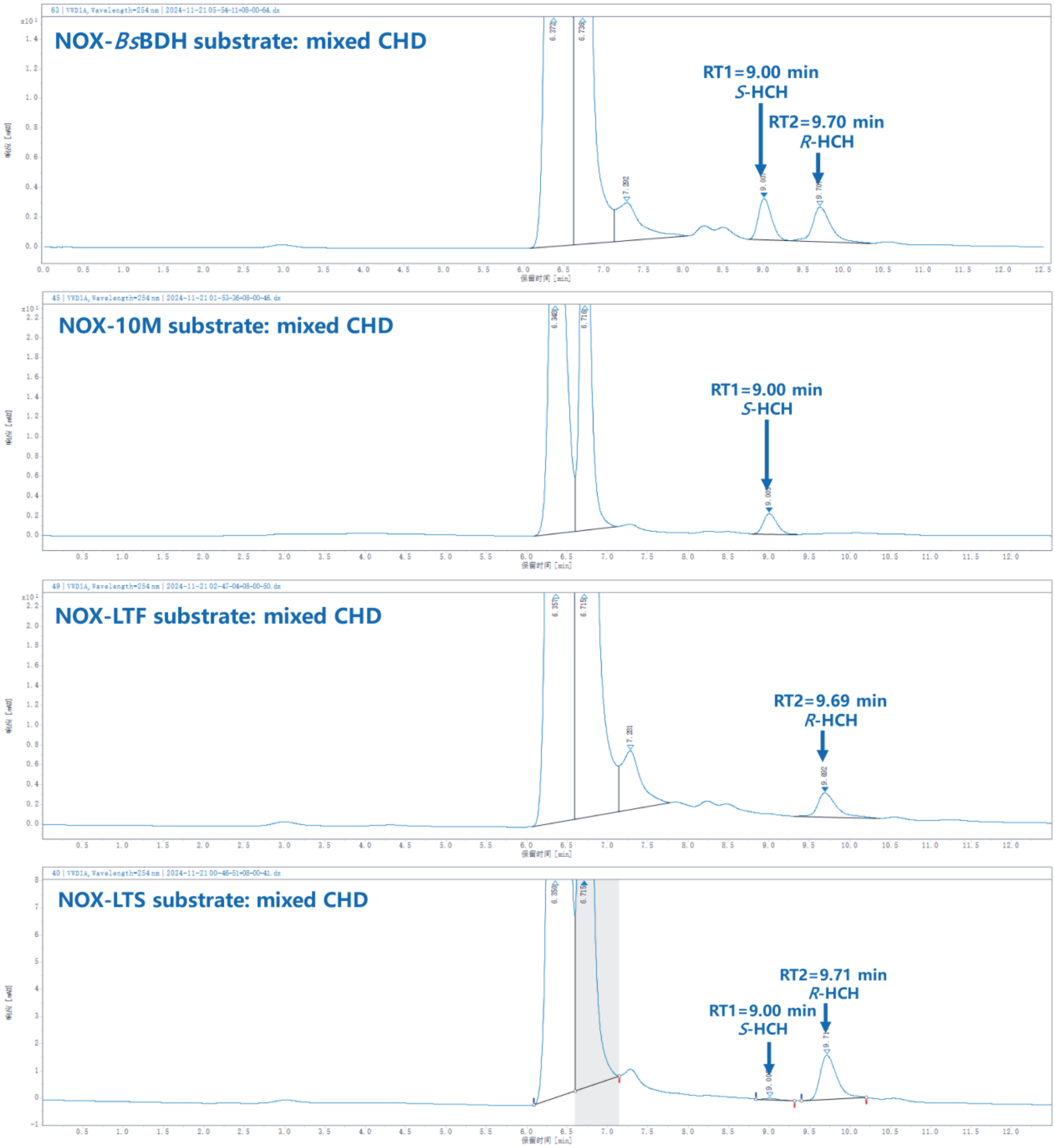

Figure S15. HPLC analysis of target products synthesized by *Bs*BDH and its mutants. The substrate mixed CHD is an equimolar (1:1:1) mixture of *cis*-CHD, (1*R*,2*R*)-CHD, (1*S*,2*S*)-CHD.

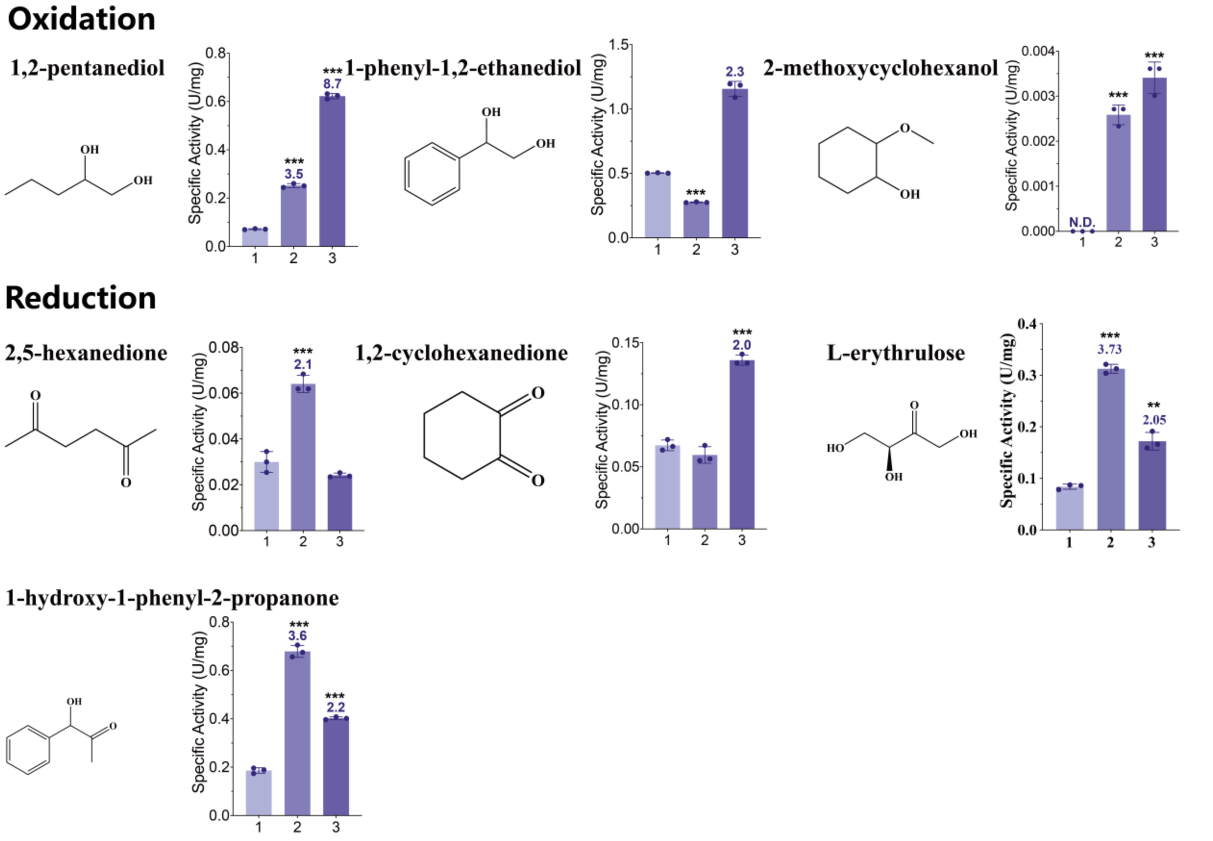

Figure S16. Substrate scope of the mutants 6M2/F115C/L118F (2) and 6M2/F115L/L118MF (3). The asterisks show statistically significant differences from WT (1). The fold increases in activity compared to WT are displayed above the columns. The data were presented as mean values ± SD from three independent biological replicates (n=3). The error bars represent the standard deviation (s.d.). Statistical significance was evaluated using one-way analysis of variance (ANOVA). The asterisks of *, **, *** denote $p < 0.05$, $p < 0.01$ and $p < 0.001$, respectively.

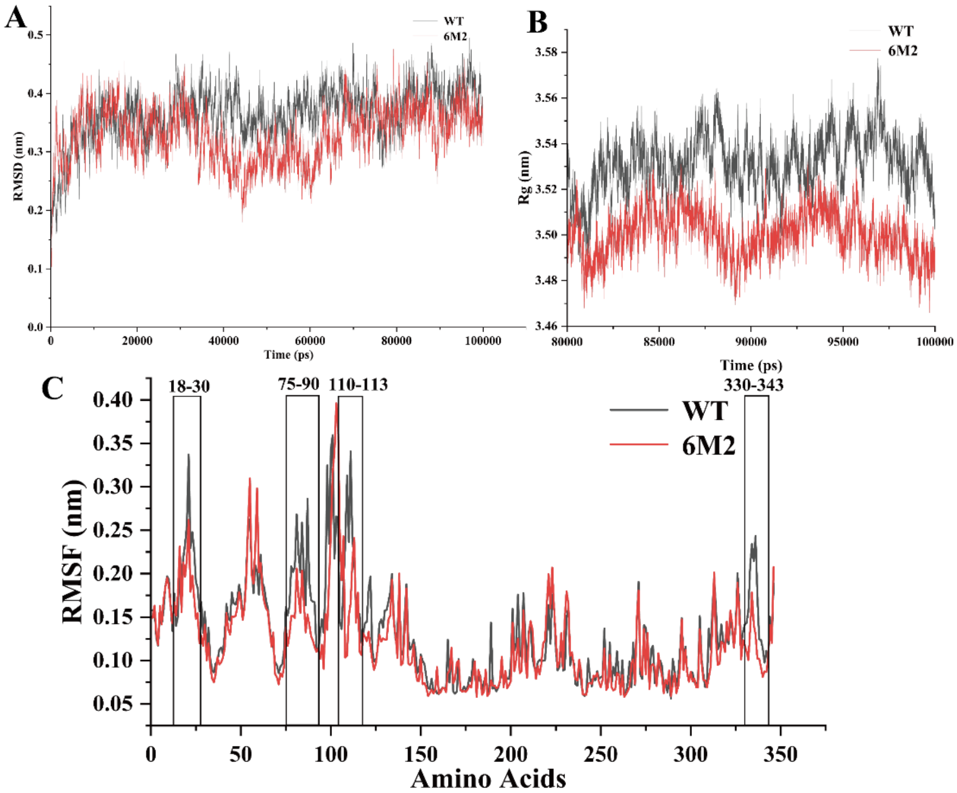

Figure S17. MD simulations analysis of *Bs*BDH and 6M2. A) RMSD analysis. B）Radius of gyration analysis. C) RMSF analysis.

Position 112

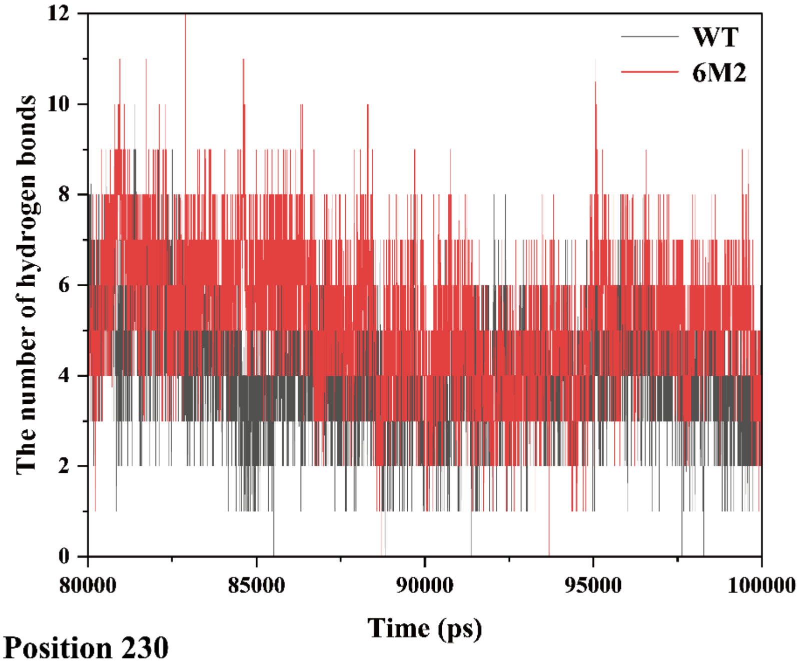

Position 230

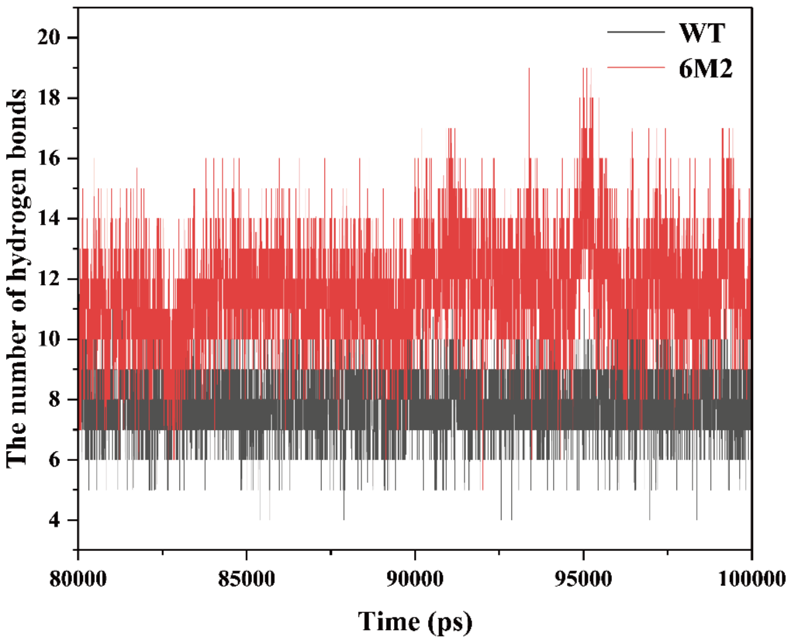

Figure S18. Analysis of the number of hydrogen bonds formed at position 112, 230. The number of hydrogen bonds formed at positions 112 and 230 in the tetrameric structure was calculated separately. In WT, the average numbers of hydrogen bonds formed at positions 112 and 230 with the surrounding residues were 4.16 and 7.82, respectively. In contrast, in 6M2, the average numbers of hydrogen bonds formed at positions 112 and 230 with the surrounding residues were 5.32 and 11.56, respectively.

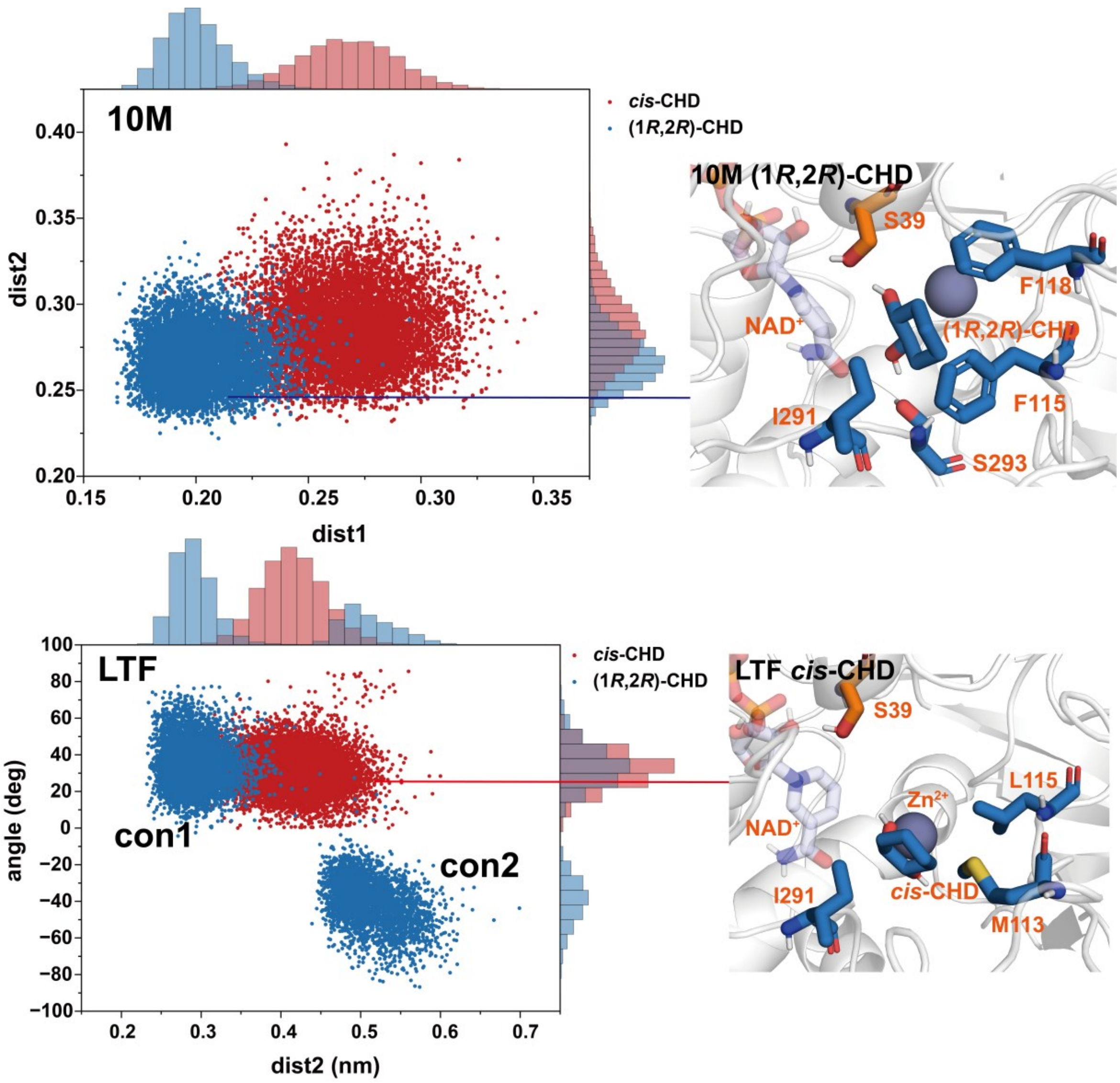

Figure S19. Conformation population analysis of 10M and LTF from the last 10 ns MD trajectories. Parameters dist1, dist2, and angle retain the same geometric definitions as in Figure 6B.

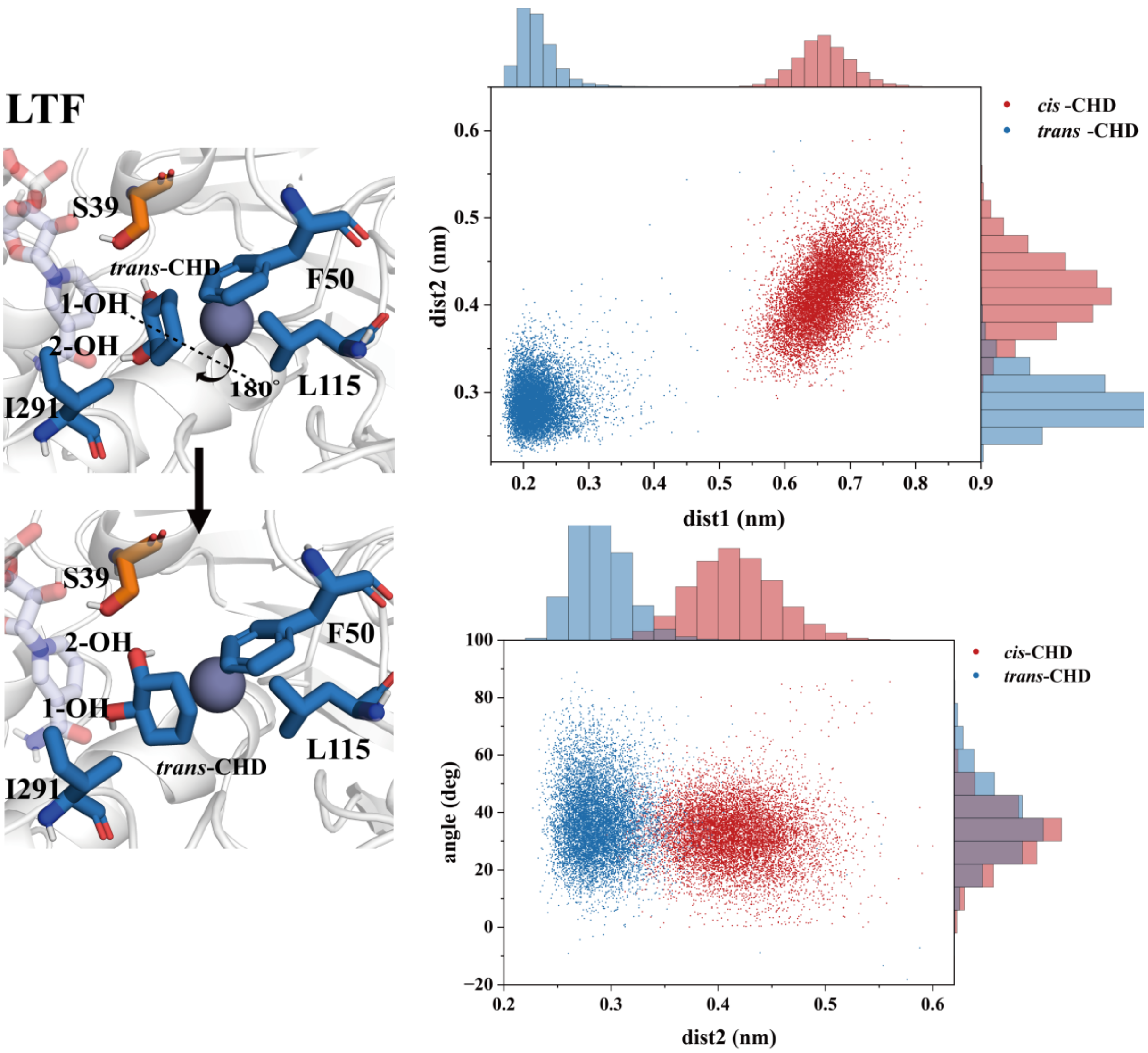

Figure S20. Conformation analysis of LTF with the substrate (1*R*,2*R*)-CHD and conformation population reanalysis from the last 10 ns trajectories. Parameters dist1, dist2, and angle retain the same geometric definitions as in Figure 6B.

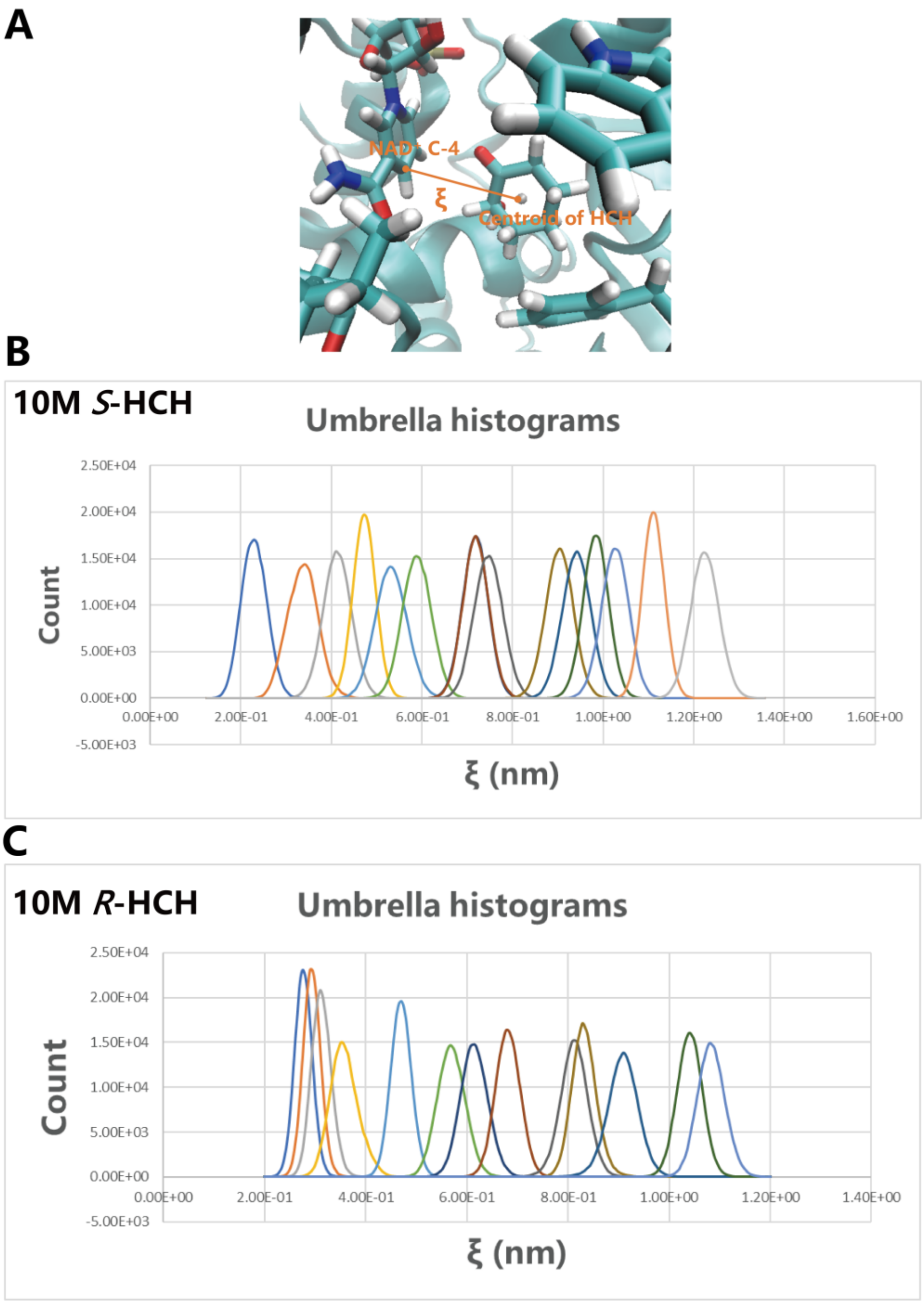

Figure S21. Umbrella sampling of 10M. A) The definition of the reaction coordinate. It is defined as the vector between the centroid of the *R*/*S*-HCH and the C-4 atom of the nicotinamide ring in NAD^+. The pulling simulations were conducted in order to generaate a series of conformations along the reaction coordinate. B) and C) Umbrella histograms. The histograms overlapped with each other to ensure that the PMF can later be derived from these simulations.

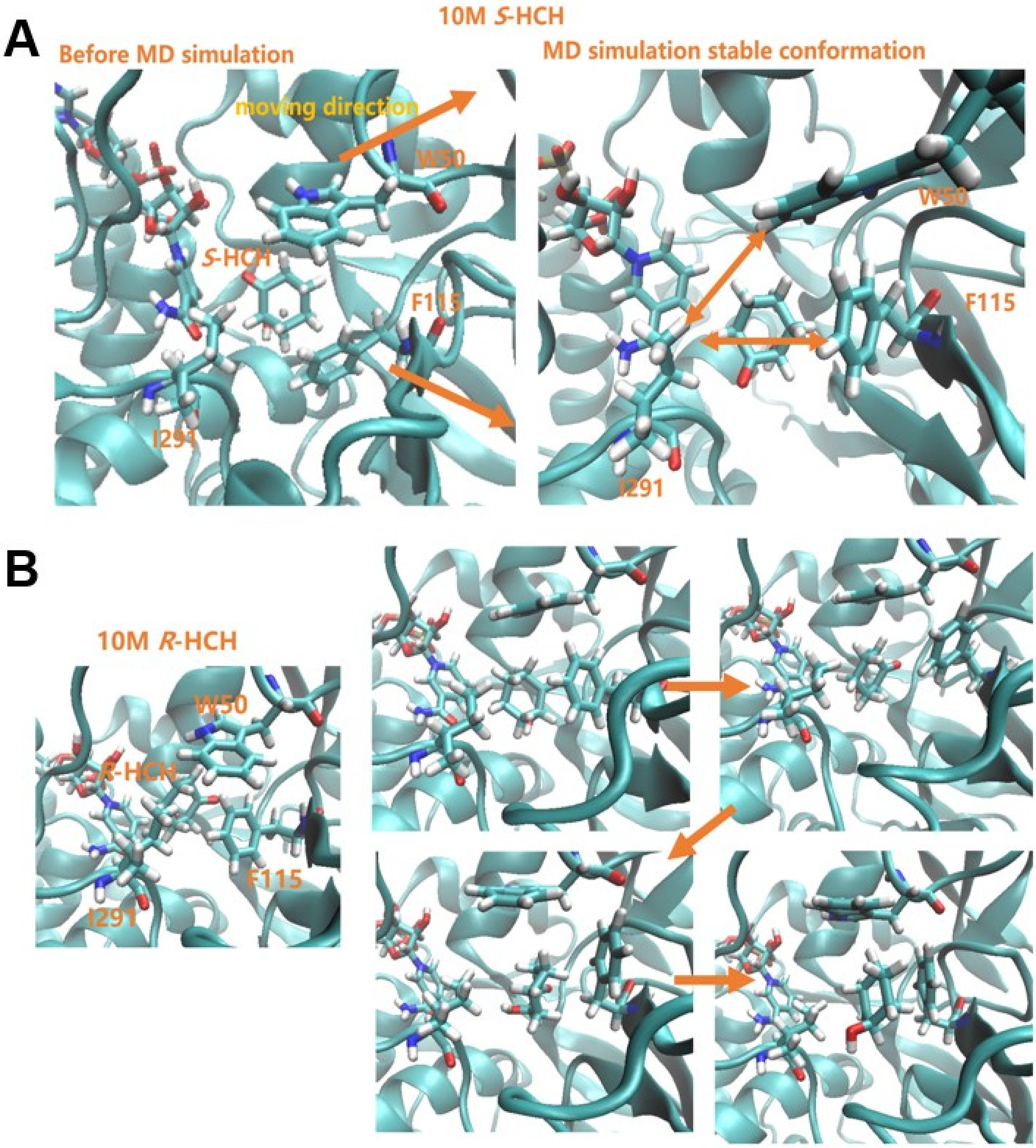

Figure S22. Release of *S*-HCH and *R*-HCH from catalytic center of 10M. A) Release process of *S*-HCH. During the release of *S*-HCH, the distances between W50, F115, and I291 were relatively large, making the substrate release easier. B) Release process of *R*-HCH. *R*-HCH forms strong hydrophobic interactions with W50, F115, and I291. Therefore, the release of *R*-HCH requires more energy to dissociate from residues W50, F115, and I291 to form an unobstructed channel.

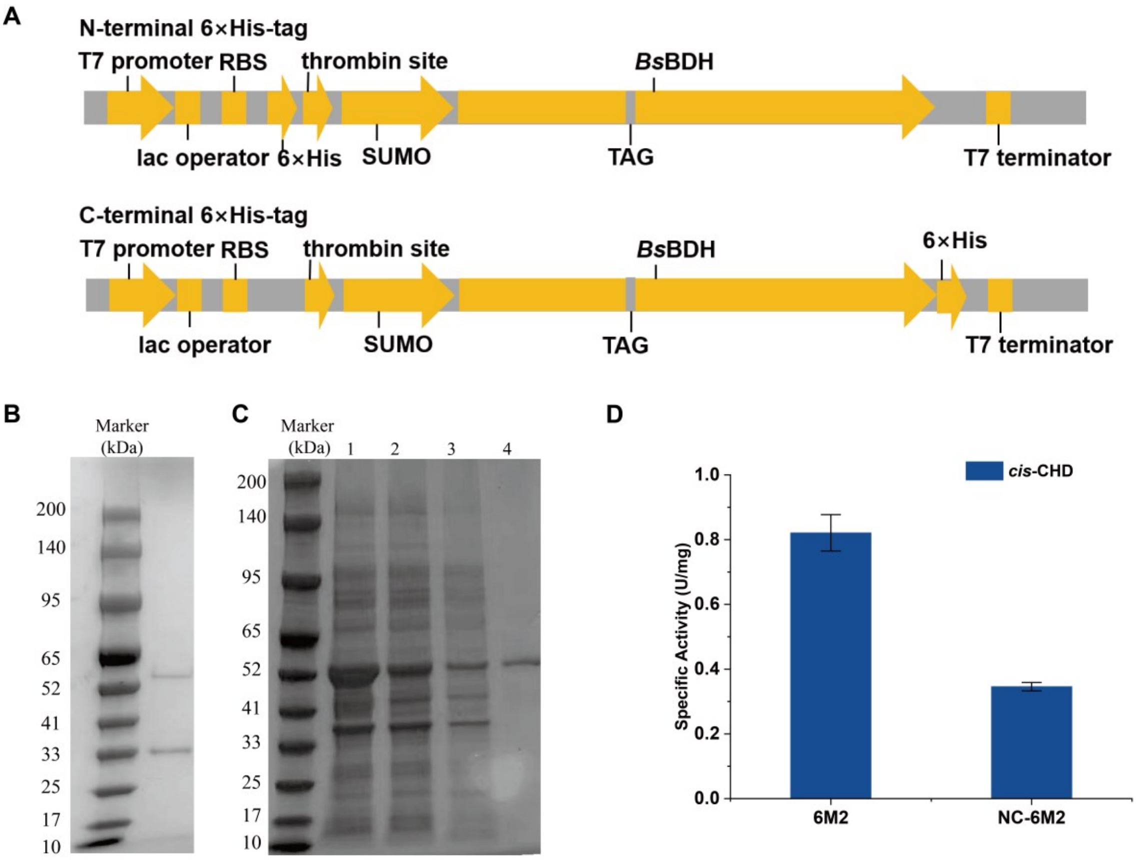

Figure S23. Exchanging His-tag from *N*-terminal to *C*-terminal for purification of whole ncAA-containing proteins. A) Schematic diagram of 6×His-tag alteration. The description of SDS-PAGE: B) *N*-terminal 6×His-tag, C) *C*-terminal 6×His-tag, 1: culture, 2: loading flow-through, 3: washing flow-through, 4: elution flow-through. D) The effects of His-tag position on catalytic activity. The data were presented as mean values ± SD from three independent biological replicates (n=3). The error bars represent the standard deviation (SD).

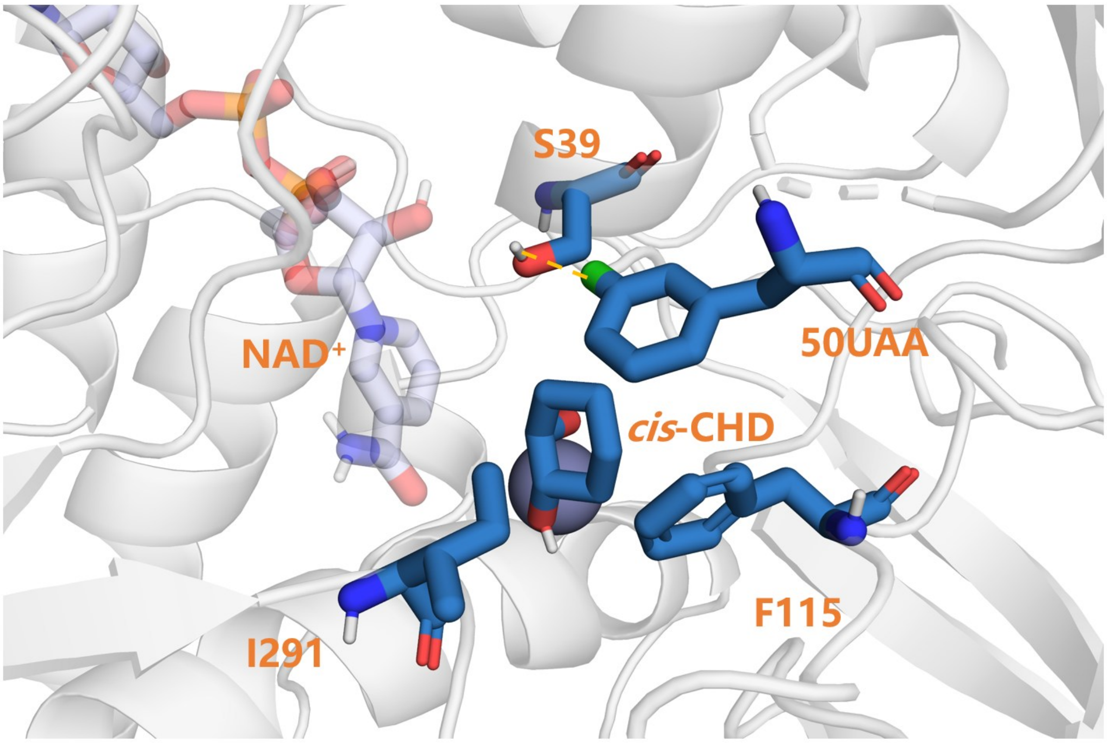

Figure S24. Substrate binding pocket of NC-6M2/Y293S/L118F/F50-3-ClF.

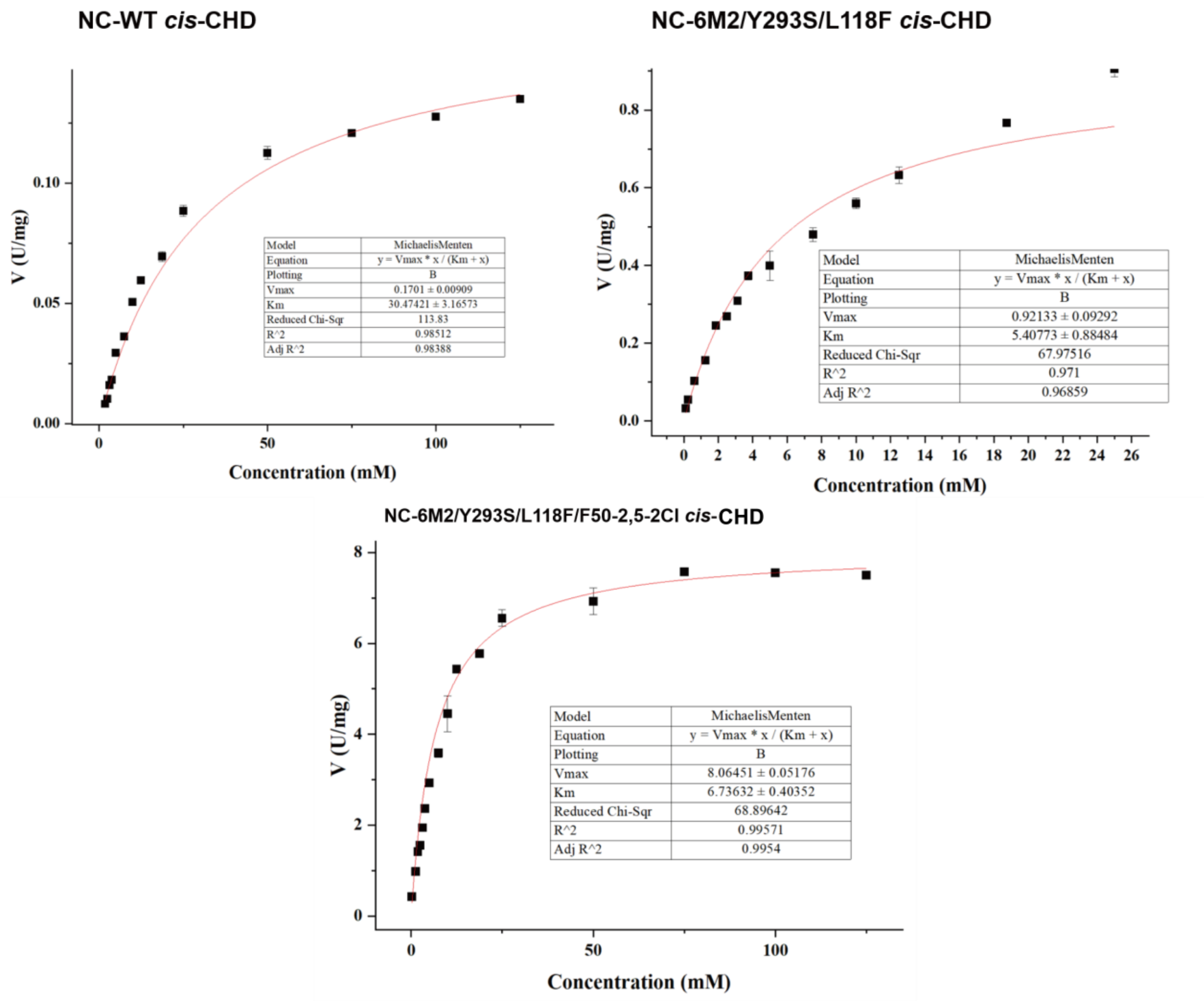

Figure S25. Measurement of kinetic parameters for the wild-type and the ncAA containing mutant.

Supporting Tables

Table S1. Activity and selectivity of single variants predicted by ESM-1v model.

Mutants	Specific activity against *cis*-CHD[a] (U/mg)	Specific activity against *trans*-CHD[b] (U/mg)	Stereoselectivity value[c]
*Bs*BDH	0.152 ± 0.006	0.043 ± 0.001	0.561 ± 0.019
I49A	0.050 ± 0.001	0.028 ± 0.004	0.286 ± 0.064
I49F	0.224 ± 0.001	0.010 ± 0.001	0.916 ± 0.006
I49M	0.121 ± 0.006	0.024 ± 0.003	0.669 ± 0.022
I49T	0.097 ± 0.006	0.017 ± 0.002	0.697 ± 0.010
I49V	0.116 ± 0.001	0.007 ± 0.001	0.893 ± 0.016
F50A	0.002 ± 0.000	0.001 ± 0.000	0.474 ± 0.153
F50L	0.007 ± 0.000	0.006 ± 0.001	0.118 ± 0.073
F50M	0.027 ± 0.002	0.011 ± 0.000	0.403 ± 0.046
F50T	0.000 ± 0.000	0.000 ± 0.000	/
F50W	0.106 ± 0.002	0.001 ± 0.001	0.973 ± 0.018
F50Y	0.004 ± 0.001	0.000 ± 0.000	0.778 ± 0.039
I97L	0.016 ± 0.001	0.007 ± 0.001	0.383 ± 0.073
I97M	0.015 ± 0.002	0.003 ± 0.001	0.663 ± 0.071
I97N	0.000 ± 0.000	0.000 ± 0.000	/
I97Q	0.028 ± 0.006	0.019 ± 0.003	0.190 ± 0.130
I97S	0.032 ± 0.002	0.012 ± 0.002	0.459 ± 0.046
I97T	0.012 ± 0.001	0.002 ± 0.001	0.684 ± 0.123
I97V	0.081 ± 0.007	0.055 ± 0.004	0.189 ± 0.086
I97Y	0.007 ± 0.000	0.003 ± 0.000	0.333 ± 0.040
M113A	0.131 ± 0.003	0.043 ± 0.001	0.508 ± 0.011
M113F	0.009 ± 0.001	0.001 ± 0.000	0.719 ± 0.092
M113G	0.421 ± 0.013	0.154 ± 0.004	0.463 ± 0.026
M113H	0.083 ± 0.005	0.066 ± 0.002	0.112 ± 0.038
M113I	0.727 ± 0.007	0.115 ± 0.001	0.727 ± 0.004
M113L	0.321 ± 0.002	0.098 ± 0.002	0.531 ± 0.010
M113T	0.068 ± 0.003	0.038 ± 0.001	0.282 ± 0.022
M113Y	0.016 ± 0.001	0.008 ± 0.002	0.345 ± 0.132
F115A	0.028 ± 0.002	0.007 ± 0.001	0.617 ± 0.071
F115C	0.401 ± 0.002	0.040 ± 0.001	0.818 ± 0.002
F115G	0.002 ± 0.001	0.000 ± 0.000	0.818 ± 0.288
F115I	0.154 ± 0.001	0.228 ± 0.001	-0.194 ± 0.002
F115L	0.003 ± 0.002	0.059 ± 0.002	-0.897 ± 0.085
F115W	0.013 ± 0.001	0.002 ± 0.001	0.733 ± 0.115
F115Y	0.116 ± 0.010	0.058 ± 0.010	0.333 ± 0.089
L118C	0.038 ± 0.000	0.022 ± 0.001	0.282 ± 0.039
L118F	0.165 ± 0.001	0.052 ± 0.001	0.524 ± 0.005
L118I	0.054 ± 0.001	0.010 ± 0.001	0.692 ± 0.021
L118S	0.000 ± 0.000	0.046 ± 0.000	-1.000 ± 0.000
L118T	0.010 ± 0.001	0.027 ± 0.001	-0.457 ± 0.052
L118V	0.018 ± 0.000	0.025 ± 0.001	-0.167 ± 0.016
L118Y	0.083 ± 0.000	0.035 ± 0.002	0.412 ± 0.036
I268A	0.023 ± 0.004	0.031 ± 0.001	-0.153 ± 0.117
I268C	0.081 ± 0.003	0.039 ± 0.002	0.352 ± 0.027
I268F	0.066 ± 0.003	0.056 ± 0.004	0.077 ± 0.024
I268L	0.034 ± 0.004	0.020 ± 0.002	0.267 ± 0.089
I268T	0.022 ± 0.002	0.001 ± 0.000	0.904 ± 0.012
I268V	0.000 ± 0.000	0.000 ± 0.000	/

I268Y	0.024 ± 0.006	0.044 ± 0.003	-0.294 ± 0.150
W269A	0.305 ± 0.004	0.087 ± 0.014	0.557 ± 0.065
W269F	0.610 ± 0.011	0.136 ± 0.003	0.635 ± 0.013
W269H	0.074 ± 0.002	0.002 ± 0.001	0.960 ± 0.020
W269L	0.028 ± 0.005	0.020 ± 0.002	0.150 ± 0.143
W269T	0.050 ± 0.004	0.068 ± 0.017	-0.154 ± 0.103
W269V	0.214 ± 0.010	0.063 ± 0.006	0.546 ± 0.031
W269Y	0.037 ± 0.002	0.054 ± 0.005	-0.185 ± 0.086
I291A	0.055 ± 0.009	0.074 ± 0.006	-0.146 ± 0.069
I291F	0.273 ± 0.002	0.012 ± 0.001	0.915 ± 0.004
I291H	0.039 ± 0.005	0.003 ± 0.001	0.845 ± 0.040
I291L	0.056 ± 0.003	0.011 ± 0.002	0.680 ± 0.055
I291N	0.028 ± 0.006	0.015 ± 0.004	0.296 ± 0.200
I291T	0.008 ± 0.001	0.002 ± 0.001	0.622 ± 0.158
I291V	0.043 ± 0.000	0.022 ± 0.002	0.335 ± 0.039
Y293A	0.000 ± 0.000	0.000 ± 0.000	/
Y293C	0.000 ± 0.000	0.000 ± 0.000	/
Y293F	0.000 ± 0.000	0.000 ± 0.000	/
Y293H	0.021 ± 0.000	0.009 ± 0.000	0.402 ± 0.024
Y293N	0.000 ± 0.000	0.000657 ± 0.000	-1.000 ± 0.000
Y293S	0.069 ± 0.001	0.003 ± 0.001	0.919 ± 0.021
Y293V	0.000 ± 0.000	0.000 ± 0.000	/

[a] *cis*-CHD consists of the *meso*-CHD configuration.
[b] *trans*-CHD comprises two configurations including (1*S*,2*S*)-CHD and (1*R*,2*R*)-CHD.
[c] Stereoselectivity value was calculated as $(SA_{cis}-SA_{trans})/(SA_{cis}+SA_{trans})$, where SA_{cis} and SA_{trans} represent specific activities against *cis*-CHD and *trans*-CHD, respectively.

Table S2. Thermostable singe variants predicted by the GRAPE and PROSS strategy.

**Method**	**Mutants**
**GRAPE**	
Rosetta_ddg	P153A, E152L, D40L, H70V, P298W, H276D, C37H, S130L, G289Y, L118F, G330W, S39M, G46W, R285L, E335D, Q252W, H42W, E71Q, F297L, K271W, K283Y, V156Y, S202I, K340I, I275W, Y293H
ABACUS	P200D, K32L, E242D, V243C, Q338I, T263V, Y86L, Q206A, R285K, M68L, W6L
FoldX	G178D, D132P, S154M, A260N, D195Y, T263I, S202D, D195M, N278W, S130V, A260L, R205F, A260M, T258G
**PROSS**	
Rosetta_cartesian ref_2015cart	S154A, L135M, H16D, G330E, F137H, A230R, T258R, A260G, I259K, E84T, S163Q, G46H, L301I, G272E, C177A, V248A, T22V, K305S, I280L, A168P, H276N, D295N, Y86F, V320K, T67I, R251T, Q112N, P298E, E75V, E306Q, D132P, Y159H, Y145F, T194S, S302E, Q207K, N8G, N61G, K21Q, I282L, G25N, E142N, D312E, D223E, A299E

Table S3. Activity and residual activity of 34 predicted stabilizing and 34 predicted destabilizing mutants deaigned by the PASS strategy. The specific activity of *Bs*BDH against (2*R*,3*R*)-butanediol was 1.03 ± 0.02 U/mg.

Mutants	Relative activity (%)	Residual activity (%)
**Predicted stabilizing mutants**		
*Bs*BDH	100.0 ± 1.5	20.7 ± 0.4
A230R	386.6 ± 7.4	37.6 ± 0.6
A260M	417.2 ± 10.3	40.4 ± 1.9
A299E	14.9 ± 1.3	6.8 ± 0.0
D132P	71.8 ± 1.4	27.1 ± 0.4
D223E	87.8 ± 1.4	25.5 ± 0.0
E142N	42.6 ± 1.0	59.9 ± 1.6
E200D	9.9 ± 0.8	23.1 ± 3.3
E335D	241.4 ± 2.0	52.3 ± 2.5
E84T	49.7 ± 1.1	58.9 ± 0.4
F137H	97.6 ± 4.7	55.2 ± 1.8
H276N	78.1 ± 1.0	34.3 ± 1.1
I259K	60.4 ± 0.2	94.0 ± 1.2
I280L	146.6 ± 13.4	30.7 ± 4.1
K21Q	66.6 ± 2.1	34.9 ± 0.5
K283Y	3.9 ± 0.3	11.1 ± 3.8
K305S	9.9 ± 3.8	44.4 ± 0.0
L301I	42.1 ± 1.7	56.6 ± 2.0
M68L	29.0 ± 1.7	45.2 ± 0.6
N61G	104.0 ± 2.0	43.8 ± 0.7
P298E	3.5 ± 0.0	12.2 ± 0.7
Q112N	121.5 ± 1.8	57.2 ± 3.4
Q206A	41.4 ± 2.4	28.0 ± 1.3
R285K	115.6 ± 1.3	47.7 ± 0.9
S130L	12.0 ± 0.3	40.3 ± 1.1
S154M	2.3 ± 0.6	0.0 ± 0.0
T22V	417.9 ± 31.0	81.0 ± 7.5
T258G	128.7 ± 3.2	70.4 ± 1.6
T263I	74.0 ± 6.5	44.1 ± 4.8
T67I	82.1 ± 0.3	30.8 ± 0.7
V320K	71.2 ± 2.4	32.0 ± 0.9
W6L	0.5 ± 0.1	79.9 ± 1.2
Y145F	94.1 ± 1.0	27.5 ± 0.5
Y159H	40.0 ± 0.3	51.8 ± 0.9
Y86F	32.7 ± 0.3	58.2 ± 1.6
**Predicted destabilizing mutants**		
A168P	30.3 ± 1.4	13.6 ± 0.0
A260G	47.1 ± 3.8	14.9 ± 0.0
A260L	73.4 ± 1.6	19.1 ± 0.2
A260N	59.1 ± 8.7	14.3 ± 0.0
D195L	47.0 ± 6.5	4.5 ± 2.3

D195Y	83.3 ± 1.5	8.1 ± 1.8
E242D	9.2 ± 2.5	26.6 ± 2.7
G25N	28.2 ± 0.7	5.1 ± 0.6
G272E	68.5 ± 5.1	19.1 ± 1.9
G330W	12.0 ± 0.3	8.9 ± 0.6
H16D	68.6 ± 1.3	9.9 ± 0.7
H276D	61.1 ± 2.3	13.9 ± 0.0
I275W	7.1 ± 0.5	7.7 ± 0.0
K271W	23.9 ± 1.4	11.0 ± 1.1
K32C	40.3 ± 4.3	2.0 ± 0.0
K340I	0.5 ± 0.1	0.0 ± 0.0
L135M	76.8 ± 1.1	18.1 ± 0.5
N278W	3.2 ± 0.7	0.0 ± 0.0
N8G	84.3 ± 1.2	33.9 ± 1.5
P153A	35.2 ± 1.4	0.0 ± 0.0
Q207K	45.7 ± 0.3	12.4 ± 0.0
Q252W	262.2 ± 27.2	16.3 ± 1.0
Q338I	26.4 ± 5.5	31.0 ± 2.7
R205F	2.4 ± 0.0	0.0 ± 0.0
R285C	6.5 ± 0.5	8.3 ± 0.0
S154A	122.1 ± 17.2	17.6 ± 0.0
S163Q	107.6 ± 3.4	16.9 ± 0.7
S202D	71.5 ± 4.3	5.7 ± 0.8
S202I	23.2 ± 1.9	30.1 ± 3.6
S302E	17.3 ± 1.3	8.3 ± 0.0
V243C	73.4 ± 6.6	14.0 ± 0.5
V248A	60.6 ± 10.5	15.2 ± 4.0
Y293H	8.1 ± 0.5	4.1 ± 0.0
Y86L	47.0 ± 0.8	19.5 ± 0.0

Table S4. Half-life times of combinatorial mutants at 37 ºC.

**Mutant**	**Half-life time (min)**
BsBDH	36.1
A230R	81.9
A260M	122.0
E335D	180.5
I280L	111.2
N61G	144.4
Q112N	113.4
R285K	48.1
T22V	222.1
T258G	49.6
A230R/A260M	154.7
A230R/E335D	105.5
A230R/I280L	108.8
A230R/R285K	85.5
A230R/T258G	96.6
A260M/I280L	230.2
A260M/R285K	72.1
I280L/E335D	269.7
N61G/A230R	129.3
N61G/A260M	149.3
N61G/Q112N	113.0
N61G/R285K	86.9
N61G/T258G	164.2
Q112N/A230R	50.0
Q112N/A260M	176.8
Q112N/E335D	172.8
Q112N/I280L	178.6
Q112N/R285K	45.3
Q112N/T258G	66.5
R285K/E335D	168.6
T22V/A230R	112.1
T22V/A260M	104.0
T22V/E335D	69.3
T22V/I280L	156.8
T22V/N61G	76.9
T22V/Q112N	61.6
T22V/R285K	45.9
T258G/E335D	163.0
T258G/R285K	51.9
A230R/A260M/E335D	91.2
A230R/A260M/I280L	161.2
A230R/I280L/E335D	141.5
A230R/I280L/R285K	99.0
A230R/T258G/I280L	113.6
A260M/R285K/E335D	96.3
N61G/A230R/E335D	147.5
N61G/A230R/R285K	79.7

N61G/Q112N/E335D	96.3
N61G/R285K/E335D	91.2
N61G/T258G/R285K	100.5
Q112N/I280L/E335D	106.6
T22V/A230R/A260M	76.2
T22V/A230R/T258G	87.7
T22V/I280L/E335D	130.8
T22V/N61G/Q112N	73.0
T258G/I280L/R285K	64.2
T258G/R285K/E335D	130.8
A230R/T258G/A260M/I280L	277.3
A230R/T258G/A260M/R285K	141.5
A230R/T258G/I280L/E335D	288.8
A230R/T258G/I280L/R285K	135.9
N61G/A230R/A260M/E335D	385.0
N61G/A230R/I280L/R285K	70.0
N61G/A230R/R285K/E335D	113.6
N61G/A230R/T258G/A260M	157.5
N61G/A230R/T258G/I280L	182.4
N61G/A260M/R285K/E335D	231.0
N61G/I280L/R285K/E335D	161.2
N61G/Q112N/A230R/E335D	157.5
N61G/Q112N/A230R/I280L	247.6
N61G/Q112N/A260M/R285K	147.5
N61G/Q112N/I280L/R285K	157.5
N61G/Q112N/T258G/A260M	256.7
N61G/Q112N/T258G/R285K	141.5
N61G/T258G/A260M/I280L	169.1
Q112N/I280L/R285K/E335D	99.0
T22V/A230R/A260M/E335D	223.6
T22V/A230R/I280L/R285K	79.7
T22V/A230R/T258G/E335D	150.7
T22V/A230R/T258G/R285K	59.2
T22V/A260M/I280L/E335D	130.8
T22V/A260M/R285K/E335D	113.6
T22V/N61G/Q112N/A230R	407.7
T22V/N61G/Q112N/I280L	100.5
T22V/N61G/Q112N/T258G	141.5
T22V/N61G/T258G/E335D	150.7
T22V/N61G/T258G/R285K	198.0
T22V/Q112N/A230R/A260M	147.5
T22V/Q112N/A260M/E335D	147.5
T22V/Q112N/I280L/E335D	301.4
T22V/Q112N/I280L/R285K	55.0
T22V/Q112N/T258G/I280L	90.0
T22V/T258G/I280L/E335D	90.0
T258G/A260M/I280L/E335D	182.4
T258G/A260M/I280L/R285K	106.6
A230R/T258G/A260M/I280L/E335D	147.5

N61G/A230R/A260M/I280L/R285K	223.6
N61G/Q112N/A230R/I280L/E335D	462.0
N61G/Q112N/A230R/T258G/A260M	315.1
N61G/Q112N/A260M/R285K/E335D	126.0
N61G/Q112N/T258G/A260M/E335D	301.4
N61G/Q112N/T258G/R285K/E335D	198.0
Q112N/A230R/T258G/A260M/E335D	301.4
Q112N/T258G/A260M/R285K/E335D	173.3
Q112N/T258G/I280L/R285K/E335D	105.0
T22V/A230R/A260M/R285K/E335D	247.6
T22V/A230R/T258G/A260M/I280L	223.6
T22V/A230R/T258G/A260M/R285K	75.3
T22V/A230R/T258G/I280L/E335D	165.0
T22V/A230R/T258G/R285K/E335D	154.0
T22V/A260M/I280L/R285K/E335D	182.4
T22V/N61G/A230R/I280L/R285K	126.0
T22V/N61G/A230R/T258G/E335D	277.3
T22V/N61G/I280L/R285K/E335D	161.2
T22V/N61G/Q112N/A260M/I280L	462.0
T22V/N61G/Q112N/I280L/E335D	173.3
T22V/N61G/Q112N/T258G/A260M	247.6
T22V/N61G/Q112N/T258G/I280L	154.0
T22V/N61G/T258G/A260M/E335D	216.6
T22V/Q112N/A230R/A260M/E335D	315.1
T22V/Q112N/A230R/R285K/E335D	154.0
T22V/Q112N/T258G/A260M/E335D	169.1
T22V/Q112N/T258G/I280L/E335D	422.6
T22V/Q112N/T258G/R285K/E335D	130.8
T22V/T258G/I280L/R285K/E335D	157.5
N61G/A230R/T258G/I280L/R285K/E335D	135.9
N61G/Q112N/A230R/A260M/I280L/E335D	223.6
N61G/Q112N/A230R/T258G/A260M/R285K	157.5
N61G/Q112N/A260M/I280L/R285K/E335D	105.0
N61G/Q112N/T258G/A260M/I280L/E335D	182.4
N61G/Q112N/T258G/A260M/R285K/E335D	113.6
Q112N/A230R/T258G/I280L/R285K/E335D	154.0
T22V/A230R/T258G/A260M/I280L/E335D	315.1
T22V/N61G/A230R/I280L/R285K/E335D	330.1
T22V/N61G/A230R/T258G/A260M/I280L (6M3)	495.1
T22V/N61G/Q112N/A230R/A260M/E335D	161.2
T22V/N61G/Q112N/A230R/A260M/I280L (6M1)	1033.7
T22V/N61G/Q112N/A230R/A260M/R285K	113.6
T22V/N61G/Q112N/T258G/A260M/E335D	346.6
T22V/Q112N/A230R/A260M/I280L/R285K	135.9
T22V/Q112N/A230R/T258G/A260M/I280L (6M2)	533.1
N61G/Q112N/A230R/T258G/A260M/I280L/E335D	288.8
N61G/Q112N/A230R/T258G/I280L/R285K/E335D	433.2
T22V/N61G/A230R/T258G/I280L/R285K/E335D	150.7
T22V/N61G/Q112N/A230R/A260M/I280L/R285K	169.1

T22V/N61G/Q112N/A230R/A260M/R285K/E335D	239.0
T22V/N61G/Q112N/A230R/I280L/R285K/E335D	115.5
T22V/N61G/Q112N/T258G/A260M/R285K/E335D	310.0
T22V/Q112N/T258G/A260M/I280L/R285K/E335D	247.6
N61G/Q112N/A230R/T258G/A260M/I280L/R285K/E335D	330.1
T22V/N61G/Q112N/A230R/T258G/I280L/R285K/E335D	491.5
T22V/N61G/Q112N/A230R/T258G/A260M/I280L/R285K/E335D	277.3
